# The association between reallocations of time and health using compositional data analysis: a systematic scoping review with an interactive data exploration interface

**DOI:** 10.1186/s12966-023-01526-x

**Published:** 2023-10-19

**Authors:** Aaron Miatke, Tim Olds, Carol Maher, Francois Fraysse, Maddison L Mellow, Ashleigh E Smith, Zeljko Pedisic, Jozo Grgic, Dorothea Dumuid

**Affiliations:** 1 Alliance for Research in Exercise, Nutrition and Activity, Allied Health and Human Performance, University of South Australia, GPO box, Adelaide, S.A 2471, 5001 Australia; 2 Centre for Adolescent Health, Murdoch Children’s Research Institute, Melbourne, Australia; 3 Institute for Health and Sport, Victoria University, Melbourne, Australia

**Keywords:** Physical activity, Moderate-to-vigorous physical activity, Sedentary behaviour, Sleep, Compositional data analysis

## Abstract

**Background:**

How time is allocated influences health. However, any increase in time allocated to one behaviour must be offset by a decrease in others. Recently, studies have used compositional data analysis (CoDA) to estimate the associations with health when reallocating time between different behaviours. The aim of this scoping review was to provide an overview of studies that have used CoDA to model how reallocating time between different time-use components is associated with health.

**Methods:**

A systematic search of four electronic databases (MEDLINE, Embase, Scopus, SPORTDiscus) was conducted in October 2022. Studies were eligible if they used CoDA to examine the associations of time reallocations and health. Reallocations were considered between movement behaviours (sedentary behaviour (SB), light physical activity (LPA), moderate-to-vigorous physical activity (MVPA)) or various activities of daily living (screen time, work, household chores etc.). The review considered all populations, including clinical populations, as well as all health-related outcomes.

**Results:**

One hundred and three studies were included. Adiposity was the most commonly studied health outcome (n = 41). Most studies (n = 75) reported reallocations amongst daily sleep, SB, LPA and MVPA. While other studies reported reallocations amongst sub-compositions of these (work MVPA vs. leisure MVPA), activity types determined by recall (screen time, household chores, passive transport etc.) or bouted behaviours (short vs. long bouts of SB). In general, when considering cross-sectional results, reallocating time to MVPA from any behaviour(s) was favourably associated with health and reallocating time away from MVPA to any behaviour(s) was unfavourably associated with health. Some beneficial associations were seen when reallocating time from SB to both LPA and sleep; however, the strength of the association was much lower than for any reallocations involving MVPA. However, there were many null findings. Notably, most of the longitudinal studies found no associations between reallocations of time and health. Some evidence also suggested the context of behaviours was important, with reallocations of leisure time toward MVPA having a stronger favourable association for health than reallocating work time towards MVPA.

**Conclusions:**

Evidence suggests that reallocating time towards MVPA from any behaviour(s) has the strongest favourable association with health, and reallocating time away from MVPA toward any behaviour(s) has the strongest unfavourable association with health. Future studies should use longitudinal and experimental study designs, and for a wider range of outcomes.

**Supplementary Information:**

The online version contains supplementary material available at 10.1186/s12966-023-01526-x.

## Introduction

Time is a finite resource, and how it is allocated may have a significant impact on health. For example, participation in physical activity [1] (PA) and adequate sleep duration [2] have long been associated with many favourable health outcomes. Conversely, high levels of sedentary behaviour [3] (SB), particularly recreational screen time [4], is thought to have a negative association with health. Historically, the influence of each of these behaviours on health has been studied in isolation. However, given that the duration of each day is fixed, any increase in time spent in one behaviour must be offset by a decrease in others. This has led to a growing interest in understanding how reallocating time between different behaviours can affect health.

Isotemporal substitution models were first proposed by Mekary et al. (2009) to estimate the effect of reallocating a fixed amount of time spent in one behaviour to another; for example, reallocating 30 min of SB to moderate-to-vigorous PA (MVPA). More recently, researchers have started using compositional data analysis (CoDA) to perform isotemporal substitutions [5, 6]. While the goal of these different approaches is the same (to investigate the associations of reallocating time between different time-use components), the way they treat the time-use variables differs. Traditional isotemporal substitution models assume time-use variables have an absolute nature. The models include all components, bar one, while also controlling for total wear time. CoDA time-reallocation models, however, assume that time-use variables are compositional data, and therefore convey relative information. CoDA models therefore include isometric log-ratios of parts [5, 7, 8]. These differences in the treatment of data mean that findings from CoDA-based time-reallocation studies can differ from those using Mekary et al. (2009) isotemporal substitution model [9] (e.g., they may be asymmetrical and time reallocations are considered relative to a reference composition [5, 7, 10]). Along with the traditional ‘one-for-one’ substitutions (i.e., reallocating a fixed amount of time between two behaviours only; e.g., sleep for SB), several CoDA studies have used a ‘one-for-remaining’ reallocation model [11]. With this method, the time reallocated to one behaviour is drawn ‘pro rata’ from all the other time-use components. CoDA-based models allow various reallocations of time between behaviours to be examined, from different reference time-use compositions [12].

A 2018 scoping review of studies using isotemporal substitution models found that reallocations of time between sleep, SB, light PA (LPA) and MVPA were associated with a range of health outcomes [13]. However, only three of the 56 studies included in the review used a CoDA-based model. It is therefore unknown how the findings of the review would have differed, if it had considered only studies that have used compositional isotemporal substitution models. Since then, there has been a rapid increase in the number of studies that have employed CoDA to examine how reallocations of time between different behaviours are associated with various health outcomes and in different populations [14–16]. The recent GRANADA consensus statement has also endorsed the use of CoDA as a suitable way of accounting for the inherent co-dependency of time-use data and suggested it was appropriate for analysing the effects of reallocating time between different time-use components [17]. Hence, an updated review of CoDA-based time-reallocation studies is warranted.

Therefore, the aim of this scoping review was to provide an overview of studies that have used CoDA to model how reallocating time between different time-use components is associated with health. The three specific objectives of the scoping review were to: [1] review and summarise findings from such studies; [2] describe their study designs, samples, health outcomes, time-use behaviours and types of reallocations investigated; and [3] identify research gaps.

## Methods

This systematic scoping review was undertaken using the framework outline by Peters et al. (2015), in accordance with the Joanna Briggs Institute methodology for scoping reviews. A scoping review methodology was chosen given the considerable heterogeneity anticipated among the included studies with regard to the populations studied, health outcomes, time-use behaviours and types of reallocations.

### Protocol

A protocol for this review was developed *a priori* and registered in advance on the Open Science framework [18]. The reporting in this scoping review was done according to the Preferred Reporting Items for Systematic Reviews and Meta-Analyses extension for Scoping Reviews (PRISMA-ScR) Checklist [19](Additional file 1).

### Eligibility criteria

To facilitate the search process, the Participants, Concept, Context (PCC) framework was used as recommended for scoping reviews [20] as follows: “Participants”: all populations were considered, including clinical populations; “Concept”: changes in health outcomes associated with reallocations of time determined using compositional data analysis, where time use referred to time spent in movement behaviours (e.g. sleep, SB, LPA, MVPA) as well as time spent in various activities of daily living (e.g. work, household chores, leisure activities), while studies focused on time spent in different postures and levels of muscle activation were excluded (for example, a small number of occupational studies investigated reallocations of time between levels of arm elevation [21] or forward bending [22]); and “Context”: all health-related outcomes were considered. Only original, empirical studies published in English in peer-reviewed sources were included. Systematic reviews, meta-analyses, methods papers, and opinion papers were not included in this review.

### Search strategy

A search strategy was developed in consultation with an academic librarian, to identify search terms and subject headings related to time-use components and CoDA. The following databases were searched: MEDLINE (through Ovid), Embase (through Ovid), Scopus, and SPORTDiscus (through EBSCOhost), with a date limit set to only include papers published since 2015, as this is the date of the first known paper to study reallocations of time using CoDA [6]. Searches were run on the 13th of October 2022. The search syntax for the MEDLINE platform is outlined is provided in Additional file 2.

### Study selection

After the removal of duplicates, records were imported into Covidence (Veritas Health Innovation, Melbourne, Australia) for screening. Firstly, titles and abstracts were screened for relevance. Articles deemed to be potentially relevant then proceeded to the second stage ─ full-text review. Both stages of study selection were undertaken in duplicate (AM, DD, AS, MM), with any discrepancies resolved via discussion. In addition, screening of reference lists of all included studies was undertaken to identify any additional relevant studies.

### Data extraction

Data were extracted from included articles via a custom-made form in REDCap [23]. One reviewer (AM) extracted data from all studies, while another member of the review team (DD, AS, MM) performed duplicate data extraction from a randomly selected subsample of 20% of included studies. For each study, data were extracted for study design, including information related to the study population and sample size; methods of measuring both time-use components and health outcomes; covariates; and findings for different reallocations, including any subgroup analyses. The results of reallocations were primarily extracted from tables. However, for studies that did not report results in a tabular form, values were extracted from the text, with additional values extracted from graphs using Plot Digitizer [24]. If multiple models were used, results were extracted from the most adjusted models.

### Risk of bias

The Newcastle-Ottawa scale for observational studies was used to assess risk of bias of the included studies [25]. The risk of bias assessment was completed by one reviewer (AM), with a randomly selected subsample of 20% of included studies assessed in duplicate by another member of the review team (DD, AS, MM). The overall risk of bias scores were classified as “good quality” (7–9 points), “fair quality” (4–6 points), and “poor quality” (0–3 points).

### Data synthesis

Summarising results from CoDA time-use reallocation studies can be challenging due to several factors [26]. Firstly, there are 12 individual one-for-one reallocations possible between the four commonly used time-use components; sleep, SB, LPA, and MVPA (e.g., sleep to MVPA, MVPA to sleep, SB to MVPA). Also, individual studies may report findings for different types (one-for-one, one-for-remaining) and durations of reallocations (e.g., 10 min/day, 20 min/day, 30 min/day). Additionally, the studies may or may not report significance levels or confidence intervals (CIs) for associations, which may hinder the ability to determine whether a specific reallocation is significantly associated with a particular health outcome or not. These differences between studies complicate the task of summarising results consistently across studies.

To simplify the synthesis and presentation of results, studies presenting results of one-for-one reallocations involving sleep, SB, LPA, and MVPA in a 24-hour day or a waking day were first summarised, as previous reviews show most studies have examined these behaviours and reallocation type [26]. This was done using a simple grading system for each health outcome to identify whether a reallocation was significantly and favourably associated with a health outcome (↑), significantly and unfavourably associated with a health outcome (↓), or not significantly associated or mixed associations with a health outcome (↔), as done previously [26, 27]. Only studies reporting significance levels or confidence intervals were included in this synthesis. If a study presented stratified results (e.g., for different sociodemographic groups), the majority of results needed to show the same response in order to be graded favourable or unfavourable, otherwise they were recorded as ↔. Studies that reported results for distinct age groups (e.g., children and adults) were included separately in the synthesis. For the purpose of summarising results, the ActivPAL domains of standing and stepping were classified as LPA and MVPA, respectively, as in previous studies [26].

Results from all the studies reporting one-for-one reallocations for sleep, SB, LPA and MVPA were collated into an online interactive graphical interface. As in previous studies, results were standardised across different outcome variables by dividing the change in outcome by the standard deviation of the outcome. Studies reporting percentage change in an outcome were first converted to an absolute change based on the sample mean. Odds ratios were converted to Cohen’s *d* effect sizes [28] for display purposes. All studies with mortality as an outcome used the same effect size (Hazard Ratios), so no standardisation was necessary.

Findings for less frequently analysed time-use components (e.g., bouted vs. non-bouted time, sedentary screen time vs. sedentary non-screen time) and reallocation types were summarised narratively.

## Results

### Study selection

**Fig. 1:**
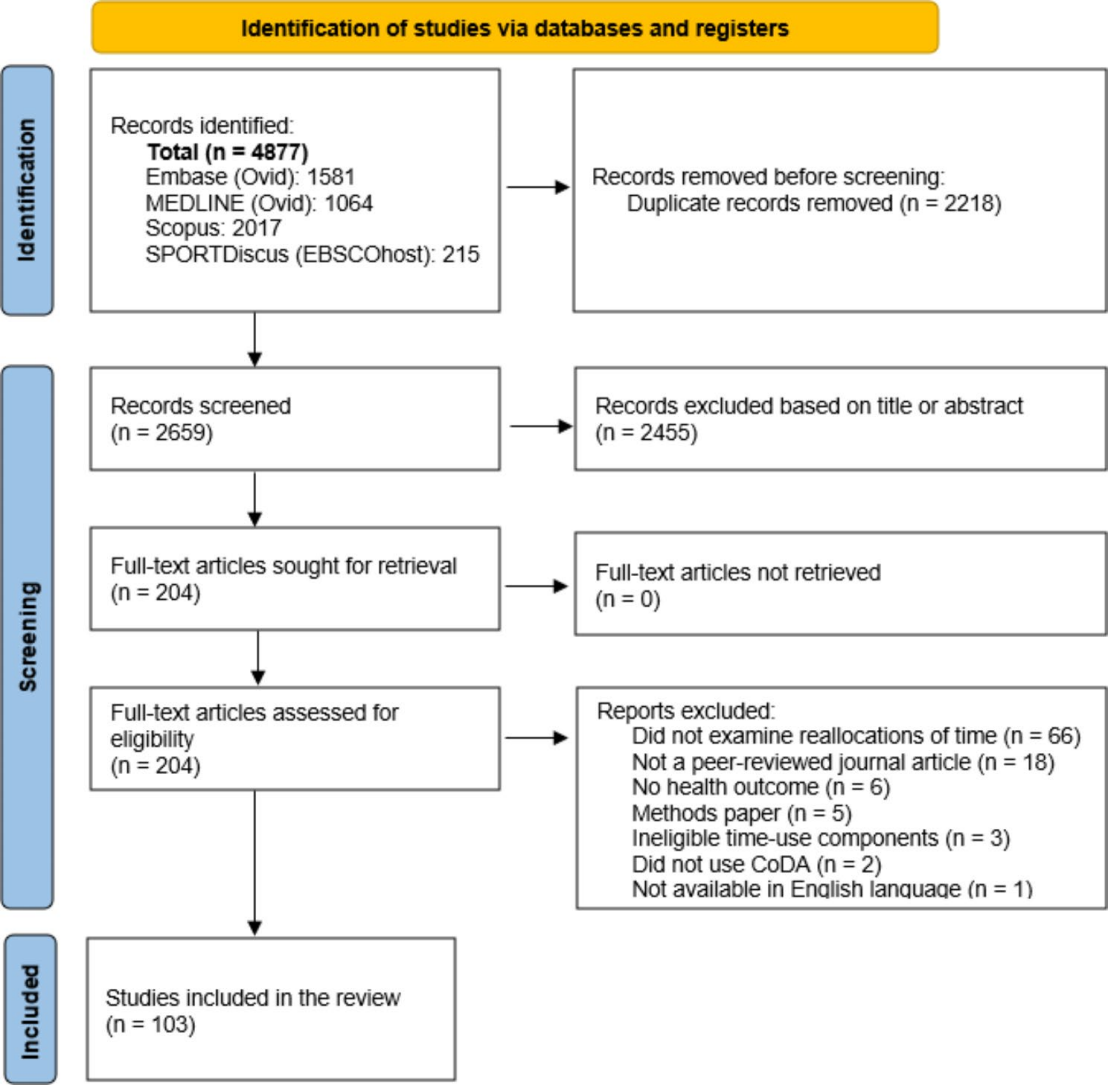
Flow diagram of the search and study selection process. Abbreviations: CoDA, Compositional data analysis

In total, 4877 studies were identified through database searches (Fig. 1). After duplicates were removed, 2659 unique studies remained for screening. After title and abstract screening, 204 studies remained for full-text review. A total of 103 studies [6, 9–12, 14–16, 29–123] met the inclusion criteria and were included in the review. The most common reason for exclusion was not examining reallocations of time. No additional studies were identified through backward citation tracking (I.e., examination of the reference lists of the included studies).

### Study characteristics

Of the included studies, 77 had a cross-sectional design, 21 had a longitudinal design and one was a randomised controlled trial. Four studies presented both cross-sectional and longitudinal results (Additional file 3). The United Kingdom (*n* = 21) was the most represented country out of 28 countries included in the studies. Adult participants (18–64 years) were the most represented age group (*n* = 48), while young children (< 5 years) were the least represented age group (*n* = 10). Adiposity was the most commonly studied health outcome (*n* = 41), followed by mental health (*n* = 26), cardiometabolic biomarkers (*n* = 21), general/perceived health (*n* = 10), fitness (*n* = 10), cognitive health (*n* = 8), pain/injury (*n* = 8), motor skills (*n* = 6), chronic disease/conditions (*n* = 6), mortality (*n* = 5), and academic achievement (*n* = 2). Several studies assessed multiple outcomes; therefore, the total number of analysed outcomes exceeds 103. The sample sizes of included studies ranged from 28 to 130,239, with a median of 574. Many included studies did not report the name of the data source (*n* = 28). However, notably some samples were used in multiple studies, for example the 2005-06 National Health and Nutrition Examination Survey (NHANES) (*n* = 5), the Child Health CheckPoint study (n = 4), the Meiji Yasuda LifeStyle (MYLS) study (*n* = 3), the Physical wOrk DEmands and Prospective register-based Sickness Absence (PODESA) study (*n* = 3) and the Danish Physical ACTivity cohort with Objective measurements (DPHACTO) (*n* = 3) were all used in three or more included studies. Most studies (n = 61) employed 24-hour measurement, with 38 of these studies using device measurement for all behaviours; 16 studies using device measurement for waking behaviours and self-report for sleep; and 8 studies using 24-hour recalls (one study presented results for device- and recall-measured data). Reallocations of time amongst sleep, SB, LPA and MVPA were the most common (*n* = 75), with 16 studies investigating reallocations within sub-compositions of these behaviours (e.g., SB at work and SB in leisure time; PA at weekdays and PA at weekends). Six studies presented results for different time-use domains based on time-use recalls which included some contextual information. For example, some studies analysed reallocations between activities such as screen time, self-care, and passive transport. Four studies investigated the influence of reallocations involving bouted vs. non-bouted activity, with different bout durations explored across the studies.

While most studies explored one-for-one reallocations (*n* = 92), around a quarter of studies reported one-for-remaining reallocations (*n* = 28), and several presented both (*n* = 16). Five studies also presented other types of reallocations.

### Risk of bias

The average score on the Newcastle-Ottawa scale for cross-sectional studies was 5.9, indicating fair quality (Additional file 4). The most common reason for being marked down was in relation to sample size justification. The average score on the Newcastle-Ottawa scale for longitudinal studies was 6.9 (Additional file 5), also indicating fair quality. The most common reason for being marked down was in relation to the adequacy of the follow-up cohort.

### Health outcomes associated with reallocations of time

**Fig. 2:**
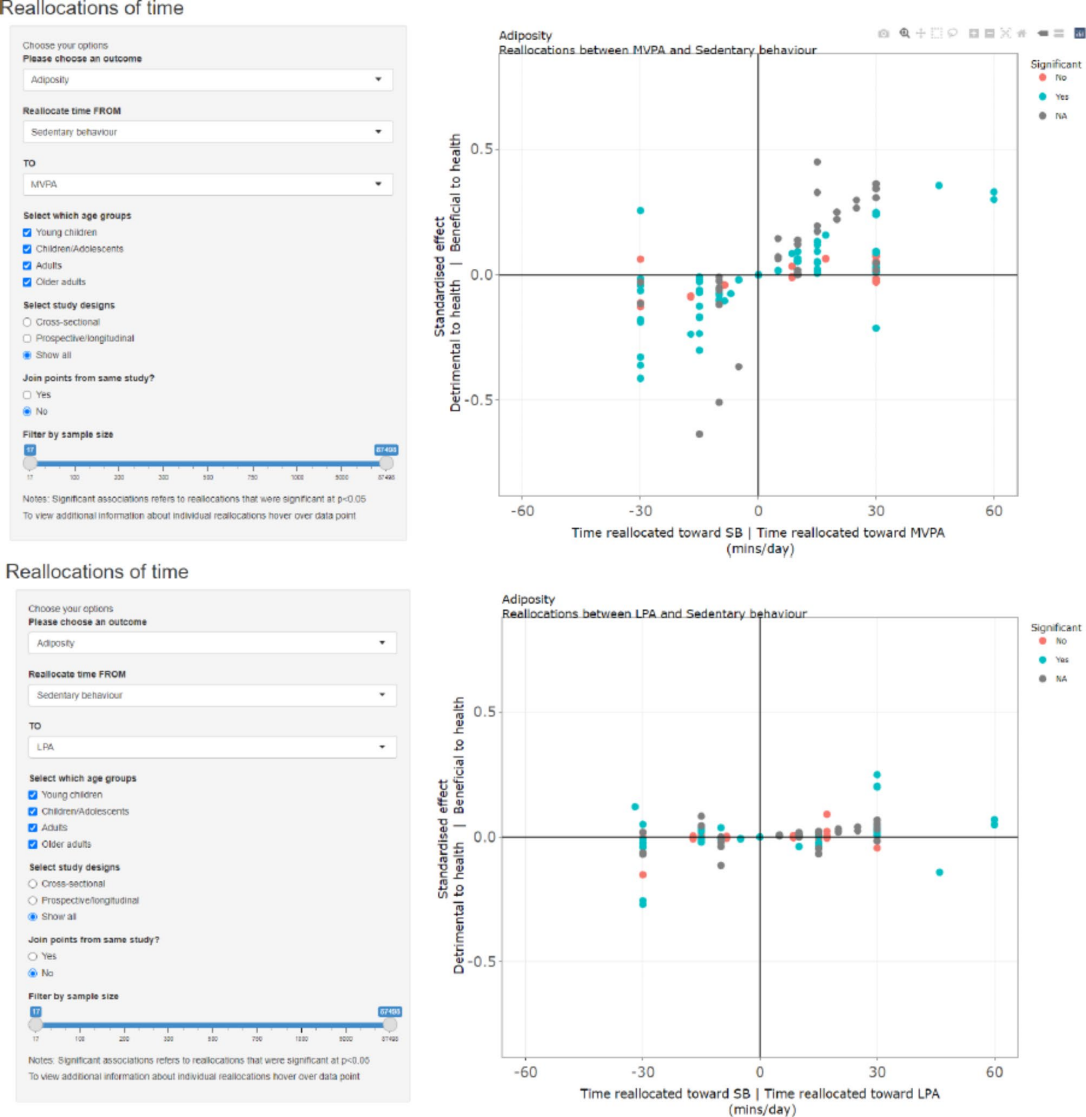
Example displays of online graphical interface

Results from studies exploring one-for-one reallocations exploring sleep, SB, LPA and MVPA are summarised in Tables 1 and 2, Additional file 6 and in an interactive web app [124]. Users can select a pair of behaviours for time-use reallocation and a health outcome of interest. Examples of the display for the analyses of reallocations from SB to MVPA and from SB to LPA in relation to adiposity are shown in Fig. 2. https://xt2oo6-aaron-miatke.shinyapps.io/review/.

**Table 1:** Health outcomes associated with reallocations of time between sleep, SB, LPA and MVPA from cross-sectional studies

Reallocation		Adiposity			Biomarkers			Mental health			Cognitive health			Fitness			Chronic disease			General health			Motor skills			Other		
From	To	↑	↔	↓	↑	↔	↓	↑	↔	↓	↑	↔	↓	↑	↔	↓	↑	↔	↓	↑	↔	↓	↑	↔	↓	↑	↔	↓
	LPA	8	9	7	6	20	1	2	7	0	0	0	3	2	2	4	0	0	0	0	3	0	0	2	2	1	0	1
Sleep	MVPA	18	7	0	14	13	0	8	4	0	1	2	0	4	4	0	0	0	0	3	0	0	3	4	0	0	1	1
	SB	0	17	8	0	26	3	0	10	3	1	1	1	0	3	2	0	0	0	0	3	0	2	4	1	0	1	1
	LPA	15	15	3	9	29	0	4	11	1	0	3	3	3	8	2	0	1	0	0	2	1	0	3	7	3	0	0
SB	MVPA	22	10	1	20	18	0	13	5	0	0	4	0	11	2	0	1	0	0	3	0	0	6	4	0	3	0	0
	Sleep	9	16	0	1	26	1	4	10	0	0	2	1	2	3	0	0	0	0	0	3	0	1	4	2	1	0	1
	MVPA	16	9	3	13	19	0	5	9	0	4	1	0	4	4	0	1	0	0	3	0	0	9	1	0	0	1	1
LPA	SB	3	16	12	0	26	6	1	10	4	2	4	0	2	3	0	0	1	0	1	2	0	7	3	0	0	0	2
	Sleep	7	9	8	1	20	6	1	6	2	2	1	0	3	1	1	0	0	0	0	3	0	2	2	0	1	0	1
	LPA	0	11	16	0	19	13	0	6	8	0	2	3	0	3	5	0	0	1	0	0	3	0	2	8	1	1	0
MVPA	SB	1	8	21	0	15	17	0	5	11	0	4	0	0	0	5	0	0	1	0	0	3	0	4	6	0	0	2
	Sleep	0	5	19	0	12	15	0	3	9	0	2	1	0	0	5	0	0	0	0	0	3	0	4	3	1	0	1

**Table 2:** Health outcomes associated with reallocations of time between sleep, SB, LPA and MVPA from longitudinal studies

Reallocation		Adiposity			Biomarkers			Mental health			Cognitive health			Chronic disease			Mortality		
From	To	↑	↔	↓	↑	↔	↓	↑	↔	↓	↑	↔	↓	↑	↔	↓	↑	↔	↓
	LPA	0	1	0	0	0	0	0	4	0	0	0	0	0	1	0	1	0	0
Sleep	MVPA	0	1	0	0	0	0	1	3	1	0	0	0	1	0	0	1	0	0
	SB	0	1	0	0	0	0	1	3	0	0	0	0	0	0	1	0	1	0
	LPA	2	10	0	2	3	0	1	4	1	0	3	0	1	0	0	1	0	0
SB	MVPA	3	6	0	0	5	0	3	3	0	0	3	0	1	0	0	1	0	0
	Sleep	0	1	0	0	0	0	2	3	1	0	0	0	1	0	0	0	1	0
	MVPA	1	5	0	0	5	0	1	3	0	0	3	0	1	0	0	1	0	0
LPA	SB	0	4	2	0	3	2	0	4	0	0	0	0	0	0	1	0	1	1
	Sleep	0	1	0	0	0	0	0	4	0	0	0	0	0	1	0	0	0	1
	LPA	0	3	2	0	5	0	0	3	1	0	0	0	0	0	1	0	0	1
MVPA	SB	0	2	3	0	5	0	0	3	1	0	0	0	0	0	1	0	0	2
	Sleep	0	0	0	0	0	0	1	3	1	0	0	0	0	0	1	0	0	1

↑, number of reallocations that were favourable for health; ↔, number of reallocations that were not significantly related to health; ↓, number of reallocations that were unfavourable for health. For example, in the first row of the first cell, when considering the association between reallocating time from sleep to LPA and adiposity, 0 studies reported a significant, favourable association; 1 study reported no significant association; 0 studies reported a significant, unfavourable association. Only studies presenting CIs or significance levels were included in the summary. Numbers for corresponding reallocations do not necessarily match due to some studies only reporting outcomes for some reallocations. (*n* = 12 studies contributed to grading)

Abbreviations: SB, sedentary behaviour; LPA, light physical activity; MVPA, moderate-to-vigorous physical activity; CIs, confidence intervals

### Reallocations involving sleep, sedentary behaviour, and physical activity

Across different outcomes and populations, reallocations of time between sleep, SB, LPA and MVPA were similarly associated with health outcomes. Reallocating time to MVPA was generally favourable and reallocating time away from MVPA was generally unfavourable for health, no matter which behaviour was substituted or being used as a substitute (Tables 1 and 2, Additional file 6 and Additional file 7). Health associations for reallocations involving sleep, SB and LPA (but not MVPA) were inconsistent, and they varied depending on the health outcome. However, even in instances where results were relatively consistent (e.g., favourable association of reallocating SB to LPA for adiposity), the magnitude of the associations were generally much smaller than for reallocations involving MVPA.

Reallocations that did not report confidence intervals or significance levels were generally consistent with those that did, both regarding the magnitude and direction of associations, as shown in the interactive web app.

#### Sleep

Reallocating time to sleep from MVPA was unfavourable for most health outcomes. Reallocating time to sleep from either SB or LPA was generally not found to be associated with health. However, there was some evidence that reallocating time toward sleep from LPA was favourable for cognitive health and unfavourable for mortality risk. There was also a trend towards favourable outcomes of reallocating time to sleep from SB (for adiposity, mental health and fitness) and LPA (for motor skills) in cross-sectional studies.

#### Sedentary behaviour

Reallocating time to SB from MVPA was unfavourable for most health outcomes. Reallocating time to SB from sleep was generally not found to be associated with health, with a trend for unfavourable results for adiposity (0↑, 17↔, 8↓, cross-sectional results) and fitness (0↑, 3↔, 2↓, cross-sectional results). Reallocating time to SB from LPA was unfavourable for adiposity, with some evidence of unfavourable associations also displayed for biomarkers using cross-section results (0↑, 26↔, 6↓), but not longitudinal results. The only instances in which reallocating time toward SB generally showed a favourable association with health was for motor skills when time was reallocated from LPA (7↑, 3↔, 0↓, all results cross-sectional), and for cognitive health also when reallocating time from LPA (2↑, 4↔, 0↓, all results cross-sectional).

#### Light physical activity

Reallocating time to LPA from MVPA was unfavourable for most health outcomes. There was some evidence that reallocating time to LPA from SB was favourable for adiposity, chronic disease and mortality risk. Reallocating time to LPA from sleep was beneficial for mortality risk in the only study that reported CIs, although similar effect sizes were found in other studies that did not report CIs. There was also a trend towards favourable associations for biomarkers when reallocating time to LPA from SB using cross-sectional results (9↑, 29↔, 0↓), but not longitudinal results. Reallocating time to LPA from SB generally showed unfavourable associations with motor skills. Associations between reallocations of time towards LPA and most other health outcomes were generally not found to be significant.

#### Moderate-to-vigorous physical activity

When considering cross-sectional results, reallocating time toward MVPA was favourable for most health outcomes, and it displayed similar patterns no matter if the time was reallocated from LPA, SB or sleep. For example, favourable outcomes were almost always observed when reallocating time towards MVPA for adiposity, biomarkers, fitness, general/perceived health, mortality and motor skills no matter where the time came from. For mental health, favourable associations were also observed when reallocating time to MVPA, albeit in several studies these associations were not significant. Cognitive health was the only outcome for which studies generally did not find significant relationships with reallocating time towards MVPA. Contributing to this was the fact that some studies reported stratified results, with reallocations of time toward MVPA beneficial for some, but not most of the strata [94, 121]. When considering longitudinal results, reallocating time toward MVPA appeared favourable for mortality and chronic disease no matter where time was drawn from. Reallocating time from SB to MVPA was favourable for adiposity and mental health, however many of the other significant associations observed using cross-sectional data were not as evident when using longitudinal data.

Some studies also used a more granular intensity spectrum. Studies reporting results for moderate physical activity (MPA) and vigorous physical activity (VPA) separately found that reallocating time towards VPA was particularly beneficial for markers of adiposity and fitness in children using both cross-sectional and longitudinal data [95, 107]. One other study separated higher intensity PA (e.g. stair climbing, running or cycling at a high intensity) from walking and also found that reallocating time towards these behaviours was most strongly associated with improved health [74].

#### Variation among cross-sectional and longitudinal results

Fewer studies investigated associations using longitudinal data, making it harder to draw conclusions based on these results, particularly for health outcomes other than adiposity, mental health, and biomarkers. In general, reallocations of time were less likely to be significantly associated with health in longitudinal studies compared to cross-sectional studies, with reallocations from SB to MVPA most consistently associated with favourable health in longitudinal studies. The non-significant findings for longitudinal reallocations were particularly evident for cardio-metabolic biomarkers where the results of nearly all reallocations were non-significant. However, all longitudinal results for biomarkers were from a single study of pregnant women [109], meaning results may not be reflective of the broader population.

#### Reallocations within subcompositions of the day

For studies involving children (*n* = 3), results for reallocations between domains of SB, LPA, and MVPA largely mirrored those that examined overall durations of these behaviours. For example, reallocating time from all other domains towards MVPA accrued during school or leisure time was generally associated with favourable health outcomes [36, 39, 55](Additional file 8).

For adult populations, the results were mixed. Some studies found that reallocating time towards MVPA was associated with lower systolic blood pressure [66], triglycerides [79], waist circumference and low-density lipoprotein cholesterol [73], regardless of whether this occurred during work or leisure time. For other outcomes, such as high-density lipoprotein cholesterol [79] and cardiorespiratory fitness [77], the reallocation towards MVPA was only favourable if it occurred during leisure time, with some studies even reporting detrimental associations for the reallocation towards MVPA if it occurred during work time (e.g., for systolic blood pressure [73], markers of injury or fatigue like absence from work [64], perceived exertion [72] and need for recovery [111], a combined occupational metric related to fatigue and absenteeism [125]). It should be noted that most studies that reported adverse health outcomes for reallocating work hours toward MVPA shared a study sample predominantly made up of Scandinavian blue-collar workers (Additional file 8)

### Reallocations involving other time-use components

#### Time-use recall domains

Studies reporting reallocations for activity types generally supported those studies that used energy expenditure bands insofar as the strongest favourable associations involved reallocating time to physical activity, and the strongest unfavourable associations involved relocating time away from physical activity, no matter which other domains were involved (Additional file 9).

Some studies that used time-use recalls found associations for screen time to be particularly harmful for health. For example, reallocations to screen time from other low-intensity activities (e.g., hobbies, socialising, education/work) were associated with poorer mental health in adolescents [30, 44] and older adults [98]. One study found that reallocating time away from TV viewing and social media use had stronger favourable associations with mental health compared to when time was drawn from other forms of electronic media such as gaming. However, the magnitude of associations for reallocations involving screen-based behaviours and other activities were generally much smaller than for those involving PA (Additional file 9).

#### Bouted vs. non-bouted activity

Results for the associations of reallocations between bouted and non-bouted activity with health outcomes were inconclusive. Some studies (*n* = 2) reported that reallocating time from longer SB bouts to higher intensity activity was more favourable for adiposity than reallocating the equivalent duration from shorter SB bouts (Additional file 10). However, the strength of these associations was generally consistent with those that used total time in SB.

### Findings by socio-demographic groups

The finding that reallocating time towards MVPA was favourably associated with most health outcomes was relatively consistent across age groups, but it appeared to be weaker in young children than in other age groups (Additional file 6 and Additional file 7). There was some variation in findings for reallocations among other behaviours depending on age. For example, when considering reallocations between SB and LPA, more time in LPA at the expense of SB was favourably associated with adiposity and fitness among adults, but the opposite was found for children [9, 47, 52, 69, 101]. However, the strength of the association was relatively low in both cases. Studies stratifying results by sex generally found similar patterns for males and females.

Two studies found that reallocating time towards MVPA was associated with larger reductions in adiposity for participants with overweight and obesity when compared to their “normal-weight” peers [16, 52]. One study conducted stratified analyses by the risk of type 2 diabetes and found that reallocating time away from sitting to either standing or stepping was more strongly associated with improved cardiometabolic biomarkers in those who were at a high risk of diabetes compared to low-risk participants [37]. One study investigated the moderating role of ethnicity and found that reallocating time from SB to MVPA was associated with a reduction in body weight for Latin participants but not for Caucasian participants [102].

## Discussion

The main findings of this systematic scoping review are that: [1] reallocating time towards MVPA is favourably associated with adiposity, cardiometabolic biomarkers, fitness and mental health, with comparable results generally seen regardless of which other behaviour(s) the time was drawn from; [2] reallocating time away from MVPA is unfavourably associated with most outcomes, including adiposity, biomarkers, mental health, fitness, mortality, chronic disease, general/perceived health, and motor skills; [3] associations of reallocations among sleep, SB and LPA with health outcomes are smaller in magnitude and less consistent than reallocations including MVPA, and they vary depending on the health outcome; [4] there is some evidence of beneficial associations for time reallocations to LPA from SB (with biomarkers, chronic disease and adiposity) and from sleep (with biomarkers and mortality); and [126] reallocating time to SB was either unfavourably or not significantly associated with most health outcomes. These findings were also supported by studies using other ways of classifying time use (e.g., according to domains and bouts of activity). A key finding is also that most available studies were cross-sectional, with less evidence available from longitudinal studies. This means many of the results are based on theoretical reallocations of time, rather than any observed changes in time use. Fewer studies also explored cognitive health, chronic disease, general/perceived health, and mortality outcomes.

The finding that reallocations to MVPA and PA are generally beneficial for health, regardless of where the time was drawn from, agrees with the conclusions from previous reviews using both the CoDA [26, 27] and non-CoDA [13] approach. It is also in accordance with findings from studies using different methodological approaches that have suggested time spent in higher intensity activities have a stronger favourable association with health [127–131]. This review found weaker associations for reallocations involving other behaviours (sleep, SB and LPA). Nonetheless, some evidence suggested reallocating time away from SB to either LPA or sleep may have benefits to health. Reallocating time from SB to LPA has been advocated previously, particularly for those unable or unwilling to engage in MVPA [132]. However, studies in the current review suggested that on a minute-for-minute basis, reallocating time to MVPA was 6–7 times more efficient than reallocating time to other behaviours in eliciting favourable changes in adiposity [114], HRQoL [97], and risk of mortality [43]. However, it is worth noting that any given reallocation to MVPA would constitute a much larger relative change compared to reallocating the same absolute amount of time to any other behaviour. In fact, one study found that when considering reallocations as a proportion of the starting composition rather than an absolute amount of time, increasing sleep time by 10% at the expense of other behaviours collectively was more strongly associated with improved health in young children than increasing MVPA by 10% [116]. Similarly, while the impact of reallocations involving MVPA can look substantial in many of the included studies, it is worth considering that the durations of time modelled sometimes resulted in total MVPA being doubled [82, 120], or sometimes even tripled [47] in the samples included. Given the difficulty often encountered by researchers and public health campaigns in increasing time spent in MVPA in a meaningful and sustained way, it may be unrealistic to expect increases of this magnitude in the real world. A better unit of time may be some metric related to the magnitude of changes typically achieved in interventions.

A key additional insight from CoDA studies compared to non-CoDA isotemporal substitution studies was the non-linearity and asymmetry of contrasting reallocations (as displayed in the interactive web app), which reflects dose-response relationships observed in experimental studies [133, 134]. Associations for reallocations must be considered relative to a reference composition. Nearly all studies included in this review modelled reallocations from the compositional mean of the study sample (or the mean of a particular subgroup). However, one study showed that reallocating time to MVPA was more strongly associated with reduced risk of cardiovascular disease when using a reference composition with a lower level of MVPA [10]. A similar observation was made for risk of mortality in another large study using pooled data from six cohorts [43]. In fact, for more active participants (30 min/day of MVPA), reallocating SB towards LPA was just as beneficial as reallocating SB towards MVPA. Two studies found that children with overweight and obesity saw greater reductions in adiposity for the same reallocations towards MVPA [16, 52] (from either sleep, SB or LPA) than their “normal-weight” peers. Similarly, another study found that those at high risk of developing type 2 diabetes were more likely to see improvements in biomarkers than those with a lower risk when reallocating time away from SB [37] (to either LPA or MVPA). It is unclear what the physiological basis is for these differences, or if it is simply that absolute time reallocations are considered relative to lower MVPA durations in these subgroups. Taken together, these results suggest that the less active and less healthy individuals would likely benefit more from reallocating time towards MVPA, compared with their more active and more healthy peers.

This review identified a selection of studies that did not find beneficial association for reallocations to MVPA – if the reallocations occurred during work time. This finding is consistent with the idea of the “physical activity paradox” which suggests that while PA accrued during leisure has consistent, positive effects on health, PA accumulated during work is not always beneficial to health [135]. These differences may be related to the types and context of physical activities people undertake during work hours (e.g., sustained postures, awkward positions of repetitive manual tasks in a stressful environment) compared to leisure (e.g., walking in nature, playing sports with friends). The type of occupation and the health outcomes considered may also play a role. For example, unfavourable associations were generally observed in samples of blue-collar workers for outcomes related to absenteeism, fatigue, and injury [64, 72, 77]. However, in a sample of office workers, the unfavourable association of reallocating work time toward MVPA was less apparent [79]. It is also unclear how some studies controlled for time spent during the rest of the day (work or leisure) when investigating these domain-specific reallocations of time.

Strengths of our review include its broad scope, including any study population, time-use composition or health outcome. Another feature is the creation of an interactive interface that allows readers to explore results graphically. Most studies used a cross-sectional design, limiting the ability of this review to provide conclusions about causal relationships. Moreover, many of the included studies did not report the results for all possible reallocations (e.g., only reporting the results for reallocations of time from SB to MVPA). It is possible that unreported reallocations were more likely to be non-significant, meaning the importance of some reallocations may have been overstated. Furthermore, many studies did not report CIs or significance levels for the differences in the health outcomes associated with reallocations of time. However, as it can be seen in the interactive web app, the strength and direction of these associations were generally similar to those which did make statistical inferences. While evidence generally suggested that reallocating time between behaviours was associated with health, particularly reallocations involving MVPA, a substantial portion of studies found reallocations not to be significantly associated with health. Some of this variation in findings may be attributable to differences in how time-use components were measured. For example, differences in device placement [136] (hip vs. wrist vs. thigh) and methods of behaviour classification [137] (different cut-points, machine learning methods etc.) can result in vastly different estimates of time spent in each behaviour, which in turn will likely impact the associations of different reallocations. It is also possible that differences in populations, sample sizes and baseline activity compositions of the included studies influenced how likely reallocations were to be significantly associated with health.

Future studies employing the CoDA approach are essential for broadening our understanding of the associations between time reallocations and a diverse range of health outcomes. To date, most research in this area has predominantly focused on adiposity and biomarkers, and mental health, leaving other health outcomes such as cognitive, academic, and social outcomes relatively unexplored. It is crucial to consider that certain sedentary behaviours, such as reading and socialising, may positively influence these outcomes. This is supported by our finding on a possibly favourable association between reallocation of time from LPA to SB with cognitive health.

Young child and adolescent populations were also underrepresented in previous research and should be a focus in the future. More research is also needed to elucidate how longitudinal reallocations are associated with health, given that most previous studies were cross-sectional. Longitudinal studies will also allow for an understanding of how people actually reallocate their time, rather than the commonly analysed “hypothetical” one-for-one substitutions. It is noteworthy that even the longitudinal studies that were included in the current review generally presented reallocations in terms of one-for-one swaps between behaviours. While these types of reallocations may be useful from a messaging perspective [138], they are unlikely to accurately reflect how people make reallocations in the real world. Given the aim of many public health initiatives is to increase physical activity on a population level, studies are needed to elucidate how people practically make reallocations in order to achieve an increase in activity. Moreover, it is worth investigating what factors (e.g., socio-demographic determinants) influence the different reallocation choices that people make. This will allow for more targeted approaches to reduce the burden of physical inactivity. Future studies should also apply CoDA to data from intervention studies to better understand how real (as opposed to hypothetical) reallocations of time are associated with health. Intervention studies will also allow for dose-response relationships to be examined. Most studies currently use energy expenditure bands; however, future research may benefit from additionally considering the context of these behaviours. This may give further understanding of the mechanisms for the differences in our findings for work and leisure MVPA.

The implications of this review are broad. The asymmetry of relationships suggests that, on a minute-for-minute basis, the most relative benefit will be seen when reallocating time toward MVPA among more inactive individuals. It also suggests that even modest increases in MVPA may be quite useful in reducing the health burden of inactivity at the population level. Conversely, reallocating time away from MVPA showed even stronger (negative) associations. Given that activity levels tend to decrease across the lifespan [139, 140], particularly after certain major life events [141], finding how and when people reallocate time away from MVPA and preventing this may be particularly beneficial. The fact that health benefits can still be seen when reallocating time away from SB to LPA, albeit much smaller in magnitude, is promising for populations that may not engage in MVPA, such as some older and clinical populations.

## Conclusion

In conclusion, reallocating time towards MVPA from any behaviour(s) is generally associated with the strongest benefits to health, while reallocating time away from MVPA to any behaviour(s) is associated with the strongest detriment to health. Some beneficial associations were seen when reallocating time from SB to both LPA and sleep; however, the strength of the association was much lower than for any reallocations involving MVPA. Some evidence suggested that researchers should consider information on where, how and when activity occurs when investigating reallocations of time in future studies. Furthermore, most studies investigated outcomes related to adiposity, cardio-metabolic biomarkers, and mental health in adult populations. Future studies may benefit from investigating associations with other health outcomes (e.g., cognitive, social and developmental outcomes), which may be particularly relevant to other populations. More research is also needed using longitudinal data, which will give insight into how people actually reallocate their time under different conditions in real life. To determine the health effects of real (as opposed to hypothetical) reallocations of time, more isotemporal substitution studies should be conducted using data from intervention trials.

### Electronic supplementary material

Below is the link to the electronic supplementary material.

**Supplementary Material 1 Table S1**. Preferred Reporting Items for Systematic reviews and Meta-Analyses extension for Scoping Reviews (PRISMA-ScR) Checklist

**Supplementary Material 2**

**Supplementary Material 3:Table S2**. Characteristics of included studies

**Supplementary Material 4: Table S3**. Newcastle-Ottawa Scale gradings for cross-sectional studies

**Supplementary Material 5: Table S4**. Newcastle-Ottawa Scale gradings for prospective studies

**Supplementary Material 6: Table S5**. Summary of each study contributing to grading

**Supplementary Material 7: Table S6**. Summary of results for studies presenting reallocations between sleep, sedentary behaviour and physical activity

**Supplementary Material 8: Table S7**. Findings for studies reallocating time during subcompositions of the day

**Supplementary Material 9: Table S8**. Results for studies reporting reallocations between recall domains

**Supplementary Material 10: Table S9**. Findings from studies reporting reallocations for bouts of activities

## Data Availability

Not applicable.

## Abbreviations

CIs: Confidence intervals
CoDA: Compositional data analysis
DPHACTO: Danish Physical ACTivity cohort with Objective measurements
LPA: Light physical activity
MPA: Moderate physical activity
MYLS: Meiji Yasuda LifeStyle Study
MVPA: Moderate-to-vigorous physical activity
NHANES: National Health and Nutrition Examination Survey
PA: Physical activity
PCC: Participants, Concept, Context
PODESA: Physical wOrk DEmands and Prospective register-based Sickness Absence study
PRISMA-ScR: Preferred Reporting Items for Systematic Reviews and Meta-Analyses extension for Scoping Reviews
SB: Sedentary behaviour
VPA: Vigorous physical activity

## References

[1] Warburton DE, Bredin SS (2017). Health benefits of physical activity: a systematic review of current systematic reviews. Curr Opin Cardiol.

[2] Chaput J-P, Dutil C, Featherstone R, Ross R, Giangregorio L, Saunders TJ (2020). Sleep duration and health in adults: an overview of systematic reviews. Appl Physiol Nutr Metab.

[3] Saunders TJ, McIsaac T, Douillette K, Gaulton N, Hunter S, Rhodes RE (2020). Sedentary behaviour and health in adults: an overview of systematic reviews. Appl Physiol Nutr Metab.

[4] Fang K, Mu M, Liu K, He Y (2019). Screen time and childhood overweight/obesity: a systematic review and meta-analysis. Child Care Health Dev.

[5] Dumuid D, Pedišić Ž, Stanford TE, Martín-Fernández J-A, Hron K, Maher CA (2019). The compositional isotemporal substitution model: a method for estimating changes in a health outcome for reallocation of time between sleep, physical activity and sedentary behaviour. Stat Methods Med Res.

[6] Chastin SFM, Palarea-Albaladejo J, Dontje ML, Skelton DA. Combined effects of time spent in physical activity, sedentary behaviors and sleep on obesity and cardio-metabolic health markers: a novel compositional data analysis approach. PLoS ONE. 2015;10(10) (no pagination). 10.1371/journal.pone.0139984 PMC4604082 26461112

[7] Dumuid D, Pedišić Ž, Palarea-Albaladejo J, Martín-Fernández JA, Hron K, Olds T (2020). Compositional data analysis in time-use epidemiology: what, why, how. Int J Environ Res Public Health.

[8] Pedišić Ž, Dumuid D, Olds S (2017). Integrating sleep, sedentary behaviour, and physical activity research in the emerging field of time-use epidemiology: definitions, concepts, statistical methods, theoretical framework, and future directions. Kinesiology.

[9] Biddle GJH, Henson J, Biddle SJH, Davies MJ, Khunti K, Rowlands AV (2021). Modelling the reallocation of Time spent sitting into physical activity: Isotemporal Substitution vs. compositional Isotemporal Substitution. Int J Environ Res Public Health.

[10] Yerramalla MS, McGregor DE, van Hees VT, Fayosse A, Dugravot A, Tabak AG (2021). Association of daily composition of physical activity and sedentary behaviour with incidence of cardiovascular disease in older adults. Int.

[11] Clarke AE, Janssen I (2021). A compositional analysis of time spent in sleep, sedentary behaviour and physical activity with all-cause mortality risk. Int.

[12] Dumuid D, Olds T, Wake M, Rasmussen CL, Pedisic Z, Hughes JH et al. Your best day: an interactive app to translate how time reallocations within a 24-hour day are associated with health measures. PLoS ONE. 2022;17(9 September) (no pagination). 10.1371/journal.pone.0272343 PMC9451088 36070284

[13] Grgic J, Dumuid D, Bengoechea EG, Shrestha N, Bauman A, Olds T (2018). Health outcomes associated with reallocations of time between sleep, sedentary behaviour, and physical activity: a systematic scoping review of isotemporal substitution studies. Int J Behav Nutr Phys Activity.

[14] Pelclova J, Stefelova N, Dumuid D, Pedisic Z, Hron K, Gaba A (2020). Are longitudinal reallocations of time between movement behaviours associated with adiposity among elderly women? A compositional isotemporal substitution analysis. Int J Obes (Lond).

[15] Le F, Yap Y, Tung NYC, Bei B, Wiley JF (2022). The Associations between Daily Activities and affect: a compositional Isotemporal Substitution Analysis. Int J Behav Med.

[16] Fairclough SJ, Dumuid D, Taylor S, Curry W, McGrane B, Stratton G (2017). Fitness, fatness and the reallocation of time between children’s daily movement behaviours: an analysis of compositional data. Int.

[17] Migueles JH, Aadland E, Andersen LB, Brønd JC, Chastin SF, Hansen BH (2022). GRANADA consensus on analytical approaches to assess associations with accelerometer-determined physical behaviours (physical activity, sedentary behaviour and sleep) in epidemiological studies. Br J Sports Med.

[18] Miatke A, Dumuid D, Olds T, Maher C, Fraysse F, Mellow M. (2022) The association between reallocation of time and health using compositional data analysis: a scoping review. osf.io/2de9h. 10.1186/s12966-023-01526-x PMC10588100 37858243

[19] Tricco AC, Lillie E, Zarin W, O’Brien KK, Colquhoun H, Levac D (2018). PRISMA extension for scoping reviews (PRISMA-ScR): checklist and explanation. Ann Intern Med.

[20] Peters MD, Godfrey CM, Khalil H, McInerney P, Parker D, Soares CB (2015). Guidance for conducting systematic scoping reviews. JBI Evid Implement.

[21] Merkus SL, Mathiassen SE, Lunde L-K, Koch M, Wærsted M, Forsman M (2021). Can a metric combining arm elevation and trapezius muscle activity predict neck/shoulder pain? A prospective cohort study in construction and healthcare. Int Arch Occup Environ Health.

[22] Gupta N, Bjerregaard SS, Yang L, Forsman M, Rasmussen CL, Rasmussen CDN et al. Does occupational forward bending of the back increase long-term sickness absence risk? A 4-year prospective register-based study using device-measured compositional data analysis. 2022. 10.5271/sjweh.4047 PMC10546616 35894796

[23] Harris PA, Taylor R, Thielke R, Payne J, Gonzalez N, Conde JG (2009). Research electronic data capture (REDCap)—a metadata-driven methodology and workflow process for providing translational research informatics support. J Biomed Inform.

[24] app PDo. Plot Digitizer online app [Available from: https://plotdigitizer.com/app.

[25] Wells GA, Shea B, O’Connell D, Peterson J, Welch V, Losos M et al. The Newcastle-Ottawa Scale (NOS) for assessing the quality of nonrandomised studies in meta-analyses. 2000.

[26] Janssen I, Clarke AE, Carson V, Chaput J-P, Giangregorio LM, Kho ME (2020). A systematic review of compositional data analysis studies examining associations between sleep, sedentary behaviour, and physical activity with health outcomes in adults. Appl Physiol Nutr Metab.

[27] Zahran S, Visser C, Ross-White A, Janssen I (2023). A systematic review of compositional analysis studies examining the associations between sleep, sedentary behaviour, and physical activity with health indicators in early childhood. J Activity Sedentary Sleep Behav.

[28] Sánchez-Meca J, Marín-Martínez F, Chacón-Moscoso S (2003). Effect-size indices for dichotomized outcomes in meta-analysis. Psychol Methods.

[29] Amagasa S, Inoue S, Murayama H, Fujiwara T, Kikuchi H, Fukushima N (2020). Associations of Sedentary and physically-active behaviors with cognitive-function decline in Community-Dwelling older adults: Compositional Data Analysis from the NEIGE Study. J Epidemiol.

[30] Atkin AJ, Dainty JR, Dumuid D, Kontostoli E, Shepstone L, Tyler R (2021). Adolescent time use and mental health: a cross-sectional, compositional analysis in the Millennium Cohort Study. BMJ Open.

[31] Bezerra TA, Clark CCT, Souza Filho AN, Fortes LS, Mota JAPS, Duncan MJ (2021). 24-hour movement behaviour and executive function in preschoolers: a compositional and isotemporal reallocation analysis. Eur J Sport Sci.

[32] Bianchim MS, McNarry MA, Holland A, Cox NS, Dreger J, Barker AR (2022). A compositional analysis of physical activity, sedentary time, and Sleep and Associated Health Outcomes in children and adults with cystic fibrosis. Int J Environ Res Public Health.

[33] Biddle GJH, Edwardson CL, Henson J, Davies MJ, Khunti K, Rowlands AV (2018). Associations of physical behaviours and behavioural reallocations with markers of Metabolic Health: a compositional data analysis. Int J Environ Res Public Health.

[34] Blodgett JM, Mitchell JJ, Stamatakis E, Chastin S, Hamer M (2022). Associations between the composition of daily time spent in physical activity, sedentary behaviour and sleep and risk of depression: compositional data analyses of the 1970 british cohort study. J Affect Disord.

[35] Booker R, Holmes ME, Newton RL, Norris KC, Thorpe RJ, Carnethon MR (2022). Compositional analysis of Movement Behaviors’ Association on high-sensitivity C-Reactive protein: the Jackson Heart Study. Ann Epidemiol.

[36] Bourke M, Vanderloo LM, Irwin JD, Burke SM, Johnson AM, Driediger M et al. Association between childcare movement behaviour compositions with health and development among preschoolers: finding the optimal combinations of physical activities and sedentary time. J Sports Sci. 2022:1–10. 10.1080/02640414.2022.2134969 36227866

[37] Brakenridge CJ, Healy GN, Sethi P, Carver A, Bellettiere J, Salim A (2021). Contrasting compositions of sitting, standing, stepping, and sleeping time: associations with glycaemic outcome by diabetes risk. Int.

[38] Brown DMY, Kwan MYW, King-Dowling S, Cairney J (2021). Cross-sectional Associations between Wake-Time Movement Compositions and Mental Health in Preschool Children with and without motor coordination problems. Front.

[39] Burns RD, Kim Y, Byun W, Brusseau T (2019). Associations of School Day Sedentary Behavior and Physical Activity with Gross Motor Skills: Use of Compositional Data Analysis. J Phys Act Health.

[40] Cabanas-Sanchez V, Esteban-Cornejo I, Garcia-Esquinas E, Ortola R, Ara I, Rodriguez-Gomez I (2021). Cross-sectional and prospective associations of sleep, sedentary and active behaviors with mental health in older people: a compositional data analysis from the Seniors-ENRICA-2 study. Int.

[41] Carson V, Tremblay MS, Chaput JP, Chastin SF (2016). Associations between sleep duration, sedentary time, physical activity, and health indicators among canadian children and youth using compositional analyses. Appl Physiol Nutr Metab.

[42] Chao L, Ma R, Jiang W (2022). Movement behaviours and anxiety symptoms in chinese college students: a compositional data analysis. Front Psychol.

[43] Chastin S, McGregor D, Palarea-Albaladejo J, Diaz KM, Hagstromer M, Hallal PC (2021). Joint association between accelerometry-measured daily combination of time spent in physical activity, sedentary behaviour and sleep and all-cause mortality: a pooled analysis of six prospective cohorts using compositional analysis. BJSM Online.

[44] Chong KH, Dumuid D, Cliff DP, Parrish AM, Okely AD (2022). Changes in 24-Hour domain-specific Movement Behaviors and their Associations with Children’s Psychosocial Health during the transition from primary to secondary school: a compositional data analysis. J Phys Act Health.

[45] Curtis RG, Dumuid D, Olds T, Plotnikoff R, Vandelanotte C, Ryan J (2020). The Association between Time-Use Behaviors and Physical and Mental Well-Being in adults: a compositional Isotemporal Substitution Analysis. J Phys Act Health.

[46] Del Alfonso-Rosa PCB, McGregor RM, Chastin D, Palarea-Albaladejo SF, Del Pozo Cruz J (2020). Sedentary behaviour is associated with depression symptoms: compositional data analysis from a representative sample of 3233 US adults and older adults assessed with accelerometers. J Affect Disord.

[47] Del Pozo-Cruz J, Irazusta J, Rodriguez-Larrad A, Alfonso-Rosa RM, Alvarez-Barbosa F, Raimundo A (2022). Replacing sedentary behavior with physical activity of different intensities: implications for physical function, muscle function, and disability in Octogenarians living in Long-Term Care Facilities. J Phys Act Health.

[48] Domingues SF, da Silva CD, Faria FR, de Sa Souza H, dos Santos Amorim PR. Sleep, sedentary behavior, and physical activity in brazilian adolescents: achievement recommendations and BMI associations through compositional data analysis. PLoS ONE. 2022;17(4 April) (no pagination). 10.1371/journal.pone.0266926 PMC9000056 35404979

[49] Dumuid D, Lewis LK, Olds TS, Maher C, Bondarenko C, Norton L (2018). Relationships between older adults’ use of time and cardio-respiratory fitness, obesity and cardio-metabolic risk: a compositional isotemporal substitution analysis. Maturitas.

[50] Dumuid D, Maher C, Lewis LK, Stanford TE, Martin Fernandez JA, Ratcliffe J (2018). Human development index, children’s health-related quality of life and movement behaviors: a compositional data analysis. Qual Life Res.

[51] Dumuid D, Mellow ML, Olds T, Tregoweth E, Greaves D, Keage H (2022). Does APOE e4 Status Change how 24-Hour time-use composition is Associated with cognitive function? An exploratory analysis among Middle-to-older adults. J Alzheimers Dis.

[52] Dumuid D, Stanford TE, Pedisic Z, Maher C, Lewis LK, Martin-Fernandez JA (2018). Adiposity and the isotemporal substitution of physical activity, sedentary time and sleep among school-aged children: a compositional data analysis approach. BMC Public Health.

[53] Dumuid D, Wake M, Clifford S, Burgner D, Carlin JB, Mensah FK (2019). The Association of the body composition of children with 24-Hour activity composition. J Pediatr.

[54] Estevan I, Clark C, Molina-Garcia J, Menescardi C, Barton V, Queralt A (2022). Longitudinal association of movement behaviour and motor competence in childhood: a structural equation model, compositional, and isotemporal substitution analysis. J Sci Med Sport.

[55] Fairclough SJ, Dumuid D, Mackintosh KA, Stone G, Dagger R, Stratton G (2018). Adiposity, fitness, health-related quality of life and the reallocation of time between children’s school day activity behaviours: a compositional data analysis. Prev Med Rep.

[56] Fairclough SJ, Hurter L, Dumuid D, Gaba A, Rowlands AV, Cruz BDP (2022). The physical Behaviour Intensity Spectrum and Body Mass Index in School-Aged youth: a compositional analysis of Pooled Individual Participant Data. Int J Environ Res Public Health.

[57] Fairclough SJ, Tyler R, Dainty JR, Dumuid D, Richardson C, Shepstone L (2021). Cross-sectional associations between 24-hour activity behaviours and mental health indicators in children and adolescents: a compositional data analysis. J Sports Sci.

[58] Farrahi V, Kangas M, Walmsley R, Niemela M, Kiviniemi A, Puukka K (2021). Compositional Associations of Sleep and Activities within the 24-h cycle with cardiometabolic health markers in adults. Med Sci Sports Exerc.

[59] Gaba A, Dygryn J, Stefelova N, Rubin L, Hron K, Jakubec L (2021). Replacing school and out-of-school sedentary behaviors with physical activity and its associations with adiposity in children and adolescents: a compositional isotemporal substitution analysis. Environ.

[60] Gaba A, Pedisic Z, Stefelova N, Dygryn J, Hron K, Dumuid D (2020). Sedentary behavior patterns and adiposity in children: a study based on compositional data analysis. BMC Pediatr.

[61] Gaba A, Pelclova J, Stefelova N, Pridalova M, Zajac-Gawlak I, Tlucakova L (2021). Prospective study on sedentary behaviour patterns and changes in body composition parameters in older women: a compositional and isotemporal substitution analysis. Clin Nutr.

[62] Germano-Soares AH, Tassitano RM, Farah BQ, Andrade-Lima A, Correia MA, Gaba A (2021). Reallocating Time from sedentary behavior to physical activity in patients with peripheral artery disease: analyzing the Effects on walking Capacity using Compositional Data Analysis. J Phys Act Health.

[63] Giurgiu M, Ebner-Priemer UW, Dumuid D. Compositional insights on the association between physical activity and sedentary behavior on momentary mood in daily life. Psychol Sport Exerc. 2022;58.

[64] Gupta N, Dencker-Larsen S, Lund Rasmussen C, McGregor D, Rasmussen CDN, Thorsen SV (2020). The physical activity paradox revisited: a prospective study on compositional accelerometer data and long-term sickness absence. Int.

[65] Gupta N, Dumuid D, Korshoj M, Jorgensen MB, Sogaard K, Holtermann A (2018). Is Daily Composition of Movement Behaviors related to blood pressure in working adults?. Med Sci Sports Exerc.

[66] Gupta N, Korshoj M, Dumuid D, Coenen P, Allesoe K, Holtermann A (2019). Daily domain-specific time-use composition of physical behaviors and blood pressure. Int.

[67] Gupta N, Rasmussen CL, Hartvigsen J, Mortensen OS, Clays E, Bultmann U (2022). Physical activity advice for Prevention and Rehabilitation of Low Back Pain- same or different? A study on device-measured physical activity and Register-Based sickness absence. J Occup Rehabil.

[68] Hallman DM, Gupta N, Januario LB, Holtermann A (2021). Work-time compositions of physical behaviors and trajectories of sick leave due to musculoskeletal pain. Int J Environ Res Public Health.

[69] Haszard JJ, Meredith-Jones K, Farmer V, Williams S, Galland B, Taylor R (2020). Non-Wear Time and Presentation of compositional 24-Hour time-use analyses influence conclusions about Sleep and Body Mass Index in Children. J Meas Phys Behav.

[70] Healy S, Brewer B, Garcia J, Daly J, Patterson F, Sweat (2021). Sit, Sleep: a compositional analysis of 24-hr Movement Behaviors and Body Mass Index among children with Autism Spectrum Disorder. Autism Res.

[71] Hofman A, Voortman T, Ikram MA, Luik AI (2022). Substitutions of physical activity, sedentary behaviour and sleep: associations with mental health in middle-aged and elderly persons. J Epidemiol Community Health.

[72] Januario LB, Stevens ML, Mathiassen SE, Holtermann A, Karstad K, Hallman DM (2020). Combined Effects of Physical Behavior Compositions and Psychosocial Resources on Perceived Exertion among Eldercare Workers. Ann Work Expo Health.

[73] Johansson MS, Holtermann A, Marott JL, Prescott E, Schnohr P, Korshoj M (2022). The physical activity health paradox and risk factors for cardiovascular disease: a cross-sectional compositional data analysis in the Copenhagen City Heart Study. PLoS ONE.

[74] Johansson MS, Sogaard K, Prescott E, Marott JL, Schnohr P, Holtermann A (2020). Can we walk away from cardiovascular disease risk or do we have to ‘huff and puff’? A cross-sectional compositional accelerometer data analysis among adults and older adults in the Copenhagen City Heart Study. Int.

[75] Kandola A, Del Pozo Cruz B, Hayes JF, Owen N, Dunstan DW, Hallgren M (2022). Impact on adolescent mental health of replacing screen-use with exercise: a prospective cohort study. J Affect Disord.

[76] Kandola AA, Del Pozo Cruz B, Osborn DPJ, Stubbs B, Choi KW, Hayes JF (2021). Impact of replacing sedentary behaviour with other movement behaviours on depression and anxiety symptoms: a prospective cohort study in the UK Biobank. BMC Med.

[77] Ketels M, Rasmussen CL, Korshoj M, Gupta N, De Bacquer D, Holtermann A (2020). The relation between Domain-Specific Physical Behaviour and Cardiorespiratory Fitness: a cross-sectional compositional data analysis on the physical activity Health Paradox using accelerometer-assessed data. Int J Environ Res Public Health.

[78] Kim Y, Burns RD, Lee DC, Welk GJ (2021). Associations of movement behaviors and body mass index: comparison between a report-based and monitor-based method using Compositional Data Analysis. Int J Obes (Lond).

[79] Kitano N, Kai Y, Jindo T, Fujii Y, Tsunoda K, Arao T (2022). Association of domain-specific physical activity and sedentary behavior with cardiometabolic health among office workers. Scand J Med Sci Sports.

[80] Kitano N, Kai Y, Jindo T, Tsunoda K, Arao T (2020). Compositional data analysis of 24-hour movement behaviors and mental health in workers. Prev Med Rep.

[81] Kuzik N, Naylor PJ, Spence JC, Carson V (2020). Movement behaviours and physical, cognitive, and social-emotional development in preschool-aged children: cross-sectional associations using compositional analyses. PLoS ONE.

[82] Larisch LM, Kallings LV, Hagstromer M, Desai M, Rosen PV, Blom V (2020). Associations between 24 h movement behavior and mental health in office workers. Int J Environ Res Public Health.

[83] Lee J, Walker ME, Gabriel KP, Vasan RS, Xanthakis V (2020). Associations of accelerometer-measured physical activity and sedentary time with chronic kidney disease: the Framingham Heart Study. PLoS ONE.

[84] Lee J, Walker ME, Matthews KA, Kuller LH, Ranjit N, Gabriel KP (2020). Associations of physical activity and sleep with cardiometabolic risk in older women. Prev Med Rep.

[85] Lemos L, Clark C, Brand C, Pessoa ML, Gaya A, Mota J (2021). 24-hour movement behaviors and fitness in preschoolers: a compositional and isotemporal reallocation analysis. Scand J Med Sci Sports.

[86] Lewthwaite H, Olds T, Williams MT, Effing TW, Dumuid D (2019). Use of time in chronic obstructive pulmonary disease: longitudinal associations with symptoms and quality of life using a compositional analysis approach. PLoS ONE.

[87] Ma T, Xie YJ, Bennett T, Lee CD (2022). Time-of-day moderate-to-vigorous physical activity and all-cause mortality in individuals with type 2 diabetes. J Sports Sci.

[88] Machida M, Takamiya T, Amagasa S, Murayama H, Fujiwara T, Odagiri Y (2021). Objectively measured intensity-specific physical activity and hippocampal volume among community-dwelling older adults. J Epidemiol.

[89] Marshall ZA, Mackintosh KA, Gregory JW, McNarry MA (2022). Using compositional analysis to explore the relationship between physical activity and cardiovascular health in children and adolescents with and without type 1 diabetes. Pediatr Diabetes.

[90] Marshall ZA, Mackintosh KA, McNarry MA. Investigating the influence of physical activity composition on arterial stiffness in youth. EJSS (Champaign). 2022:1–8. 10.1080/17461391.2022.2039304 35135413

[91] Martins CML, Clark CCT, Tassitano RM, Filho ANS, Gaya AR, Duncan MJ (2021). School-Time Movement Behaviors and Fundamental Movement Skills in Preschoolers: an Isotemporal reallocation analysis. Percept Mot Skills.

[92] Matricciani L, Dumuid D, Paquet C, Fraysse F, Wang Y, Baur LA (2021). Sleep and cardiometabolic health in children and adults: examining sleep as a component of the 24-h day. Sleep Med.

[93] McGregor DE, Palarea-Albaladejo J, Dall PM, Del Pozo Cruz B, Chastin SFM (2021). Compositional analysis of the association between mortality and 24-hour movement behaviour from NHANES. Eur J Prev Cardiolog.

[94] Migueles JH, Cadenas-Sanchez C, Esteban-Cornejo I, Torres-Lopez LV, Aadland E, Chastin SF (2020). Associations of objectively-assessed physical activity and sedentary time with hippocampal Gray Matter volume in children with Overweight/Obesity. J.

[95] Migueles JH, Delisle Nystrom C, Leppanen MH, Henriksson P, Lof M (2022). Revisiting the cross-sectional and prospective association of physical activity with body composition and physical fitness in preschoolers: a compositional data approach. Pediatr Obes.

[96] Mota JG, Clark CCT, Bezerra TA, Lemos L, Reuter CP, Mota J (2020). Twenty-four-hour movement behaviours and fundamental movement skills in preschool children: a compositional and isotemporal substitution analysis. J Sports Sci.

[97] Ng E, Wake M, Olds T, Lycett K, Edwards B, Le H (2021). Equivalence curves for healthy lifestyle choices. Pediatrics.

[98] Olds T, Burton NW, Sprod J, Maher C, Ferrar K, Brown WJ (2018). One day you’ll wake up and won’t have to go to work: the impact of changes in time use on mental health following retirement. PLoS ONE.

[99] Oviedo-Caro MA, Bueno-Antequera J, Munguia-Izquierdo D (2020). Associations of 24-hours activity composition with adiposity and cardiorespiratory fitness: the PregnActive project. Scand J Med Sci Sports.

[100] Pelclova J, Stefelova N, Hodonska J, Dygryn J, Gaba A, Zajac-Gawlak I (2018). Reallocating time from sedentary behavior to light and moderate-to-vigorous physical activity: what has a stronger association with adiposity in older Adult Women?. Int J Environ Res Public Health.

[101] Powell C, Browne LD, Carson BP, Dowd KP, Perry IJ, Kearney PM (2020). Use of Compositional Data Analysis to show estimated changes in Cardiometabolic Health by reallocating time to light-intensity physical activity in older adults. Sports Med.

[102] Rees-Punia E, Guinter MA, Gapstur SM, Wang Y, Patel AV (2021). Composition of time in movement behaviors and weight change in Latinx, Black and white participants. PLoS ONE.

[103] Ren Z, Tan J, Huang B, Cheng J, Huang Y, Xu P (2022). Association between 24-Hour Movement Behaviors and Smartphone Addiction among Adolescents in Foshan City, Southern China: Compositional Data Analysis. Int J Environ Res Public Health.

[104] Roscoe CMP, Duncan MJ, Clark CCT (2021). The 24-h Movement Compositions in Weekday, Weekend Day or Four-Day Periods differentially associate with Fundamental Movement Skills. Child (Basel).

[105] Rosen P, Dohrn IM, Hagströmer M (2020). Association between physical activity and all-cause mortality: a 15‐year follow‐up using a compositional data analysis. Scand J Med Sci Sports.

[106] Rossen J, Von Rosen P, Johansson UB, Brismar K, Hagstromer M (2020). Associations of physical activity and sedentary behavior with cardiometabolic biomarkers in prediabetes and type 2 diabetes: a compositional data analysis. Phys Sportsmed.

[107] Rubin L, Gaba A, Pelclova J, Stefelova N, Jakubec L, Dygryn J (2022). Changes in sedentary behavior patterns during the transition from childhood to adolescence and their association with adiposity: a prospective study based on compositional data analysis. Arch.

[108] Sampasa-Kanyinga H, Colman I, Dumuid D, Janssen I, Goldfield GS, Wang JL (2021). Longitudinal association between movement behaviours and depressive symptoms among adolescents using compositional data analysis. PLoS ONE.

[109] Sandborg J, Migueles JH, Soderstrom E, Blomberg M, Henriksson P, Lof M (2022). Physical activity, body composition, and Cardiometabolic Health during pregnancy: a Compositional Data Approach. Med Sci Sports Exerc.

[110] Smith E, Fazeli F, Wilkinson K, Clark CCT (2021). Physical behaviors and fundamental movement skills in british and iranian children: an isotemporal substitution analysis. Scand J Med Sci Sports.

[111] Stevens ML, Crowley P, Rasmussen CL, Hallman DM, Mortensen OS, Nygard CH (2020). Accelerometer-measured physical activity at work and need for recovery: a compositional analysis of cross-sectional data. Ann Work Expo Health.

[112] Su J, Wei E, Clark C, Liang K, Sun X (2022). Physical exercise, sedentary behaviour, sleep and depression symptoms in chinese young adults during the COVID-19 pandemic: a compositional Isotemporal Analysis. Int J Mental Health Promotion.

[113] Swindell N, Rees P, Fogelholm M, Drummen M, MacDonald I, Martinez JA (2020). Compositional analysis of the associations between 24-h movement behaviours and cardio-metabolic risk factors in overweight and obese adults with pre-diabetes from the PREVIEW study: cross-sectional baseline analysis. Int.

[114] Talarico R, Janssen I (2018). Compositional associations of time spent in sleep, sedentary behavior and physical activity with obesity measures in children. Int J Obes (Lond).

[115] Taylor RW, Haszard JJ, Farmer VL, Richards R, Te Morenga L, Meredith-Jones K (2020). Do differences in compositional time use explain ethnic variation in the prevalence of obesity in children? Analyses using 24-hour accelerometry. Int J Obes (Lond).

[116] Taylor RW, Haszard JJ, Meredith-Jones KA, Galland BC, Heath AM, Lawrence J (2018). 24-h movement behaviors from infancy to preschool: cross-sectional and longitudinal relationships with body composition and bone health. Int.

[117] Tsunoda K, Kitano N, Kai Y, Jindo T, Uchida K, Arao T (2021). Dose-response relationships of accelerometer-measured sedentary behaviour and physical activity with non-alcoholic fatty liver disease. Aliment Pharmacol Ther.

[118] Verhoog S, Braun KVE, Bano A, van Rooij FJA, Franco OH, Koolhaas CM (2020). Associations of Activity and Sleep with Quality of Life: a compositional data analysis. Am J Prev Med.

[119] Verswijveren S, Lamb KE, Martin-Fernandez JA, Winkler E, Leech RM, Timperio A (2022). Using compositional data analysis to explore accumulation of sedentary behavior, physical activity and youth health. J.

[120] Walmsley R, Chan S, Smith-Byrne K, Ramakrishnan R, Woodward M, Rahimi K (2021). Reallocation of time between device-measured movement behaviours and risk of incident cardiovascular disease. BJSM Online.

[121] Whitaker KM, Zhang D, Gabriel KP, Ahrens M, Sternfeld B, Sidney S (2021). Longitudinal associations of midlife accelerometer determined sedentary behavior and physical activity with cognitive function: the cardia study. J Am Heart Association.

[122] Winkler EAH, Chastin S, Eakin EG, Owen N, Lamontagne AD, Moodie M (2018). Cardiometabolic impact of changing sitting, standing, and stepping in the Workplace. Med Sci Sports Exerc.

[123] Zhang T, Li H, Li C, Zhang L, Zhang Z (2022). The compositional impacts of 2 distinct 24-Hour Movement Behavior change patterns on physical fitness in chinese adolescents. J Phys Act Health.

[124] Miatke A. Reallocations of time 2023 [Available from: https://xt2oo6-aaron-miatke.shinyapps.io/review/.

[125] Van Veldhoven M, Broersen S (2003). Measurement quality and validity of the need for recovery scale. Occup Environ Med.

[126] Mekary RA, Willett WC, Hu FB, Ding EL (2009). Isotemporal substitution paradigm for physical activity epidemiology and weight change. Am J Epidemiol.

[127] Aadland E, Kvalheim OM, Anderssen SA, Resaland GK, Andersen LB (2018). The multivariate physical activity signature associated with metabolic health in children. Int J Behav Nutr Phys Activity.

[128] Costigan SA, Eather N, Plotnikoff R, Taaffe DR, Lubans DR (2015). High-intensity interval training for improving health-related fitness in adolescents: a systematic review and meta-analysis. Br J Sports Med.

[129] Howard B, Winkler E, Sethi P, Carson V, Ridgers ND, Salmon J (2015). Associations of low-and high-intensity light activity with cardiometabolic biomarkers. Med Sci Sports Exerc.

[130] Augustin NH, Mattocks C, Faraway JJ, Greven S, Ness AR (2017). Modelling a response as a function of high-frequency count data: the association between physical activity and fat mass. Stat Methods Med Res.

[131] Samitz G, Egger M, Zwahlen M (2011). Domains of physical activity and all-cause mortality: systematic review and dose–response meta-analysis of cohort studies. Int J Epidemiol.

[132] Chastin SF, De Craemer M, De Cocker K, Powell L, Van Cauwenberg J, Dall P (2019). How does light-intensity physical activity associate with adult cardiometabolic health and mortality? Systematic review with meta-analysis of experimental and observational studies. Br J Sports Med.

[133] Chen Z, Zhu L (2023). Dose–response relationship between physical activity and cardiometabolic risk in obese children and adolescents: a pre-post quasi-experimental study. Front Physiol.

[134] Gallardo-Gómez D, del Pozo-Cruz J, Noetel M, Álvarez-Barbosa F, Alfonso-Rosa RM, del Pozo Cruz B (2022). Optimal dose and type of exercise to improve cognitive function in older adults: a systematic review and bayesian model-based network meta-analysis of RCTs. Ageing Res Rev.

[135] Holtermann A, Krause N, Van Der Beek AJ, Straker L. The physical activity paradox: six reasons why occupational physical activity (OPA) does not confer the cardiovascular health benefits that leisure time physical activity does. BMJ Publishing Group Ltd and British Association of Sport and Exercise Medicine; 2017. 10.1136/bjsports-2017-097965 28798040

[136] Marcotte RT, Petrucci GJ, Cox MF, Freedson PS, Staudenmayer JW, Sirard JR (2020). Estimating sedentary time from a hip-and wrist-worn accelerometer. Med Sci Sports Exerc.

[137] Ahmadi MN, Pavey TG, Trost SG (2020). Machine learning models for classifying physical activity in free-living preschool children. Sensors.

[138] O’Hara BJ, Grunseit A, Phongsavan P, Bellew W, Briggs M, Bauman AE (2016). Impact of the swap it, don’t stop it australian national mass media campaign on promoting small changes to lifestyle behaviors. J Health Communication.

[139] Farooq A, Martin A, Janssen X, Wilson MG, Gibson AM, Hughes A (2020). Longitudinal changes in moderate-to‐vigorous‐intensity physical activity in children and adolescents: a systematic review and meta‐analysis. Obes Rev.

[140] Ortega FB, Konstabel K, Pasquali E, Ruiz JR, Hurtig-Wennlöf A, Mäestu J (2013). Objectively measured physical activity and sedentary time during childhood, adolescence and young adulthood: a cohort study. PLoS ONE.

[141] Gropper H, John JM, Sudeck G, Thiel A (2020). The impact of life events and transitions on physical activity: a scoping review. PLoS ONE.

