# Supplementary material for: The association between reallocations of time and health using compositional data analysis: a systematic scoping review with an interactive data exploration interface
Source: Int J Behav Nutr Phys Act. 2023 Oct 19;20:127. doi: 10.1186/s12966-023-01526-x (PMC10588100; doi:10.1186/s12966-023-01526-x)
Supplement: Supplementary file 2 — Supplementary Material 2 [file 12966_2023_1526_MOESM2_ESM.docx]

## MEDLINE search strategy

Exercise/

Walking/

Running/

Exercise Movement Techniques/

Leisure Activities/

Physical Exertion/

Motor Activity/

Sports/

(exercis* or walk* or running or run or jogging or "leisure activit*" or "resistance training" or (activ* adj2 transport) or (time adj2 behavi*) or (physical* adj2 behavi*) or (physical* adj2 activ*) or mvpa or (play adj2 behav*)).ti,ab,kf.

Sedentary Behavior/

Screen Time/

Reading/

(sedentar* or sitting or reading or screen time or (physical* adj2 inactiv*)).ti,ab,kf.

Sleep/

sleep.ti,ab,kf.

exp Accelerometry/

("movement behav*" or "time use" or "time budget*" or "time spent" or 24h or "24 h" or acceleromet* or actigraph*).ti,ab,kf.

or/1-17

((composition* adj2 data) or (composition* adj2 analys*) or "log ratio*" or ilr or coda or (composition* adj3 substitution) or (composition* adj3 isotemporal) or (composition* adj3 "iso-temporal") or (composition* adj3 reallocation) or (composition* adj3 "re-allocation") or (behavio* adj2 composition*) or (activit* adj2 composition*) or (time adj3 composition*) or (movement adj2 composition*)).ti,ab,kf.

18 and 19

limit 20 to yr="2015 -Current"
