# Supplementary material for: The association between reallocations of time and health using compositional data analysis: a systematic scoping review with an interactive data exploration interface
Source: Int J Behav Nutr Phys Act. 2023 Oct 19;20:127. doi: 10.1186/s12966-023-01526-x (PMC10588100; doi:10.1186/s12966-023-01526-x)
Supplement: Supplementary file 3 — Supplementary Material 3:Table S2. Characteristics of included studies [file 12966_2023_1526_MOESM3_ESM.docx]

Table S2. Characteristics of included studies

| **Study** | **Age range and sample size** | **Study sample** | **Time-use components** | **Measurement device and duration** | **Health outcome(s)** |
| --- | --- | --- | --- | --- | --- |
| Amagasa (2020) | Older adults (n = 511) | Japanese community dwelling older adults (Neuron to Environmental Impact across Generations (NEIGE) study) | SB, LPA, MVPA | Active style Pro HJA-750C (4-7 days) | Cognitive health |
| Atkin (2021) | Adolescents (n = 3485) | Nationally representative UK birth cohort (Millennium Cohort Study wave 6) | Sleep, PA, School-related, Hobbies & socialising, Electronic media, Domestic, personal care, work-related | Recall -not stated (1 weekday & 1 weekend day) | Mental health |
| Bezerra (2020) | Young children (n = 123) | Brazilian preschool children | Sleep, SB, LPA, MVPA | Actigraph WGT3-X, Sleep via parental report (3-6 days) | Cognitive health |
| Bianchim (2022) | Children, Adults (n = 129) | Children/Adults with Cystic Fibrosis | Sleep, SB, LPA, MVPA | ActiGraph GT9X Link, GENEActiv (7 days) | Other |
| Biddle (2018) | Older adults (n = 435) | UK adults at high risk of type 2 diabetes (Walking Away from Diabetes) | Sleep, Sitting, Standing, Stepping | activPAL3 (4-7 days) | Biomarkers |
| Biddle (2021) | Adults, Older adults (n = 1524) | Pooled sample of UK adults/older adults at high risk of developing T2DM. (PROPELS, Walking Away from Diabetes, and Project STAND | Sleep, Sitting, Standing, Stepping | activPAL3 (4-10 days) | Adiposity, Biomarkers |
| Blodgett (2022) | Adults (n = 4738) | UK birth cohort, aged 46yo (1970 British Cohort Study) | Sleep, SB, LPA, MVPA | activPAL3 (7 days) | Mental health |
| Booker (2022) | Adults (n = 2805) | African American adults (Jackson heart study third wave) | Sleep, SB, PA | JHS Physical Activity Cohort (JPAC) survey; JHS Sleep History Form; SB remaining time | Biomarkers |
| Bourke (2022) | Young children (n = 392) | Canadian preschool children (Supporting Physical Activity in the Childcare Environment (SPACE)) | Preschool SB, Preschool LPA, Preschool MVPA | Actical (2-5 days) | Adiposity, General/perceived health, Mental health |
| Brakenridge (2021) | Adults (n = 648) | The Australian Diabetes, Obesity and Lifestyle Study (AusDiab) | Sleep, Sitting, Standing, Stepping | activPAL3 (7 days) | Biomarkers |
| Brown (2021) | Young children (n = 589) | Canadian preschool children (typically developing & at risk of developmental coordination disorder) (Coordination and Activity Tracking in CHildren (CATCH) study) | SB, LPA, MVPA | Actigraph GT3X+ (3-7 days) | Mental health |
| Burns (2019) | Children (n = 409) | US school children | School SB, School LPA, School MVPA | ActiGraph wGT3X-BT (3-5 days) | General/perceived health |
| Cabanas-Sanchez (2021) | Older adults (n = 2489) | Spanish community-dwelling older adults aged ≥ 65 years (Seniors- ENRICA-2) | Sleep, SB, LPA, MVPA | ActiGraph GT9X (4-7 days) | Mental health |
| Carson (2016) | Children, Adolescents (n = 4169) | Nationally representative sample of Canadian children/adolescents (Canadian Health Measures Survey) | Sleep, SB, LPA, MVPA | Actical, sleep self-report (adolescents) or parental report (children) (7 days) | Adiposity, Mental health, Fitness, Biomarkers |
| Chao (2022) | Adults (n = 1475) | Chinese college students (during COVID-19 pandemic) | Sleep, SB, LPA, MVPA | SB and PA, IPAQ; sleep Pittsburgh Sleep Quality Index | Mental health |
| Chastin (2021) | Adults, Older adults (n = 130239) | Pooled international sample 6 cohorts (Pooled sample (Women's Health Study, Whitehall 2, REGARDS, ABC, UK Biobank, 05/06 NHANES)) | Sleep, SB, LPA, MVPA | multiple (7 days) | Mortality |
| Chastin (2015) | Adults (n = 1937) | US adults (2005-06 NHANES) | Sleep, SB, LPA, MVPA | Actigraph 7164, sleep via self-report (7 days) | Adiposity, Biomarkers |
| Chong (2022) | Children, Adolescents (n = 909) | Representative sample of Australian children/adolescents (Longitudinal Study of Australian Children (LSAC) wave 4 & 5) | Sleep, PA, Self-care/domestic, Social, Education, Recreational screen use, Quiet time, Passive transport | Paper based 24-h recall (1 day at each timepoint) | Mental health |
| Clarke (2021) | Adults (n = 2838) | Representative sample of US adults (2005-2006 NHANES) | Sleep, SB, LPA, MVPA | Actigraph PAM-7164. Sleep estimated based on CATI for usual sleep duration to nearest whole hour (4-7 days) | Mortality |
| Curtis (2020) | Adults (n = 430) | Inactive Australian adults (Active Team) | Sleep, SB, LPA, MVPA | GENEActiv (4-7 days) | Adiposity, General/perceived health |
| del Pozo Cruz (2020) | Adults (n = 3233) | Representative sample of US adults (2005-2006 NHANES) | Sleep, SB, LPA, MVPA | ActiGraph AM-7164, sleep self-report (7) | Mental health |
| del Pozo-Cruz (2022) | Older adults (n = 345) | Spanish older adults (>80yo) in long-term care | SB, LPA, MVPA | ActiGraph wGT3X-BT (4-7 days) | Fitness, Other |
| Domingues (2022) | Adolescents (n = 185) | Brazilian adolescents from single school | Sleep, SB, LPA, MVPA | Actigraph GT3X (3-8 days) | Adiposity |
| Dumuid (2018) | Older adults (n = 122) | Community dwelling Australian older adults | Sleep, SB, LPA, MVPA | ActiGraph GT3X+ (4-7 days) | Adiposity, Fitness |
| Dumuid (2018) | Children (n = 5855) | Multi-nation, representative sample of 9-11yo children (International Study of Childhood Obesity, Lifestyle and the Environment (ISCOLE)) | Sleep, SB, LPA, MVPA | Actigraph GT3X+ (4-7 days) | General/perceived health |
| Dumuid (2022) | Older adults (n = 82) | Australian older adults | Sleep, SB, LPA, MVPA | GENEActiv (4-7 days) | Cognitive health |
| Dumuid (2022) | Children (n = 1685) | Representative sample of Australian children (Child Health CheckPoint) | Sleep, Screen time, PA,Quiet time, Passive transport, School-related activity, Domestic/self-care | MARCA (3) | Adiposity, Mental health, Academic achievement |
| Dumuid (2018) | Children (n = 1728) | International sample of urban/suburban children (ISCOLE) | Sleep, SB, LPA, MVPA | Actigraph GT3X+ (4-7 days) | Adiposity |
| Dumuid (2018) | Children (n = 971) | Representative birth cohort of Australian children (Child Health CheckPoint) | Sleep, SB, LPA, MVPA | GENEActiv (8) | Adiposity |
| Estevan (2022) | Children (n = 124) | Spanish school children | SB, LPA, MVPA | Actigraph GT3X+ (4-6 days) | Other |
| Fairclough (2018) | Children (n = 243) | Schoolchildren from Northwest England (Children's Health, Activity, Nutrition: Get Educated! (CHANGE!) intervention) | SB, LPA, MVPA, School SB, School LPA, School MVPA | ActiGraph GT1M (3-7 days) | Adiposity, General/perceived health, Mental health, Fitness |
| Fairclough (2017) | Children (n = 169) | UK primary school students in low income town (Active Schools: Skelmersdale study) | Sleep, SB, LPA, MVPA | ActiGraph GT9X (7 days) | Adiposity, Fitness |
| Fariclough (2022) | Children (n = 1453) | Pooled sample of UK school children (pooled studies) | Acceleration bands: 0-50mg, 50-100mg, 100-150mg, 150-200mg, 200-250mg, 250-300mg, 300-350mg, 350-700mg, >700mg | ActiGraph GT9X, Axivity AX3, GENEActiv (up to 7 days) | Adiposity |
| Fairclough (2021) | Children, Adolescents (n = 359) | Healthy English school children | Sleep, SB, LPA, MVPA | ActiGraph GT9X (3-7 days) | Mental health, Cognitive health |
| Farrahi (2021) | Adults (n = 3443) | Finnish adults from birth cohort (Northern Finland Birth Cohort 1966 study) | Sleep, SB, LPA, MVPA | Hookie AM20; sleep via question asking usual sleep duration (4-14 days) | Adiposity, Biomarkers |
| Gaba (2021) | Children, Adolescents (n = 336) | Czech primary and secondary school children/adolescents | Sleep, School SB, Out-of-school SB, School LPA, Out-of-school LPA, School MPA, Out-of-school MPA, School VPA, Out-of-school VPA | ActiGraph GT9X Link or wGT3X-BT (7) | Adiposity |
| Gaba (2020) | Children (n = 425) | Healthy Czech school-children | SB, LPA, MVPA, SB short bouts, SB middle bouts, SB long bouts | ActiGraph GT3X (4-7 days) | Adiposity |
| Gaba (2021) | Older adults (n = 182) | Healthy Eastern European older women | SB, LPA, MVPA, SB short bouts, SB middle bouts, SB long bouts | ActiGraph GT1M (7 days) | Adiposity |
| Germano-Soares (2021) | Older adults (n = 178) | Brazilian older adults with symptomatic peripheral artery disease | SB, LPA, MVPA | ActiGraph GT3X (4-7 days) | Fitness |
| Giurgiu (2022) | Adults (n = 98) | German university students | SB, LPA, MVPA | Move 4 accelerometer (5 days) | Mental health |
| Gupta (2020) | Adults (n = 929) | Danish predominately blue collar workers (Physical wOrk DEmands and Prospective register-based Sickness Absence (PODESA) study. | Sleep, Work SB, Work Standing, Work LPA, Work MVPA, Leisure SB, Leisure Standing, Leisure LPA, Leisure MVPA | ActiGraph GT3X+ (1-5 days) | Pain/injury/illness |
| Gupta (2018) | Adults (n = 827) | Danish predominately blue collar workers (Physical wOrk DEmands and Prospective register-based Sickness Absence (PODESA) study. | SB, LPA, MVPA, Time in bed | Actigraph GT3X+, time in bed self-report (1-4 days) | Biomarkers |
| Gupta (2019) | Adults (n = 669) | Danish predominantly blue collar workers (Danish PHysical ACTivity cohort with Objective measurements (DPHACTO)) | Sleep, Work SB, Work LPA, Work MVPA, Leisure SB, Leisure LPA, Leisure MVPA | Actigraph GT3X+, sleep self-report (4 days) | Biomarkers |
| Gupta (2022) | Adults (n = 925) | Danish predominately blue collar workers (Physical wOrk DEmands and Prospective register-based Sickness Absence (PODESA) study. | Work SB, Work standing, Work LPA, Work MVPA, Leisure SB, Leisure standing, Leisure LPA, Leisure MVPA, Time in bed | Actigraph GT3X+, sleep self-report (5 workdays) | Pain/injury/illness |
| Hallman (2021) | Adults (n = 789) | Danish blue collar workers (Danish Physical Activity Cohort with Objective measurements (DPHACTO)) | Work sitting, Work standing, Work LPA, Work MVPA, Leisure MVPA, Leisure non-MVPA | Actigraph GTX+ (1-5 days) | Pain/injury/illness |
| Haszard (2020) | Children (n = 742) | New Zealand school children (the PLAY study) | Sleep, SB, LPA, MVPA | Actigraph GT3X (3-7 days) | Adiposity |
| Healy (2020) | Adolescents (n = 28) | US children with autism spectrum disorder | Sleep, SB, LPA, MVPA, Other behaviours equally | ActiGraph GT9X Link (7 days) | Adiposity |
| Hofman (2022) | Older adults (n = 1943) | Middle-aged and elderly Dutch adults (The Rotterdam Study) | Sleep, SB, LPA, MVPA | GENEActiv (7 days) | Mental health |
| Januario (2020) | Adults (n = 399) | Danish eldercare workers (Danish Observational Study of Eldercare work and musculoskeletal disorderS (DOSES)) | Work sitting, Work standing, Work LPA, Work MVPA | ActiGraph GT3X+ (at least 2 days) | Pain/injury/illness |
| Johansson (2022) | Adults (n = 652) | Healthy Danish adults (fifth examination of the Copenhagen City Heart Study) | Sleep, Work SB, Work Standing, Work Moving, Work Walking, Work HIPA, Leisure SB, Leisure Standing, Leisure Moving, Leisure Walking, Leisure HIPA | ActiGraph GT3X+ (5-7 days) | Adiposity, Biomarkers |
| Johansson (2020) | Adults, Older adults (n = 1053) | Representative sample of Copenhagen residents (fifth examination of the Copenhagen City Heart Study) | Sleep, SB, Standing, Moving, Walking, High-intensity PA | ActiGraph GT3X+ (5-7 days) | Adiposity, Biomarkers |
| Kandola (2022) | Adolescents (n = 4599) | Nationally representative birth cohort in the UK (The Millennium Cohort Study) | daily screen time, exercise, watching TV, using social media, playing video games, general computer use, individual exercise, team sports | Recall - not stated (2 days) | Mental health |
| Kandola (2021) | Adults (n = 60235) | UK middle-aged adults (UK Biobank) | Sleep, SB, LPA, MVPA | Axivity AX3, sleep self-report (3-7 days) | Mental health |
| Ketels (2020) | Adults (n = 309) | Belgian workers within the service and production sector (Flemish Employees' Physical Activity (FEPA) study) | Sleep, Work SB, Work Standing, Work LPA, Work MVPA, Leisure SB, Leisure standing, Leisure LPA, Leisure MVPA | Axivity AX3 (2-5 workdays) | Fitness |
| Kim (2021) | Adults (n = 1247) | Representative sample of US adults (Physical Activity Measurement Survey (PAMS) project) | Sleep, SB, LPA, MVPA | SenseWear Armband Mini (SWA); 24 physical activity recall (24PAR) (2 days) | Adiposity |
| Kitano (2022) | Adults (n = 1258) | Japanese office workers (Meiji Yasuda LifeStyle (MYLS) study) | Work SB, Work LPA, Work MVPA, Leisure SB, Leisure LPA, Leisure MVPA | Active style Pro HJA750-C, sleep self-report (10 days) | Adiposity, Biomarkers |
| Kitano (2020) | Adults (n = 1095) | Japanese office workers (Meiji Yasuda LifeStyle (MYLS) study) | Sleep, SB, LPA, MVPA | Active style Pro HJA750-C, sleep via self-report (10 days) | Mental health |
| Kuzik (2020) | Young children (n = 95) | Canadian preschool children (Parent-Child Movement Behaviours and Pre-School Children's Development study) | Sleep, SB, LPA, MVPA | ActiGraph wGT3X-BT (3-7 days) | Adiposity, General/perceived health, Mental health, Cognitive health |
| Larisch (2020) | Adults (n = 370) | Swedish office workers ("Physical activity and healthy brain functions" research project) | Sleep, SB, LPA, MVPA | ActiGraph GT3X, sleep via diary (5-7 days) | Mental health |
| Le (2022) | Adults (n = 361) | Australian young adults (Pooled sample of 3 studies: Activity, Coping, Emotions, Stress, and Sleep (ACES, N = 187); Diet, Exercise, Stress, Emotions, Speech, and Sleep (DESTRESS, N = 78); and Stress and Health Study (SHS, N = 96).) | Sleep, SB, LPA, MVPA, Time awake in bed | ActiGraph wGT3X-BT (7-15 days) | Mental health |
| Lee (2020) | Older adults (n = 1268) | US older adults. Offspring of original cohort (Framingham Offspring Study) | SB, LPA, MVPA | Actical model no. 198‐0200‐00 (4-7 days) | Chronic diseases/conditions |
| Lee (2020) | Older adults (n = 136) | US older women (Healthy Women Study) | Sleep, SB, LPA, MVPA | ActiGraph GT1M (SB, LPA, MVPA), Actiwatch-2 (sleep) (4-7 days) | Adiposity, Biomarkers |
| Lemos (2021) | Young children (n = 270) | Brazilian preschool children | Sleep, SB, LPA, MVPA | Actigraph WGT3-X, sleep via parental report | Fitness |
| Lewthwaite (2019) | Older adults (n = 95) | Australian older adults with chronic obstructive pulmonary disease (COPD) (Pooled sample: RCT and cohort study, not named) | Sleep, SB, LPA, MVPA, PA, Chores, Self-care, Socio-cultural, Passive transport, Work/study, Quiet time | MARCA (2 days at each timepoint) | General/perceived health, Mental health, Chronic diseases/conditions |
| Ma (2021) | Adults (n = 904) | US adults with Type 2 diabetes (NHANES 2003-2006) | SB, LPA, Morning MVPA, Evening MVPA | ActiGraph AM-7164 (1-7 days) | Mortality |
| Machida (2021) | Older adults (n = 485) | Japanese community-dwelling older adults (Neuron to Environmental Impact across Generations (NEIGE) study) | SB, LPA, MVPA | Active style Pro HJA-750C (4-7 days) | Cognitive health |
| Marshall (2021) | Children (n = 37) | Welsh children with/without type 1 diabetes | Sleep, SB, LPA, MVPA | GENEActiv (24 days) | Biomarkers |
| Marshall (2022) | Children (n = 101) | Welsh school children | Sleep, SB, LPA, MVPA | ActiGraph GT3X+, sleep via diary (3-7 days) | Biomarkers |
| Martins (2021) | Young children (n = 204) | Brazilian preschool children (Movement's Cool) | Preschool SB, Preschool LPA, Preschool MVPA | Actigraph WGT3-X (up to 7 days) | General/perceived health |
| Matricciani (2020) | Children, Adults (n = 2451) | Representative cohort of Australian children and their parents (Child Health CheckPoint study) | Sleep, SB, LPA, MVPA | GENEActiv (4-7 days) | Adiposity, Biomarkers |
| McGregor (2019) | Adults, Older adults (n = 1592) | Representative sample of 50-79yo US adults (2005-2006 NHANES) | Sleep, SB, LPA, MVPA | ActiGraph AM-7164; sleep self-report: 'How much sleep do you actually get at night on weekdays or workdays?' to the nearest hour (7) | Mortality |
| Migueles (2020) | Children (n = 93) | Overweight and obese Spanish children (ActiveBrains project) | Sleep, SB, LPA, MVPA | ActiGraph GT3X+. Sleep determined via algorithm, but sleep log also used. (4-7 days) | Cognitive health |
| Migueles (2022) | Young children (n = 315) | Swedish preschool children (MINISTOP trial) | SB, LPA, MPA, VPA | ActiGraph GT3X+ (3-7 days) | Adiposity, Fitness |
| Mota (2020) | Young children (n = 204) | Brazilian preschool children (Movement´s cool) | Sleep, SB, LPA, MVPA | Actigraph WGT3-X, sleep via parental report (7 days) | General/perceived health |
| Ng (2021) | Children (n = 1181) | Representative sample of Australian children (Child Health CheckPoint) | Sleep, SB, LPA, MVPA | GENEActiv | Adiposity, General/perceived health, Academic achievement |
| Olds (2018) | Adults (n = 105) | Australian adults transitioning to retirement (Life After Work (LAW)) | Sleep, PA, Chores, Quiet time, Screen time, Self-care, Social time, Passive transport, Work | Multimedia Activity Recall for Children and Adults (MARCA) (4 days at each timepoint) | General/perceived health, Mental health |
| Oviedoâ€Caro (2020) | Adults (n = 130) | Pregnant Spanish women in midterm (The PregnActive project) | Sleep, SB, LPA, MVPA | Sensewear Mini Armband (7 days) | Adiposity, Fitness |
| Pelclova (2020) | Older adults (n = 158) | Healthy Eastern European older women | SB, LPA, MVPA | ActiGraph GT1M (4-8 days) | Adiposity |
| Pelclova (2018) | Older adults (n = 314) | Healthy Eastern European older women | SB, LPA, MVPA | ActiGraph GT1M (4-8 days) | Adiposity |
| Powell (2020) | Older adults (n = 366) | Healthy Irish older adults (Mitchelstown Cohort Rescreen (MCR) Study) | Sleep, SB, LPA, MVPA, Standing | activPAL3 Micro (4-7 days) | Adiposity, Biomarkers |
| Rees-Punia (2021) | Adults (n = 549) | Racially diverse sample of US adults (Cancer Prevention Study-3 [CPS-3 Activity Validation Sub-study (AVSS)]) | Sleep, SB, LPA, MVPA | Actigraph GT3x+ (SB, LPA, MVPA), diary (sleep) (up to 7 days) | Adiposity |
| Ren (2022) | Adolescents (n = 1323) | Healthy Chinese adolescents (taken during COVID 19 pandemic) | Sleep, SB, LPA, MVPA | Survey, not stated | Mental health |
| Roscoe (2021) | Young children (n = 185) | British preschool children from a low SES region | SB, LPA, MVPA | GENEActiv (4 days) | General/perceived health |
| Von Rosen (2019) | Adults, Older adults (n = 851) | Swedish adults >50yo (Sweden Attitude Behaviour and Change (ABC) study) | SB, LPA, MVPA | ActiGraph 7164 (7) | Mortality |
| Rossen (2019) | Adults, Older adults (n = 175) | Swedish adults/older adults with diabetes or pre-diabetes (the Sophia step study) | SB, LPA, MVPA | ActiGraph GT1M (3-7 days) | Adiposity, Biomarkers |
| Rubin (2022) | Children, Adolescents (n = 88) | Healthy Czech children/adolescence (9.2yo at baseline and 14.6yo at follow-up) | SB, LPA, MPA, VPA, SB short bouts, SB middle bouts, SB long bouts | ActiGraph GT3X (4-7 days) |  |
| Sampasa-Kanyinga (2021) | Adolescents (n = 14620) | Canadian adolescent school students (The COMPASS study) | Sleep, MVPA, Screen time | Survey, not stated | Mental health |
| Sandborg (2022) | Adults (n = 272) | Pregnant Swedish women in midterm pregnancy (HealthyMoms trial) | Sleep, SB, LPA, MVPA | ActiGraph wGT3x-BT (1-7 days) | Adiposity, Biomarkers |
| Smith (2020) | Children (n = 258) | British and Iranian children | Sleep, SB, LPA, MVPA | GENEActiv (4-7 days) | General/perceived health |
| Stevens (2019) | Adults (n = 840) | Danish blue-collar workers (Danish Physical Activity Cohort with Objective Measurements (DPhacto)) | Work SB, Work standing, Work LPA, Work MVPA, Non-work time | ActiGraph GT3X+ | Pain/injury/illness |
| Su (2022) | Adults (n = 1475) | Chinese young adults (taken during COVID-19 pandemic) | Sleep, SB, LPA, MVPA | IPAQ-SF (SB, LPA, MVPA); Pittsburgh Sleep Quality Index (sleep) | Mental health |
| Swindell (2020) | Adults (n = 1462) | Multi-nation sample of OW/obese adults with pre-diabetes (PREVention of diabetes through lifestyle Intervention and population studies in Europe and around the World (PREVIEW) study) | Sleep, SB, LPA, MVPA | ActiSleep+ (4-7 days) | Adiposity, Biomarkers |
| Talarico (2018) | Children (n = 434) | Canadian school children | Sleep, SB, LPA, MVPA | Actical (4-7 days) | Adiposity |
| Taylor (2020) | Children (n = 574) | Predominantly low SES New Zealand children (PLAY) | Sleep, SB, LPA, MVPA, Time awake after sleep onset | ActiGraph GT3X (up to 8 days) | Adiposity |
| Taylor (2018) | Young children (n = 346) | Healthy New Zealand young children (Prevention of Overweight in Infancy (POI) study) | Sleep, SB, LPA, MVPA | Actical (7 days) | Adiposity, Other |
| Tsunoda (2021) | Adults (n = 1914) | Japanese adults (Meiji Yasuda LifeStyle study) | Sleep, SB, LPA, MVPA | Active style Pro HJA-750C, sleep via questionnaire (At least 4 days) | Chronic diseases/conditions |
| Verhoog (2020) | Adults, Older adults (n = 1934) | Population based cohort of Dutch adults (2011-2016 Rotterdam Study) | Sleep, SB, LPA, MVPA | GENEActiv (7 days) | General/perceived health |
| Verswijveren (2022) | Children (n = 782) | Healthy Australian Children (Pooled 2 studies (Transform-Us! & LOOK)) | SB, LPA, MPA, VPA, SB short bouts (<5 min), SB long bouts (≥5 min), LPA short bouts (<1 min), LPA longs bouts (≥1 min), MPA short bouts (<1min), MPA long bouts (≥1 min), VPA short bouts (<1min), VPA long bouts (≥1 min), Other (sleep & non-wear) | ActiGraph GT1M & GT3X (4-8 days) | Adiposity |
| Walmsley (2021) | Adults (n = 87498) | Large prospective cohort of UK adults (UK Biobank) | Sleep, SB, LPA, MVPA | Axivity AX3 (3-7 days) | Chronic diseases/conditions |
| Whitaker (2021) | Adults (n = 1970) | Ethnically diverse sample of middle-aged adults from 4 regions of the USA (The Coronary Artery Risk Development in Young Adults (CARDIA) Study) | Sleep, SB, LPA, MVPA | ActiGraph 7164, sleep inferred via non-wear time (4-7 days) | Cognitive health |
| Winkler (2018) | Adults (n = 136) | Australian office workers (Stand Up Victoria) | Sitting, Standing, Stepping, Other (sleep, time in bed, non-wear) | activPAL3 (up to 7 days) | Adiposity, Biomarkers |
| Yerramalla (2021) | Older adults (n = 3319) | British older adults from cohort study. Originally middle-aged civil servants working in London (recruited in 1985-88) (Whitehall II study) | Sleep, SB, LPA, MVPA | GENEActiv (4-7 days) | Chronic diseases/conditions |
| Zhang (2022) | Adolescents (n = 241) | Healthy Chinese adolescents | Sleep, SB, LPA, MVPA | ActiGraph GT3X+, sleep inferred by non-wear time (4-7 days) |  |

LPA, light physical activity; MVPA, moderate-to-vigorous physical activity; PA, physical activity; SB, sedentary behaviour
