# Supplementary material for: The association between reallocations of time and health using compositional data analysis: a systematic scoping review with an interactive data exploration interface
Source: Int J Behav Nutr Phys Act. 2023 Oct 19;20:127. doi: 10.1186/s12966-023-01526-x (PMC10588100; doi:10.1186/s12966-023-01526-x)
Supplement: Supplementary file 4 — Supplementary Material 4: Table S3. Newcastle-Ottawa Scale gradings for cross-sectional studies [file 12966_2023_1526_MOESM4_ESM.docx]

Table S3. Newcastle-Ottawa Scale gradings for cross-sectional studies

| Study | Representativeness of the sample | Sample size | Comparability of non-respondents | Ascertainment of exposure | Comparability | Assessment of outcome | Statistical test | Total |
| --- | --- | --- | --- | --- | --- | --- | --- | --- |
| Amagasa (2020) | * | 0 | 0 | * | ** | ** | 0 | 6 |
| Atkin (2021) | * | 0 | 0 | * | ** | * | * | 6 |
| Bezerra (2020) | * | 0 | 0 | * | ** | * | * | 6 |
| Bianchim (2022) | 0 | 0 | 0 | * | 0 | ** | 0 | 3 |
| Biddle (2018) | * | 0 | * | * | ** | ** | * | 8 |
| Biddle (2021) | * | 0 | * | * | ** | ** | * | 8 |
| Blodgett (2022) | * | 0 | 0 | * | ** | ** | * | 7 |
| Booker (2022) | * | 0 | 0 | 0 | ** | ** | * | 6 |
| Bourke (2022) | * | 0 | 0 | * | ** | ** | * | 7 |
| Brakenridge (2021) | * | 0 | 0 | * | ** | ** | * | 7 |
| Brown (2021) | 0 | 0 | 0 | * | ** | * | * | 5 |
| Burns (2019) | 0 | 0 | 0 | * | ** | * | 0 | 4 |
| Cabanas-Sanchez (2021) | * | 0 | 0 | * | ** | * | * | 6 |
| Carson (2016) | * | 0 | 0 | * | ** | ** | 0 | 6 |
| Chao (2022) | 0 | 0 | 0 | 0 | ** | * | * | 4 |
| Chastin (2015) | * | 0 | * | * | ** | ** | 0 | 7 |
| Curtis (2020) | 0 | 0 | * | * | ** | * | * | 6 |
| del Pozo Cruz (2020) | * | 0 | 0 | * | ** | * | * | 6 |
| del Pozo-Cruz (2022) | 0 | 0 | 0 | * | ** | ** | * | 6 |
| Domingues (2022) | 0 | 0 | 0 | * | ** | ** | 0 | 5 |
| Dumuid (2018) | 0 | * | * | * | ** | ** | * | 8 |
| Dumuid (2018) | * | 0 | * | * | ** | * | 0 | 6 |
| Dumuid (2022) | 0 | 0 | 0 | * | ** | * | * | 5 |
| Dumuid (2022) | * | 0 | 0 | * | ** | ** | 0 | 6 |
| Dumuid (2018) | * | 0 | 0 | * | * | ** | * | 6 |
| Dumuid (2018) | * | 0 | 0 | * | ** | ** | * | 7 |
| Estevan (2022) | 0 | * | 0 | * | ** | * | * | 6 |
| Fairclough (2018) | * | 0 | 0 | * | ** | ** | * | 7 |
| Fairclough (2017) | * | 0 | 0 | * | ** | ** | 0 | 6 |
| Fariclough (2022) | 0 | 0 | 0 | * | ** | ** | * | 6 |
| Fairclough (2021) | 0 | 0 | 0 | * | ** | * | * | 5 |
| Farrahi (2021) | * | 0 | 0 | * | ** | ** | * | 7 |
| Gaba (2021) | 0 | * | 0 | * | * | ** | * | 6 |
| Gaba (2020) | 0 | * | 0 | * | * | ** | * | 6 |
| Germano-Soares (2021) | 0 | 0 | 0 | * | * | ** | * | 5 |
| Giurgiu (2022) | 0 | 0 | 0 | * | ** | * | 0 | 4 |
| Gupta (2018) | 0 | 0 | 0 | * | ** | ** | * | 6 |
| Gupta (2019) | 0 | 0 | 0 | * | ** | ** | * | 6 |
| Haszard (2020) | * | 0 | 0 | * | 0 | ** | * | 5 |
| Healy (2020) | 0 | 0 | 0 | * | ** | ** | 0 | 5 |
| Hofman (2022) | * | 0 | 0 | * | ** | * | * | 6 |
| Januario (2020) | 0 | 0 | 0 | * | ** | * | * | 5 |
| Johansson (2022) | * | 0 | * | * | ** | ** | * | 8 |
| Johansson (2020) | * | 0 | * | * | ** | ** | * | 8 |
| Ketels (2020) | 0 | 0 | 0 | * | ** | ** | * | 6 |
| Kim (2021) | * | 0 | 0 | * | ** | ** | * | 7 |
| Kitano (2022) | 0 | 0 | 0 | * | ** | ** | * | 6 |
| Kitano (2020) | 0 | 0 | 0 | * | ** | * | 0 | 4 |
| Kuzik (2020) | 0 | 0 | 0 | * | ** | ** | * | 6 |
| Larisch (2020) | 0 | 0 | 0 | * | ** | * | * | 5 |
| Le (2022) | 0 | 0 | 0 | * | ** | * | * | 5 |
| Lee (2020) | * | 0 | * | * | ** | ** | 0 | 7 |
| Lee (2020) | 0 | 0 | 0 | * | ** | ** | 0 | 5 |
| Lemos (2021) | * | 0 | 0 | * | ** | ** | * | 7 |
| Machida (2021) | * | 0 | 0 | * | ** | ** | 0 | 6 |
| Marshall (2021) | 0 | 0 | 0 | * | ** | ** | 0 | 5 |
| Marshall (2022) | 0 | 0 | * | * | * | ** | 0 | 5 |
| Martins (2021) | 0 | * | 0 | * | ** | * | * | 6 |
| Matricciani (2020) | * | 0 | 0 | * | ** | ** | 0 | 6 |
| Migueles (2020) | 0 | 0 | 0 | * | * | ** | * | 5 |
| Migueles (2022) | * | 0 | 0 | * | ** | ** | * | 7 |
| Mota (2020) | 0 | 0 | 0 | * | ** | ** | * | 6 |
| Ng (2021) | * | 0 | * | * | ** | ** | 0 | 7 |
| Oviedo-Caro (2020) | 0 | 0 | 0 | * | ** | ** | * | 6 |
| Pelclova (2018) | 0 | 0 | 0 | * | ** | ** | 0 | 5 |
| Powell (2020) | * | 0 | * | * | ** | ** | * | 8 |
| Ren (2022) | 0 | 0 | 0 | 0 | ** | * | 0 | 3 |
| Roscoe (2021) | 0 | 0 | 0 | * | ** | ** | 0 | 5 |
| Rossen (2019) | 0 | 0 | 0 | * | ** | ** | 0 | 5 |
| Sandborg (2022) | 0 | 0 | 0 | * | ** | ** | * | 6 |
| Smith (2020) | 0 | 0 | 0 | * | ** | ** | * | 6 |
| Stevens (2019) | 0 | 0 | 0 | * | ** | * | * | 5 |
| Su (2022) | 0 | 0 | 0 | 0 | ** | * | * | 4 |
| Swindell (2020) | 0 | 0 | 0 | * | ** | ** | * | 6 |
| Talarico (2018) | * | 0 | 0 | * | ** | ** | 0 | 6 |
| Taylor (2020) | 0 | 0 | 0 | * | ** | ** | * | 6 |
| Taylor (2018) | 0 | 0 | 0 | * | ** | ** | * | 6 |
| Tsunoda (2021) | 0 | 0 | 0 | * | ** | ** | * | 6 |
| Verhoog (2020) | * | 0 | 0 | * | ** | * | * | 6 |
| Verswijveren (2022) | 0 | 0 | 0 | * | ** | ** | 0 | 5 |
| Zhang (2022) | 0 | 0 | 0 | * | ** | ** | * | 6 |

Grading for Newcastle-Ottawa scale for cross-sectional studies

**NEWCASTLE - OTTAWA QUALITY ASSESSMENT SCALE**

**(adapted for cross sectional studies)**

**Selection:** (Maximum 5 stars)

1. Representativeness of the sample:
   - 1. Truly representative of the average in the target population. * (all subjects or random sampling)
     2. Somewhat representative of the average in the target population. * (non-random sampling)
     3. Selected group of users.
     4. No description of the sampling strategy.
2. Sample size:
   - 1. Justified and satisfactory. *
     2. Not justified.
3. Non-respondents:
   - 1. Comparability between respondents and non-respondents characteristics is established, and the response rate is satisfactory. *
     2. The response rate is unsatisfactory, or the comparability between respondents and non-respondents is unsatisfactory.
     3. No description of the response rate or the characteristics of the responders and the non-responders.
4. Ascertainment of the exposure (risk factor):
   1. Validated measurement tool. **
   2. Non-validated measurement tool, but the tool is available or described.*
   3. No description of the measurement tool.

**Comparability:** (Maximum 2 stars)

1. The subjects in different outcome groups are comparable, based on the study design or analysis. Confounding factors are controlled.
   1. The study controls for age and sex. *
   2. The study control for any additional factor. *

**Outcome:** (Maximum 3 stars)

1. Assessment of the outcome:
   1. Independent blind assessment. **
   2. Record linkage. **
   3. Self report. *
   4. No description.
2. Statistical test:
   1. The statistical test used to analyze the data is clearly described and appropriate, and the measurement of the association is presented, including confidence intervals and the probability level (p value). *
   2. The statistical test is not appropriate, not described or incomplete.

Note: for comparability, age and sex/gender were required for 1 star, and any additional factor for a second.

**Gradings**

Studies that scored a total ≤4 were considered to be poor quality; studies that scored a total 5-6 were considered fair quality; Studies that scored a total ≥7 were considered good quality.
