# Supplementary material for: The association between reallocations of time and health using compositional data analysis: a systematic scoping review with an interactive data exploration interface
Source: Int J Behav Nutr Phys Act. 2023 Oct 19;20:127. doi: 10.1186/s12966-023-01526-x (PMC10588100; doi:10.1186/s12966-023-01526-x)
Supplement: Supplementary file 5 — Supplementary Material 5: Table S4. Newcastle-Ottawa Scale gradings for prospective studies [file 12966_2023_1526_MOESM5_ESM.docx]

Table S4. Newcastle-Ottawa Scale gradings for prospective studies

| Study | Representativeness | Selection of the non-exposed | Ascertainment of exposure | Outcome not present | Comparability of cohorts | Assessment of outcome | Follow-up length | Adequacy of follow-up | Total |
| --- | --- | --- | --- | --- | --- | --- | --- | --- | --- |
| Cabanas-Sanchez (2021) | * | * | * | * | ** | 0 | * | 0 | 7 |
| Chastin (2021) | * | * | * | * | ** | * | * | 0 | 8 |
| Chong (2022) | * | * | * | 0 | * | 0 | * | 0 | 5 |
| Clarke (2021) | * | * | * | * | ** | * | * | * | 9 |
| Gaba (2021) | 0 | * | * | * | * | * | * | 0 | 6 |
| Gupta (2020) | 0 | * | * | * | ** | * | * | 0 | 7 |
| Gupta (2022) | 0 | * | * | * | ** | * | * | 0 | 7 |
| Hallman (2021) | 0 | * | * | * | ** | 0 | * | 0 | 6 |
| Kandola (2022) | * | * | * | * | ** | 0 | * | 0 | 7 |
| Kandola (2021) | * | * | * | * | ** | 0 | * | 0 | 7 |
| Lewthwaite (2019) | 0 | * | * | * | ** | * | 0 | * | 7 |
| Ma (2021) | * | * | * | * | ** | * | * | 0 | 8 |
| McGregor (2019) | * | * | * | * | ** | * | * | * | 9 |
| Migueles (2022) | * | * | * | * | ** | * | * | 0 | 8 |
| Olds (2018) | 0 | * | * | * | * | 0 | * | * | 6 |
| Pelclova (2020) | 0 | * | * | * | ** | * | * | 0 | 7 |
| Rees-Punia (2021) | 0 | * | * | 0 | ** | 0 | * | * | 6 |
| Von Rosen (2019) | * | * | * | * | ** | * | * | * | 9 |
| Rubin (2022) | 0 | * | * | * | * | * | * | 0 | 6 |
| Sampasa-Kanyinga (2021) | 0 | * | 0 | 0 | ** | 0 | * | 0 | 4 |
| Sandborg (2022) | 0 | * | * | 0 | ** | * | 0 | 0 | 5 |
| Taylor (2018) | 0 | * | * | * | ** | * | * | 0 | 7 |
| Walmsley (2021) | * | * | * | * | ** | * | * | * | 9 |
| Whitaker (2021) | * | * | * | 0 | ** | * | * | 0 | 7 |
| Yerramalla (2021) | 0 | * | * | * | ** | * | * | 0 | 7 |

Grading for Newcastle-Ottawa scale for prospective studies

**Newcastle-Ottawa Quality Assessment Form for Cohort Studies**

**Selection**

1. Representativeness of the exposed cohort
   1. Truly representative ***(one star)***
   2. Somewhat representative ***(one star)***
   3. Selected group
   4. No description of the derivation of the cohort
2. Selection of the non-exposed cohort
   1. Drawn from the same community as the exposed cohort ***(one star)***
   2. Drawn from a different source
   3. No description of the derivation of the non exposed cohort
3. Ascertainment of exposure
   1. Objective time-use measure ***(one star)***
   2. 24-h time-use recall ***(one star)***
   3. Self-report
   4. No description
   5. Other
4. Demonstration that outcome of interest was not present at start of study
   1. Yes ***(one star)***
   2. No

**Comparability**

1. Comparability of cohorts on the basis of the design or analysis controlled for confounders
   1. The study controls for age and sex ***(one star)***
   2. Study controls for other factors ***(one star)***
   3. Cohorts are not comparable on the basis of the design or analysis controlled for confounders

**Outcome**

1. Assessment of outcome
   1. Independent blind assessment ***(one star)***
   2. Record linkage ***(one star)***
   3. Self report
   4. No description
   5. Other
2. Was follow-up long enough for outcomes to occur
   1. Yes ***(one star)***
   2. No
3. Adequacy of follow-up of cohorts
   1. Complete follow up- all subject accounted for ***(one star)***
   2. Subjects lost to follow up unlikely to introduce bias- number lost less than or equal to 20% or description of those lost suggested no different from those followed. ***(one star)***
   3. Follow up rate less than 80% and no description of those lost
   4. No statement

**Gradings**

Studies that scored a total ≤5 were considered to be poor quality; studies that scored a total 6-7 were considered fair quality; Studies that scored a total ≥8 were considered good quality.
