# Supplementary material for: The association between reallocations of time and health using compositional data analysis: a systematic scoping review with an interactive data exploration interface
Source: Int J Behav Nutr Phys Act. 2023 Oct 19;20:127. doi: 10.1186/s12966-023-01526-x (PMC10588100; doi:10.1186/s12966-023-01526-x)
Supplement: Supplementary file 6 — Supplementary Material 6: Table S5. Summary of each study contributing to grading [file 12966_2023_1526_MOESM6_ESM.docx]

Table S5. Summary of each study contributing to grading

| Study | Sample | Health outcome | Sleep to SB | Sleep to LPA | Sleep to MVPA | SB to Sleep | SB to LPA | SB to MVPA | LPA to Sleep | LPA to SB | LPA to MVPA | MVPA to Sleep | MVPA to SB | MVPA to LPA |
| --- | --- | --- | --- | --- | --- | --- | --- | --- | --- | --- | --- | --- | --- | --- |
|  | **Adiposity** |  |  |  |  |  |  |  |  |  |  |  |  |  |
| Dumuid (2018) | Children | % Fat-free mass | ↓ | ↓ | ↑ | ↔ | ↓ | ↑ | ↑ | ↑ | ↑ | ↓ | ↓ | ↓ |
| Dumuid (2018) | Children | % Non-truncal fat | ↓ | ↓ | ↑ | ↑ | ↔ | ↑ | ↑ | ↔ | ↑ | ↓ | ↓ | ↓ |
| Dumuid (2018) | Children | % Truncal fat | ↔ | ↓ | ↑ | ↔ | ↓ | ↑ | ↑ | ↑ | ↑ | ↓ | ↓ | ↓ |
| Farrahi (2021) | Adults | %BF | ↔ | ↑ | ↑ | ↔ | ↑ | ↑ | ↓ | ↓ | ↑ | ↓ | ↓ | ↓ |
| Dumuid (2018) | Children | %BF | ↔ | ↓ | ↑ | ↔ | ↔ | ↑ | ↑ | ↔ | ↑ | ↓ | ↓ | ↓ |
| Gaba (2020) | Children | %BF |  |  |  |  | ↔ | ↔ |  | ↔ |  |  | ↔ |  |
| Rubin (2022) | Children/Adolescents | %BF^#^ |  |  |  |  | ↔ |  |  |  |  |  |  |  |
| Pelclova (2020) | Older adults | %BF^#^ |  |  |  |  | ↑ | ↑ |  | ↓ | ↔ |  | ↓ | ↓ |
| Powell (2020) | Older adults | %BF | ↔ | ↑ | ↔ | ↔ | ↑ | ↔ | ↓ | ↓ | ↓ | ↔ | ↔ | ↔ |
| Swindell (2020) | Adults | %BF | ↔ | ↔ | ↑ | ↔ | ↑ | ↑ | ↔ | ↓ | ↑ | ↓ | ↓ | ↓ |
| Matricciani (2020) | Adults | BMI | ↓ | ↔ | ↑ | ↑ | ↑ | ↑ | ↔ | ↓ | ↑ | ↓ | ↓ | ↓ |
| Matricciani (2020) | Children | BMI | ↓ | ↓ | ↑ | ↑ | ↔ | ↑ | ↑ | ↔ | ↑ | ↓ | ↓ | ↓ |
| Dumuid (2018) | Older adults | BMI | ↔ | ↔ | ↑ | ↔ | ↔ | ↑ | ↔ | ↔ | ↑ | ↓ | ↓ | ↓ |
| Pelclova (2020) | Older adults | BMI^#^ |  |  |  |  | ↑ | ↑ |  | ↓ | ↑ |  | ↓ | ↓ |
| Powell (2020) | Older adults | BMI | ↓ | ↑ | ↔ | ↑ | ↑ | ↔ | ↓ | ↓ | ↓ | ↔ | ↔ | ↔ |
| Biddle (2021) | Adults | BMI |  |  |  |  | ↑ | ↑ |  |  |  |  |  |  |
| Healy (2020) | Children | BMI | ↔ | ↓ | ↔ | ↔ | ↔ | ↔ | ↑ | ↔ | ↔ | ↔ | ↔ | ↔ |
| Oviedo-Caro (2020) | Adults | BMI | ↔ | ↔ | ↔ | ↔ | ↑ | ↑ | ↔ | ↓ | ↔ | ↓ | ↓ | ↔ |
| Sandborg (2022) | Adults | BMI |  |  |  |  | ↔ | ↔ |  | ↔ | ↔ |  | ↔ | ↔ |
| Sandborg (2022) | Adults | BMI^#^ |  |  |  |  | ↔ | ↔ |  | ↔ | ↔ |  | ↔ | ↔ |
| Swindell (2020) | Adults | BMI | ↓ | ↔ | ↑ | ↑ | ↑ | ↑ | ↔ | ↓ | ↑ | ↓ | ↓ | ↓ |
| Rees-Punia (2021) | Adults | Body mass^#^ | ↔ | ↔ | ↔ | ↔ | ↔ | ↔ | ↔ | ↔ | ↔ |  |  |  |
| Farrahi (2021) | Adults | Fat mass | ↔ | ↑ | ↑ | ↑ | ↑ | ↑ | ↓ | ↓ | ↑ | ↓ | ↓ | ↓ |
| Powell (2020) | Older adults | Fat mass | ↔ | ↑ | ↔ | ↔ | ↑ | ↔ | ↓ | ↔ | ↓ | ↔ | ↓ | ↔ |
| Gaba (2021) | Older adults | FFMI^#^ |  |  |  |  | ↔ | ↔ |  |  |  |  |  |  |
| Sandborg (2022) | Adults | FFMI |  |  |  |  | ↔ | ↔ |  | ↔ | ↔ |  | ↔ | ↔ |
| Sandborg (2022) | Adults | FFMI^#^ |  |  |  |  | ↔ | ↔ |  | ↔ | ↔ |  | ↔ | ↔ |
| Gaba (2020) | Children | FMI |  |  |  |  | ↔ | ↔ |  | ↔ |  |  | ↔ |  |
| Rubin (2022) | Children/Adolescents | FMI^#^ |  |  |  |  | ↔ |  |  |  |  |  |  |  |
| Gaba (2021) | Older adults | FMI^#^ |  |  |  |  | ↔ | ↔ |  |  |  |  |  |  |
| Oviedo-Caro (2020) | Adults | FMI | ↔ | ↑ | ↑ | ↔ | ↑ | ↑ | ↓ | ↓ | ↔ | ↓ | ↓ | ↔ |
| Sandborg (2022) | Adults | FMI |  |  |  |  | ↔ | ↔ |  | ↔ | ↔ |  | ↔ | ↔ |
| Sandborg (2022) | Adults | FMI^#^ |  |  |  |  | ↔ | ↔ |  | ↔ | ↔ |  | ↔ | ↔ |
| Oviedo-Caro (2020) | Adults | Sum of skinfolds | ↔ | ↑ | ↑ | ↔ | ↑ | ↑ | ↓ | ↓ | ↔ | ↓ | ↓ | ↔ |
| Gaba (2020) | Children | Visceral adipose tissue |  |  |  |  | ↔ | ↑ |  | ↔ |  |  | ↓ |  |
| Rubin (2022) | Children/Adolescents | Visceral adipose tissue^#^ |  |  |  |  | ↔ |  |  |  |  |  |  |  |
| Gaba (2021) | Older adults | Visceral adipose tissue^#^ |  |  |  |  | ↔ | ↔ |  |  |  |  |  |  |
| Dumuid (2018) | Older adults | W:Hip | ↔ | ↔ | ↑ | ↔ | ↔ | ↑ | ↔ | ↔ | ↑ | ↓ | ↓ | ↓ |
| Farrahi (2021) | Adults | WC | ↔ | ↑ | ↑ | ↑ | ↑ | ↑ | ↓ | ↓ | ↑ | ↓ | ↓ | ↓ |
| Biddle (2021) | Adults | WC |  |  |  |  | ↑ | ↑ |  |  |  |  |  |  |
| Swindell (2020) | Adults | WC | ↓ | ↔ | ↑ | ↑ | ↑ | ↑ | ↔ | ↓ | ↑ | ↓ | ↓ | ↓ |
| Domingues (2022) | Adolescents | zBMI | ↔ | ↔ | ↔ | ↔ | ↔ | ↔ | ↔ | ↔ | ↔ |  |  |  |
| Curtis (2020) | Adults | zBMI | ↔ | ↔ | ↑ | ↔ | ↔ | ↑ | ↔ | ↔ | ↑ | ↓ | ↓ | ↓ |
| Haszard (2020) | Children | zBMI | ↓ | ↓ | ↑ | ↑ | ↓ | ↑ | ↑ | ↑ | ↑ | ↓ | ↓ | ↓ |
| Kuzik (2020) | Young children | zBMI | ↔ |  | ↔ | ↔ | ↔ | ↓ |  | ↔ | ↔ | ↔ | ↑ | ↔ |
|  | **Biomarkers** |  |  |  |  |  |  |  |  |  |  |  |  |  |
| Brakenridge (2021) | Adults | 2-h glucose | ↔ | ↔ | ↔ | ↔ | ↔ | ↑ | ↔ | ↔ | ↔ | ↓ | ↓ | ↔ |
| Biddle (2018) | Adults | 2-h glucose | ↔ | ↔ | ↑ | ↔ | ↔ | ↑ | ↔ | ↔ | ↑ | ↓ | ↓ | ↓ |
| Farrahi (2021) | Adults | 2-h insulin | ↔ | ↑ | ↑ | ↔ | ↑ | ↑ | ↓ | ↓ | ↑ | ↓ | ↓ | ↓ |
| Biddle (2018) | Adults | 2-h insulin | ↔ | ↔ | ↑ | ↔ | ↔ | ↑ | ↔ | ↔ | ↑ | ↓ | ↓ | ↓ |
| Biddle (2021) | Adults | CMR Score |  |  |  |  | ↑ | ↑ |  |  |  |  |  |  |
| Matricciani (2020) | Adults | Diastolic BP | ↔ | ↔ | ↔ | ↔ | ↔ | ↑ | ↔ | ↔ | ↔ | ↔ | ↓ | ↔ |
| Matricciani (2020) | Children | Diastolic BP | ↔ | ↔ | ↑ | ↔ | ↔ | ↑ | ↔ | ↔ | ↑ | ↓ | ↓ | ↓ |
| Sandborg (2022) | Adults | Diastolic BP |  |  |  |  | ↔ | ↔ |  | ↔ | ↔ |  | ↔ | ↔ |
| Sandborg (2022) | Adults | Diastolic BP^#^ |  |  |  |  | ↔ | ↔ |  | ↔ | ↔ |  | ↔ | ↔ |
| Brakenridge (2021) | Adults | Fasting glucose | ↔ | ↔ | ↔ | ↔ | ↔ | ↔ | ↔ | ↔ | ↔ | ↔ | ↔ | ↔ |
| Farrahi (2021) | Adults | Fasting glucose | ↔ | ↑ | ↑ | ↔ | ↑ | ↑ | ↓ | ↓ | ↑ | ↓ | ↓ | ↓ |
| Powell (2020) | Older adults | Fasting glucose | ↔ | ↔ | ↔ | ↔ | ↔ | ↔ | ↔ | ↔ | ↔ | ↔ | ↔ | ↔ |
| Biddle (2018) | Adults | Fasting glucose | ↔ | ↔ | ↔ | ↔ | ↔ | ↔ | ↔ | ↔ | ↔ | ↔ | ↔ | ↔ |
| Sandborg (2022) | Adults | Fasting glucose |  |  |  |  | ↔ | ↔ |  | ↔ | ↔ |  | ↔ | ↔ |
| Sandborg (2022) | Adults | Fasting glucose^#^ |  |  |  |  | ↔ | ↔ |  | ↔ | ↔ |  | ↔ | ↔ |
| Biddle (2018) | Adults | Fasting insulin | ↔ | ↔ | ↔ | ↔ | ↔ | ↔ | ↔ | ↔ | ↔ | ↔ | ↔ | ↔ |
| Biddle (2021) | Adults | HbA1c |  |  |  |  | ↔ | ↔ |  |  |  |  |  |  |
| Powell (2020) | Older adults | HDL cholesterol | ↔ | ↔ | ↔ | ↔ | ↔ | ↔ | ↔ | ↔ | ↔ | ↔ | ↔ | ↔ |
| Biddle (2021) | Special population | HDL cholesterol |  |  |  |  | ↑ | ↑ |  |  |  |  |  |  |
| Biddle (2018) | Adults | HOMA-IS | ↔ | ↔ | ↑ | ↔ | ↔ | ↔ | ↔ | ↔ | ↔ | ↓ | ↔ | ↔ |
| Sandborg (2022) | Adults | HOMA IR |  |  |  |  | ↔ | ↔ |  | ↔ | ↔ |  | ↔ | ↔ |
| Sandborg (2022) | Adults | HOMA IR^#^ |  |  |  |  | ↑ | ↔ |  | ↓ | ↔ |  | ↔ | ↔ |
| Booker (2022) | Adults | hs-CRP concentration | ↔ |  |  | ↔ |  |  |  |  |  |  |  |  |
| Powell (2020) | Older adults | LDL cholesterol | ↔ | ↔ | ↔ | ↔ | ↔ | ↔ | ↔ | ↔ | ↔ | ↔ | ↔ | ↔ |
| Biddle (2021) | Adults | LDL cholesterol |  |  |  |  | ↔ | ↔ |  |  |  |  |  |  |
| Farrahi (2021) | Adults | LDL/HDL ratio | ↔ | ↑ | ↑ | ↔ | ↑ | ↑ | ↓ | ↓ | ↑ | ↓ | ↓ | ↓ |
| Swindell (2020) | Adults | Log10 hs-CRP | ↔ | ↔ | ↑ | ↔ | ↔ | ↑ | ↔ | ↔ | ↑ | ↓ | ↓ | ↓ |
| Biddle (2018) | Adults | Matsuda-ISI | ↔ | ↔ | ↑ | ↔ | ↔ | ↑ | ↔ | ↔ | ↑ | ↓ | ↓ | ↓ |
| Sandborg (2022) | Adults | MetS score |  |  |  |  | ↔ | ↑ |  | ↔ | ↑ |  | ↓ | ↓ |
| Sandborg (2022) | Adults | MetS score^#^ |  |  |  |  | ↑ | ↔ |  | ↓ | ↔ |  | ↔ | ↔ |
| Matricciani (2020) | Adults | MetS score | ↔ | ↔ | ↑ | ↔ | ↔ | ↑ | ↔ | ↔ | ↑ | ↓ | ↓ | ↓ |
| Matricciani (2020) | Children | MetS score | ↓ | ↓ | ↑ | ↑ | ↔ | ↑ | ↑ | ↔ | ↑ | ↓ | ↓ | ↓ |
| Matricciani (2020) | Adults | sqrt HOMA-IR | ↔ | ↑ | ↔ | ↔ | ↑ | ↔ | ↓ | ↓ | ↔ | ↔ | ↔ | ↔ |
| Matricciani (2020) | Adults | sqrt Insulin | ↔ | ↑ | ↑ | ↔ | ↑ | ↑ | ↓ | ↓ | ↔ | ↓ | ↓ | ↔ |
| Swindell (2020) | Adults | sqrt Triglycerides | ↔ | ↔ | ↑ | ↔ | ↔ | ↑ | ↔ | ↔ | ↑ | ↓ | ↓ | ↓ |
| Swindell (2020) | Adults | Systolic BP | ↓ | ↔ | ↔ | ↓ | ↔ | ↑ | ↔ | ↔ | ↔ | ↔ | ↓ | ↔ |
| Swindell (2020) | Children | Systolic BP | ↔ | ↔ | ↔ | ↔ | ↔ | ↔ | ↔ | ↔ | ↔ | ↔ | ↔ | ↔ |
| Gupta (2018) | Adults | Systolic BP | ↓ |  |  |  |  |  |  |  |  |  |  |  |
| Sandborg (2022) | Adults | Systolic BP |  |  |  |  | ↔ | ↔ |  | ↔ | ↔ |  | ↔ | ↔ |
| Sandborg (2022) | Adults | Systolic BP^#^ |  |  |  |  | ↔ | ↔ |  | ↔ | ↔ |  | ↔ | ↔ |
| Powell (2020) | Older adults | Total cholesterol | ↔ | ↔ | ↔ | ↔ | ↔ | ↔ | ↔ | ↔ | ↔ | ↔ | ↔ | ↔ |
| Biddle (2021) | Adults | Total cholesterol |  |  |  |  | ↔ | ↔ |  |  |  |  |  |  |
| Farrahi (2021) | Adults | total/HDL cholesterol ratio | ↔ | ↑ | ↑ | ↔ | ↑ | ↑ | ↓ | ↓ | ↑ | ↓ | ↓ | ↓ |
| Powell (2020) | Older adults | Triglycerides | ↔ | ↔ | ↔ | ↔ | ↔ | ↔ | ↔ | ↔ | ↔ | ↔ | ↔ | ↔ |
| Biddle (2021) | Adults | Triglycerides |  |  |  |  | ↑ | ↑ |  |  |  |  |  |  |
|  | **Mental health** |  |  |  |  |  |  |  |  |  |  |  |  |  |
| Kandola (2021) | Adults | Anxiety score^#^ |  |  |  | ↑ | ↓ | ↑ |  |  |  |  |  |  |
| Chao (2022) | Adults | Anxiety symptoms | ↔ | ↑ | ↑ | ↔ | ↑ | ↑ | ↓ | ↓ | ↔ | ↓ | ↓ | ↓ |
| Hofman (2022) | Adults/Older adults | Anxiety symptoms | ↔ | ↔ | ↔ | ↔ | ↔ | ↔ | ↔ | ↔ | ↔ | ↔ | ↔ | ↔ |
| Cabanas-Sanchez (2021) | Older adults | Depression | ↔ | ↔ | ↑ | ↔ | ↓ | ↑ | ↑ | ↑ | ↑ | ↓ | ↓ | ↓ |
| Cabanas-Sanchez (2021) | Older adults | Depression^#^ | ↑ | ↔ | ↔ | ↓ | ↔ | ↔ | ↔ | ↔ | ↔ | ↔ | ↔ | ↔ |
| Blodgett (2022) | Adults | Depression risk | ↔ | ↑ | ↑ | ↔ | ↑ | ↑ | ↓ | ↓ | ↑ | ↓ | ↓ | ↓ |
| Kandola (2021) | Adults | Depression score^#^ |  |  |  | ↑ | ↑ | ↑ |  |  |  |  |  |  |
| del Pozo Cruz (2020) | Adults | Depressive symptoms |  |  |  | ↑ | ↔ | ↑ |  |  |  |  |  |  |
| Su (2022) | Adults | Depressive symptoms | ↓ | ↔ | ↑ | ↑ | ↑ | ↑ | ↔ | ↓ | ↔ | ↓ | ↓ | ↓ |
| Hofman (2022) | Adults/Older adults | Depressive symptoms | ↔ | ↔ | ↑ | ↔ | ↔ | ↑ | ↔ | ↔ | ↔ | ↓ | ↓ | ↔ |
| Sampasa-Kanyinga (2021) | Children | Depressive symptoms^#^ |  |  | ↓ |  |  |  |  |  |  | ↑ |  |  |
| Brown (2021) | Young children | Externalising problems |  |  |  |  | ↔ | ↔ |  | ↔ | ↔ |  | ↔ | ↔ |
| Cabanas-Sanchez (2021) | Older adults | Global mental health | ↔ | ↔ | ↔ | ↔ | ↔ | ↔ | ↔ | ↔ | ↔ | ↔ | ↔ | ↔ |
| Cabanas-Sanchez (2021) | Older adults | Global mental health^#^ | ↔ | ↔ | ↑ | ↔ | ↔ | ↑ | ↔ | ↔ | ↑ | ↓ | ↓ | ↓ |
| Cabanas-Sanchez (2021) | Older adults | Happiness | ↔ | ↔ | ↑ | ↔ | ↔ | ↑ | ↔ | ↔ | ↑ | ↓ | ↓ | ↓ |
| Cabanas-Sanchez (2021) | Older adults | Happiness^#^ | ↔ | ↔ | ↔ | ↔ | ↔ | ↔ | ↔ | ↔ | ↔ | ↔ | ↔ | ↔ |
| Fairclough (2021) | Children/Adolescents | Internalising problems | ↓ |  |  | ↑ | ↔ | ↑ |  | ↔ |  |  | ↓ |  |
| Brown (2021) | Young children | Internalising problems |  |  |  |  | ↔ | ↔ |  | ↔ | ↔ |  | ↔ | ↔ |
| Kuzik (2020) | Young children | Internalising problems | ↔ |  | ↔ | ↔ | ↔ | ↔ |  | ↔ | ↔ | ↔ | ↔ | ↔ |
| Cabanas-Sanchez (2021) | Older adults | Loneliness | ↔ | ↔ | ↑ | ↔ | ↔ | ↑ | ↔ | ↔ | ↑ | ↓ | ↓ | ↓ |
| Cabanas-Sanchez (2021) | Older adults | Loneliness^#^ | ↔ | ↔ | ↔ | ↔ | ↔ | ↔ | ↔ | ↔ | ↔ | ↔ | ↔ | ↔ |
| Larisch (2020) | Adults | Mental wellbeing |  |  | ↔ |  |  | ↑ |  |  | ↑ | ↓ | ↓ | ↓ |
| Fairclough (2021) | Children | Prosocial behaviour | ↓ |  |  | ↑ | ↑ | ↑ |  | ↓ |  |  | ↓ |  |
| Ren (2022) | Adolescents | Smartphone addiction |  |  |  |  |  | ↑ |  |  |  |  |  |  |
| Kuzik (2020) | Young children | Sociability | ↔ |  | ↑ | ↔ | ↔ | ↑ |  | ↔ | ↔ | ↓ | ↓ | ↓ |
|  | **Cognitive health** |  |  |  |  |  |  |  |  |  |  |  |  |  |
| Kuzik (2020) | Young children | Cognitive self-regulation | ↔ |  | ↔ | ↔ | ↔ | ↔ |  | ↔ | ↑ | ↔ | ↔ | ↓ |
| Whitaker (2021) | Adults | DSST^#^ |  |  |  |  | ↔ | ↔ |  |  | ↔ |  |  |  |
| Bezerra (2020) | Young children | Executive function | ↓ | ↓ | ↑ | ↔ | ↔ | ↔ | ↔ | ↔ | ↑ | ↓ | ↔ | ↔ |
| Fairclough (2021) | Children | Inhibition errors |  | ↓ |  |  | ↓ |  | ↑ | ↑ | ↑ |  |  | ↓ |
| Whitaker (2021) | Adults | RAVLT^#^ |  |  |  |  | ↔ | ↔ |  |  | ↔ |  |  |  |
| Migueles (2020) | Children | Right hippocampal GMV |  |  |  |  | ↔ | ↔ |  | ↔ |  |  | ↔ |  |
| Whitaker (2021) | Adults | Stroop^#^ |  |  |  |  | ↔ | ↔ |  |  | ↔ |  |  |  |
| Fairclough (2021) | Children | Switching errors |  | ↓ |  |  | ↓ |  | ↑ | ↑ | ↑ |  |  | ↓ |
| Kuzik (2020) | Young children | Vocabulary | ↑ |  | ↔ | ↓ | ↓ | ↔ |  | ↔ | ↔ | ↔ | ↔ | ↔ |
|  | **Fitness** |  |  |  |  |  |  |  |  |  |  |  |  |  |
| Lemos (2021) | Young children | 20m shuttle run (laps) |  | ↔ | ↑ |  | ↔ | ↑ |  |  | ↔ |  |  | ↔ |
| Zhang (2022) | Adolescents | 50m run (s) | ↔ | ↓ | ↑ | ↔ | ↓ | ↑ | ↑ | ↑ | ↑ | ↓ | ↓ | ↓ |
| del Pozo-Cruz (2022) | Older adults | 6MWT |  |  |  |  | ↑ | ↑ |  |  |  |  |  |  |
| Germano-Soares (2021) | Older adults | 6MWT |  |  |  |  | ↔ | ↑ |  |  |  |  |  |  |
| Zhang (2022) | Adolescents | 800/1000 m running score | ↓ | ↓ | ↔ | ↑ | ↓ | ↑ | ↑ | ↑ | ↑ | ↓ | ↓ | ↓ |
| del Pozo-Cruz (2022) | Older adults | CST Number of cycles |  |  |  |  | ↑ | ↑ |  |  |  |  |  |  |
| del Pozo-Cruz (2022) | Older adults | Hand Grip (Kg) |  |  |  |  | ↔ | ↑ |  |  |  |  |  |  |
| Zhang (2022) | Adolescents | Long jump (m) | ↓ | ↓ | ↔ | ↑ | ↔ | ↑ | ↑ | ↔ | ↑ | ↓ | ↓ | ↓ |
| Lemos (2021) | Young children | Speed/agility (sec) |  | ↓ | ↔ |  | ↔ | ↔ |  |  | ↔ |  |  | ↔ |
| Lemos (2021) | Young children | Standing long jump (cm) |  | ↑ | ↔ |  | ↔ | ↔ |  |  | ↔ |  |  | ↔ |
| del Pozo-Cruz (2022) | Older adults | TUG test |  |  |  |  | ↑ | ↑ |  |  |  |  |  |  |
| Dumuid (2018) | Older adults | VO2max | ↔ | ↔ | ↑ | ↔ | ↔ | ↑ | ↔ | ↔ | ↑ | ↓ | ↓ | ↓ |
| Oviedo-Caro (2020) | Adults | VO2max | ↔ | ↑ | ↑ | ↔ | ↔ | ↑ | ↓ | ↔ | ↔ | ↓ | ↓ | ↓ |
|  | **General health** |  |  |  |  |  |  |  |  |  |  |  |  |  |
| Verhoog (2020) | Adults/Older adults | HRQoL | ↔ | ↔ | ↑ | ↔ | ↔ | ↑ | ↔ | ↔ | ↑ | ↓ | ↓ | ↓ |
| Curtis (2020) | Adults | Physical HRQoL | ↔ | ↔ | ↑ | ↔ | ↓ | ↑ | ↔ | ↑ | ↑ | ↓ | ↓ | ↓ |
| Verhoog (2020) | Adults/Older adults | VAS | ↔ | ↔ | ↑ | ↔ | ↔ | ↑ | ↔ | ↔ | ↑ | ↓ | ↓ | ↓ |
|  | **Mortality** |  |  |  |  |  |  |  |  |  |  |  |  |  |
| Clarke (2021) | Adults | Mortality^#^ | ↔ | ↑ | ↑ | ↔ | ↑ | ↑ | ↓ | ↓ | ↑ | ↓ | ↓ | ↓ |
| Von Rosen (2019) | Adults/Older adults | Mortality^#^ |  |  |  |  |  |  |  | ↔ |  |  | ↓ |  |
|  | **Motor skills** |  |  |  |  |  |  |  |  |  |  |  |  |  |
| Estevan (2022) | Children | Actual ball skills |  |  |  |  | ↓ | ↔ |  | ↑ | ↑ |  | ↔ | ↓ |
| Estevan (2022) | Children | Actual locomotion |  |  |  |  | ↓ | ↑ |  | ↑ | ↑ |  | ↓ | ↓ |
| Estevan (2022) | Children | Actual MC |  |  |  |  | ↓ | ↑ |  | ↑ | ↑ |  | ↓ | ↓ |
| Smith (2020) | Children | FMS | ↓ | ↔ | ↔ | ↑ | ↔ | ↑ | ↔ | ↔ | ↑ | ↔ | ↓ | ↔ |
| Kuzik (2020) | Young children | Locomotor MS | ↔ |  | ↑ | ↔ | ↓ | ↔ |  | ↑ | ↑ | ↓ | ↔ | ↓ |
| Mota (2020) | Young children | Locomotor MS | ↔ | ↔ | ↔ | ↔ | ↔ | ↑ | ↔ | ↔ | ↔ | ↔ | ↔ | ↔ |
| Kuzik (2020) | Young children | Object control MS | ↔ |  | ↑ | ↔ | ↔ | ↑ |  | ↔ | ↑ | ↓ | ↓ | ↓ |
| Mota (2020) | Young children | Object control MS | ↑ | ↓ | ↔ | ↓ | ↓ | ↔ | ↑ | ↑ | ↑ | ↔ | ↓ | ↓ |
| Kuzik (2020) | Young children | Total MS | ↔ |  | ↑ | ↔ | ↓ | ↑ |  | ↑ | ↑ | ↓ | ↓ | ↓ |
| Mota (2020) | Young children | Total MS | ↑ | ↓ | ↔ | ↓ | ↓ | ↔ | ↑ | ↑ | ↑ | ↔ | ↔ | ↓ |
|  | **Other** |  |  |  |  |  |  |  |  |  |  |  |  |  |
| del Pozo-Cruz (2022) | Older adults | Barthel Index |  |  |  |  | ↑ | ↑ |  |  |  |  |  |  |
| Bianchim (2022) | Children | Lung capacity (FEV1% predicted) | ↓ | ↓ | ↓ | ↑ | ↑ | ↑ | ↑ | ↓ | ↔ | ↑ | ↓ | ↔ |
| Bianchim (2022) | Adults | Lung capacity (FEV1% predicted) | ↔ | ↑ | ↔ | ↓ | ↑ | ↑ | ↓ | ↓ | ↓ | ↓ | ↓ | ↑ |
|  | **Chronic disease** |  |  |  |  |  |  |  |  |  |  |  |  |  |
| Walmsley (2021) | Adults | Incident CVD^#^ | ↓ | ↔ | ↑ | ↑ | ↑ | ↑ | ↔ | ↓ | ↑ | ↓ | ↓ | ↓ |
| Tsunoda (2021) | Adults | NAFLD |  |  |  |  | ↔ | ↑ |  | ↔ | ↑ |  | ↓ | ↓ |

↑, reallocation that was favourable for health

↔, reallocation that was not significantly related to health

↓, reallocation that was unfavourable for health

#, indicates longitudinal results

Abbreviations: BMI, body mass index; BP, blood pressure; CMR, cardio-metabolic risk; CRP, C-reactive protein; CST, chair stand cycles; CVD, cardio-vascular disease; DSST, digit symbol substitution test; FMI, fat mass index; FFMI, fat-free mass index; FEV1%, forced expiratory volume in 1 second; HbAlc, hemoglobin A1c; HDL, high-density lipoprotein; HOMA-IS, Homeostasis Model Assessment of Insulin Sensitivity; HRQoL, health-related quality of life; IGFBP, insulin-like growth factor-binding protein; LPA, light physical activity; Matsuda-ISI, matsuda insulin sensitivity index; MC, motor competence; MetS, metabolic syndrome score; MS, motor skills; MVPA, moderate-to-vigorous physical activity; NAFLD, non-alcoholic fatty liver disease; RAVLT, rey auditory verbal learning test; SB, sedentary behaviour; SD, standard deviation; SDQ, strength and difficulties questionnaire; TUG, timed up and go; VAS, visual analogue scale of happiness; VPA, vigorous physical activity; WC, waist circumference; zBMI, body mass index z-score; %BF, body fat percentage; %WHtR, waist-to-height ratio
