# Supplementary material for: The association between reallocations of time and health using compositional data analysis: a systematic scoping review with an interactive data exploration interface
Source: Int J Behav Nutr Phys Act. 2023 Oct 19;20:127. doi: 10.1186/s12966-023-01526-x (PMC10588100; doi:10.1186/s12966-023-01526-x)
Supplement: Supplementary file 7 — Supplementary Material 7: Table S6. Summary of results for studies presenting reallocations between sleep, sedentary behaviour and physical activity [file 12966_2023_1526_MOESM7_ESM.docx]

Table S6. Summary of results for studies presenting reallocations between sleep, sedentary behaviour and physical activity

| Study | Age group | Health outcome | Duration of reallocation (min) | LPA to Sleep | LPA to SB | LPA to MVPA | MVPA to sleep | MVPA to SB | MVPA to LPA | SB to sleep | SB to LPA | SB to MVPA | Sleep to SB | Sleep to SB | Sleep to MVPA |
| --- | --- | --- | --- | --- | --- | --- | --- | --- | --- | --- | --- | --- | --- | --- | --- |
| **Adiposity** |  |  |  |  |  |  |  |  |  |  |  |  |  |  |  |
| Dumuid (2018) | Children | % Fat-free mass | 15.00 | 0.4 * | 0.2 * | 1.2 * | -1.2 * | -1.4 * | -1.6 * | 0.2 | -0.2 * | 1 * | -0.2 * | -0.4 * | 0.8 * |
| Dumuid (2018) | Children | % Nontruncal fat | 15.00 | -0.1 * | 0 | -0.4 * | 0.4 * | 0.4 * | 0.5 * | -0.1 * | 0 | -0.3 * | 0.1 * | 0.1 * | -0.3 * |
| Dumuid (2018) | Children | % Truncal fat | 15.00 | -0.3 * | -0.2 * | -0.8 * | 0.8 * | 0.9 * | 1.1 * | -0.1 | 0.2 * | -0.7 * | 0.1 | 0.3 * | -0.5 * |
| Dumuid (2018) | Children (girls) | %BF | 30.00 | -0.49 * | 0.02 | -1.74 * | 2.44 * | 2.94 * | 2.9 * | -0.52 * | -0.06 | -1.78 * | 0.52 * | 0.47 * | -1.24 * |
|  | Children (boys) | %BF | 30.00 | -0.53 * | -0.33 * | -1.86 * | 2.11 * | 2.31 * | 2.6 * | -0.2 | 0.28 | -1.54 * | 0.2 | 0.49 * | -1.33 * |
| Gaba (2020) | Children | %BF^%^ | 17.14 |  | 0.3 |  |  | 3.6 |  |  | -0.3 | -2.8 |  |  |  |
| Pelclova (2020) | Older adults | %BF | 30.00 |  | 0.27 * | -0.37 |  | 1.28 * | 1.02 * |  | -0.27 * | -0.65 * |  |  |  |
| Pelclova (2018) | Older adults | %BF | 30.00 |  |  |  |  |  |  |  | -0.35 | -2.18 |  |  |  |
| Powell (2020) | Older adults | %BF | 30.00 | 2.1 * | 2.13 * | 1.72 * | 0.85 | 0.87 | -0.73 | -0.03 | -1.61 * | -0.42 | 0.02 | -1.58 * | -0.39 |
| Rubin (2022) | Children/Adolescents | %BF^%^ | 17.14 |  |  |  |  |  |  |  | -0.2 |  |  |  |  |
| Swindell (2020) | Adults | %BF^%^ | 10.00 | 0.06 | 0.14 * | -0.76 * | 1.3 * | 1.38 * | 1.23 * | -0.08 | -0.14 * | -0.91 * | 0.08 | -0.06 | -0.83 * |
| Fairclough (2017) | Children (whole sample) | %WHtR | 15.00 | -0.4 | -0.5 | -3.1 | 5.1 | 5 | 5.5 | 0.1 | 0.4 | -2.7 | -0.1 | 0.3 | -2.8 |
|  | Children (Underweight) | %WHtR | 15.00 | -0.3 | -0.4 | -2.5 | 3.4 | 3.3 | 3.7 | 0.1 | 0.4 | -2.1 | -0.1 | 0.3 | -2.2 |
|  | Children (Normal weight) | %WHtR | 15.00 | -0.4 | -0.5 | -2.9 | 4.5 | 4.4 | 4.8 | 0.1 | 0.4 | -2.5 | -0.1 | 0.3 | -2.6 |
|  | Children (Overweight/obese) | %WHtR | 15.00 | -0.4 | -0.5 | -4.1 | 10.8 | 10.7 | 11.1 | 0.1 | 0.5 | -3.6 | -0.1 | 0.4 | -3.7 |
| Biddle (2021) | Adults | BMI | 60.00 |  |  |  |  |  |  |  | -0.05 * | -0.33 * |  |  |  |
| Chastin (2015) | Adults | BMI^%^ | 10.00 | -0.002 | 0 | -0.001 | -0.07 | 1.21 | 0.85 | -0.002 | 0 | -0.001 | 0.003 | 0.003 | 0 |
| Dumuid (2018) | Older adults | BMI | 15.00 | 0 | -0.1 | -0.7 * | 1.3 * | 1.2 * | 1.3 * | 0.1 | 0.1 | -0.7 * | -0.1 | 0 | -0.8 * |
| Healy (2020) | Children | BMI | 30.00 | -0.471 * | -0.079 | 0.032 | -0.658 | -0.267 | -0.2 | -0.396 | 0.063 | 0.106 | 0.415 | 0.482 * | 0.526 |
| Kim (2021) | Adults (accelerometer data) | BMI | 30.00 |  | 0.46 |  |  | 0.84 |  | -0.53 | -0.4 | -0.35 | 0.57 |  |  |
| Kim (2021) | Adults (recall data) | BMI | 30.00 |  |  |  |  | 0.21 |  | -0.27 |  | -0.14 | 0.28 |  |  |
| Oviedo-Caro (2020) | Adults | BMI^%^ | 30.00 | 0.28 | 0.57 * | -0.19 | 0.73 * | 1.02 * | 0.47 | -0.29 | -0.54 * | -0.76 * | 0.29 | -0.25 | -0.47 |
| Pelclova (2020) | Older adults | BMI | 30.00 |  | 0.1 * | -0.26 * |  | 0.75 * | 0.65 * |  | -0.1 * | -0.37 * |  |  |  |
| Pelclova (2018) | Older adults | BMI | 30.00 |  |  |  |  |  |  |  | -0.14 | -1.51 |  |  |  |
| Powell (2020) | Older adults | BMI | 30.00 | 0.91 * | 1.22 * | 0.88 * | 0.29 | 0.61 | -0.34 | -0.32 * | -0.96 * | -0.35 | 0.32 * | -0.63 * | -0.02 |
| Swindell (2020) | Adults | BMI^%^ | 10.00 | -0.05 | 0.11 * | -0.78 * | 1.17 * | 1.13 * | 1.22 * | -0.16 * | -0.11 * | -0.89 * | 0.16 * | 0.05 | -0.73 * |
| Powell (2020) | Older adults | Fat mass | 30.00 | 2.59 * | 1.28 | 2.15 * | 1.09 | 2.78 * | -0.83 | -0.21 | -2.12 * | -0.65 | 0.19 | -1.92 * | -0.44 |
| Gaba (2021) | Older adults | FFMI^%^ | 30.00 |  |  |  |  |  |  |  | 0.1 | -0.13 |  |  |  |
| Gaba (2020) | Children | FMI^%^ | 17.14 |  | 0.2 |  |  | 5.6 |  |  | -0.2 | -4.2 |  |  |  |
| Gaba (2021) | Older adults | FMI^%^ | 30.00 |  |  |  |  |  |  |  | -0.71 | 1.14 |  |  |  |
| Oviedo-Caro (2020) | Adults | FMI^%^ | 30.00 | 0.31 * | 0.46 * | -0.12 | 0.65 * | 0.8 * | 0.35 | -0.15 | -0.42 * | -0.56 * | 0.14 | -0.28 * | -0.42 * |
| Rubin (2022) | Children/Adolescents | FMI^%^ | 17.14 |  |  |  |  |  |  |  | 0 |  |  |  |  |
| Sandborg (2022) | Adults | FMI | 30.00 |  |  |  |  |  |  |  | -0.08 * |  |  |  |  |
| Rossen (2019) | Adults/Older adults | Sagittal abdominal diameter ^%^ | 30.00 |  | 1 | -4.2 |  |  |  |  | -1 | -5.3 |  |  |  |
|  |  |  | 19.00 |  |  |  |  | 17.7 | 16.5 |  |  |  |  |  |  |
| Oviedo-Caro (2020) | Adults | Sum of skinfolds^%^ | 30.00 | 1.36 * | 1.58 * | -0.34 | 2.42 * | 2.65 * | 1.13 | -0.25 | -1.46 * | -1.88 * | 0.2 | -1.24 * | -1.66 * |
| Gaba (2020) | Children | Visceral adipose tissue^%^ | 17.14 |  | -0.3 |  |  | 17.7 * |  |  | 0.3 | -11.9 * |  |  |  |
| Gaba (2021) | Older adults | Visceral adipose tissue^%^ | 30.00 |  |  |  |  |  |  |  | -0.09 | 0.14 |  |  |  |
| Rubin (2022) | Children/Adolescents | Visceral adipose tissue^%^ | 17.14 |  |  |  |  |  |  |  | -2.5 |  |  |  |  |
| Dumuid (2018) | Older adults | W:Hip | 15.00 | -0.2 | 0 | -1.3 * | 2 * | 2.1 * | 2.2 * | -0.2 | 0 | -1.2 * | 0.2 | 0.2 | -1.2 * |
| Biddle (2021) | Adults | WC | 60.00 |  |  |  |  |  |  |  | -0.07 * | -0.3 * |  |  |  |
| Carson (2016) | Children/Adolescents | WC^%^ | 10.00 | -0.005 | 0.022 | -0.001 | 0.004 | 0.07 | 0.008 | -0.001 | -0.001 | -0.001 | 0.001 | 0 | 0 |
| Chastin (2015) | Adults | WC^%^ | 10.00 | -0.001 | 0.001 | -0.001 | 0.29 | 0.84 | 0.55 | -0.001 | 0 | -0.001 | 0.001 | 0.001 | -0.001 |
| Lee (2020) | Older adults | WC | 5.00 |  |  | -1.94 | 4.65 | 4.78 | 4.81 |  |  | -1.9 |  |  | -1.78 |
|  |  |  | 30.00 | -0.99 | -0.26 |  |  |  |  | -0.74 | 0.2 |  | 0.77 | 0.99 |  |
| Swindell (2020) | Adults | WC^%^ | 10.00 | -0.02 | 0.08 * | -0.82 * | 1.28 * | 1.38 * | 1.29 * | -0.1 * | -0.09 * | -0.91 * | 0.1 * | 0.02 | -0.81 * |
| Carson (2016) | Children/Adolescents | zBMI^%^ | 10.00 | -0.571 | 1.129 | -0.119 | 1.088 | 5.072 | 1.23 | -0.053 | -0.026 | -0.036 | 0.048 | 0.011 | -0.007 |
| Curtis (2020) | Adults | zBMI | 15.00 | -0.02 | -0.01 | -0.06 * | 0.05 * | 0.06 * | 0.07 * | -0.01 | 0.01 | -0.05 * | 0.01 | 0.02 | -0.04 * |
| Domingues (2022) | Adolescents | zBMI | 120.00 | -0.11 | -0.16 | 0.31 |  |  |  | 0.05 | 0.11 | 0.48 | -0.04 | 0.06 | 0.43 |
| Fairclough (2017) | Children (total sample) | zBMI | 15.00 | -0.01 | -0.06 | -0.49 | 0.88 | 0.83 | 0.89 | 0.05 | 0.06 | -0.43 | -0.05 | 0.01 | -0.48 |
|  | Underweight |  | 15.00 | -0.01 | -0.06 | -0.4 | 0.59 | 0.54 | 0.6 | 0.05 | 0.06 | -0.33 | -0.05 | 0.01 | -0.39 |
|  | Normal weight |  | 15.00 | -0.01 | -0.06 | -0.46 | 0.77 | 0.72 | 0.78 | 0.05 | 0.06 | -0.4 | -0.05 | 0.01 | -0.45 |
|  | Overweight/obese |  | 15.00 | -0.01 | -0.06 | -0.65 | 1.82 | 1.77 | 1.83 | 0.05 | 0.06 | -0.59 | -0.05 | 0.01 | -0.64 |
| Haszard (2020) | Children | zBMI | 32.00 | -0.22 * | -0.13 * | -0.41 * |  |  |  |  |  |  |  |  |  |
|  |  |  | 10.00 |  |  |  | 0.09 * | 0.11 * | 0.15 * |  |  |  |  |  |  |
|  |  |  | 46.00 |  |  |  |  |  |  | -0.13 * | 0.15 * | -0.38 * |  |  |  |
|  |  |  | 59.00 |  |  |  |  |  |  |  |  |  | 0.17 * | 0.38 * | -0.29 * |
| Kuzik (2020) | Young children | zBMI | 30.00 |  |  |  |  | -0.23 * |  |  |  | 0.19 * |  |  |  |
| Fariclough (2022) | Adolescents | Using different accelerometer intensity bands, reallocating time to higher intensity bands most strongly associated with reduced adiposity. Suggesting that reallocating to higher intensity VPA is more strongly associated with reduced adiposity than lower intensity MPA. | | | | | | | | | | | | | |
| Johansson (2020) | Adults/older adults | Reallocating time from SB to high-intensity PA (stair climbing, running, cycling) associated with lower WC, but reallocating SB to walking was not. | | | | | | | | | | | | | |
| Migueles (2022) | Young children | Reallocating time to VPA from other behaviours (SB, LPA, MPA) individually or collectively associated with favourable changes to BMI and FFMI. Results suggest that behaviour/s reallocated from were irrelevant, as long as VPA was increased. | | | | | | | | | | | | | |
| Ng (2021) | Children | Reallocating time to MVPA from other behaviours collectively 2-6 times more potent than reallocations to sleep or away from SB (from other behaviours collectively) in terms of lower adiposity. Reallocating time to LPA unfavourable for all outcomes. | | | | | | | | | | | | | |
| Rees-Punia (2021) | Adults | Reallocating SB to MVPA associated with modest weight loss in Latinx participants. Reallocating SB to sleep associated with modest weight gain in white participants. | | | | | | | | | | | | | |
| Rubin (2022) | Children/adolescents | Reallocating time from SB to VPA, but not LPA or MPA associated with favourable changes to adiposity. For example, reallocating 15min/week of SB to VPA associated with 3.3% reduction in visceral adipose tissue. Favourable, but non-significant, findings also found for FMI and FFMI. | | | | | | | | | | | | | |
| Talarico (2018) | Children | Reallocating time to MVPA from other behaviours collectively associated with strongest favourable association with adiposity markers (BMI, WC, FMI). Reallocating time to LPA (from other behaviours collectively) showed unfavourable associations with adiposity, however, were weaker than reallocations involving MVPA. No association found for reallocations involving sleep and SB. | | | | | | | | | | | | | |
| Taylor (2020) | Children | When reallocating time equivalent to a 10% increase in each behaviour increasing sleep showed strongest favourable association with odds of being obese. However, 10% increase in sleep (57min/day) was a much larger increase than 6.9min/day for MVPA. | | | | | | | | | | | | | |
| Taylor (2018) | Young children | Reallocating time to increase sleep by 10% at expense of others collectively, associated with favourable changes to zBMI at some timepoints, but not all. Reallocating time to increase LPA or SB by 10% at expense of others collectively, associated with unfavourable changes to zBMI at some timepoints, but not all. Reallocations involving MVPA not significant, but durations very small (<2min/day). | | | | | | | | | | | | | |
| Winkler (2018) | Adults | Reallocating time from sitting to standing or stepping associated with favourable changes to adiposity (%BF), however, benefits much larger (3-5 times) when reallocating to stepping than standing. However, using different ratios of standing/stepping showed even if only a small amount of time was reallocated to stepping, the favourable associations were much stronger. | | | | | | | | | | | | | |
| Matricciani (2020) | Children | Reallocating time to MVPA from any other behaviour (sleep, SB, LPA) showed favourable association for adiposity. Reallocating time from MVPA to any other behaviour (sleep, SB, LPA) showed unfavourable association for adiposity. Reallocating time from SB or LPA to sleep also showed favourable associations with adiposity, albeit much weaker than reallocations involving MVPA. | | | | | | | | | | | | | |
| Matricciani (2020) | Adults | Reallocating time to MVPA from any other behaviour (sleep, SB, LPA) showed favourable association for adiposity. Reallocating time from MVPA to any other behaviour (sleep, SB, LPA) showed unfavourable association for adiposity. Reallocating time from SB or sleep to LPA also showed favourable associations with adiposity, albeit much weaker than reallocations involving MVPA. | | | | | | | | | | | | | |
| Farrahi (2021) | Adults | Reallocating time to MVPA from any behaviour associated with favourable changes in multiple markers of adiposity (fat mass, %BF, WC). Reallocating time away from MVPA unfavourable for adiposity. Reallocations from SB or sleep to LPA also favourable, albeit relationship weaker. | | | | | | | | | | | | | |
| Biomarkers |  |  |  |  |  |  |  |  |  |  |  |  |  |  |  |
| Biddle (2018) | Adults | 2-h glucose | 30.00 | 1 | 0.99 | 0.95 * | 1.06 * | 1.06 * | 1.07 * | 1.01 | 1.01 | 0.96 * | 0.99 | 1 | 0.95 * |
| Brakenridge (2021) | Adults (low risk diabetes) | 2-h glucose^%^ | 60.00 | 0.7 | -0.2 | -4 | 7.8 * | 6.9 * | 7 | 0.9 | 0.1 | -3.8 * | -1 | -0.8 | -4.8 |
|  | Adults (high risk diabetes) | 2-h glucose^%^ | 60.00 | 2.7 | 1.2 | -3.8 | 10.8 * | 9.1 * | 8.1 | 1.5 | -1 | -5 * | -1.7 | -2.6 | -6.5 * |
| Biddle (2018) | Adults | 2-h insulin | 30.00 | 1.02 | 1 | 0.86 * | 1.21 * | 1.19 * | 1.19 * | 1.02 | 1 | 0.86 * | 0.98 | 0.98 | 0.84 * |
| Farrahi (2021) | Adults | 2-h insulin^%^ | 30.00 |  |  | -9.4 * | 31.2 * | 30.3 * | 26.3 * |  |  | -12.4 * |  |  | -13 * |
|  |  |  | 60.00 | 8.3 * | 6.9 * |  |  |  |  |  | -6.1 * |  |  | -7.4 * |  |
| Rossen (2019) | Adults/Older adults | Adiponectin^%^ | 30.00 |  | -2.8 | -8.5 |  |  |  |  | 2.6 | -5.8 |  |  |  |
|  |  |  | 19.00 |  |  |  |  | 25.3 | 27.3 |  |  |  |  |  |  |
| Marshall (2021) | Children (non-diabetic) | Arterial stiffness (pulse wave velocity) ^%^ | 20.00 | 0.07 | -0.46 | -2.48 | 3.21 | 2.68 | 3.13 | 0.55 | 0.46 | -2.01 | -0.55 | -0.1 | -2.57 |
| Marshall (2021) | Children (diabetic) | Arterial stiffness (pulse wave velocity) ^%^ | 20.00 | 0.1 | -0.4 | -3.29 | 5.07 | 4.58 | 4.97 | 0.5 | 0.4 | -2.89 | -0.51 | -0.12 | -3.4 |
| Marshall (2022) | Children (boys) | Arterial stiffness (pulse wave velocity) ^%^ | 20.00 | 0.36 | 0.09 | -0.25 | 1.05 | 0.77 | 0.7 | 0.32 | -0.07 | -0.33 | -0.28 | -0.35 | -0.62 |
| Marshall (2022) | Children (girls) | Arterial stiffness (pulse wave velocity) ^%^ | 20.00 | 0.34 | 0.07 | -0.31 | 1.22 | 0.95 | 0.89 | 0.27 | -0.05 | -0.37 | -0.28 | -0.34 | -0.66 |
| Rossen (2019) | Adults/Older adults | C-peptide^%^ | 30.00 |  | 1.3 | -1.3 |  |  |  |  | -1.2 | -2.7 |  |  |  |
|  |  |  | 19.00 |  |  |  |  | 8.1 | 7.3 |  |  |  |  |  |  |
| Biddle (2021) | Adults | CMR Risk Score | 60.00 |  |  |  |  |  |  |  | -0.03 * | -0.13 * |  |  |  |
| Carson (2016) | Children/Adolescents | CRP^%^ | 10.00 | -0.985 | -0.369 | -0.002 | -2.423 | -0.885 | 0.019 | -0.018 | 0.008 | 0.006 | 0.018 | 0.021 | 0.015 |
| Carson (2016) | Children/Adolescents | Diastolic BP^%^ | 10.00 | 0.001 | 0.006 | 0 | 0.005 | 0.017 | 0.001 | 0 | 0 | 0 | 0 | 0 | 0 |
| Chastin (2015) | Adults | Diastolic BP^%^ | 10.00 | -0.001 | 0 | 0 | -0.36 | 0.07 | -0.004 | 0 | 0 | 0 | 0.001 | 0.001 | 0.001 |
| Lee (2020) | Older adults | Diastolic BP | 5.00 |  |  | 0.35 | -0.71 | -0.62 | -0.76 |  |  | 0.21 |  |  | 0.3 |
|  |  |  | 30.00 | 0.35 | 0.85 |  |  |  |  | -0.52 | -0.81 |  | 0.52 | -0.27 |  |
| Sandborg (2022) | Adults | Diastolic BP | 10.00 |  |  |  |  |  |  |  |  | 0.24 * |  |  |  |
| Biddle (2018) | Adults | Fasting glucose | 30.00 | 1 | 1 | 0.99 | 1 | 1.01 | 1.02 | 1 | 1 | 0.99 | 1 | 1 | 0.99 |
| Brakenridge (2021) | Adults (low risk diabetes) | Fasting glucose^%^ | 60.00 | -1.1 | -0.1 | -0.7 | -0.3 | 0.7 | 0.7 | -1.1 * | 0 | -0.6 | 1.1 * | 1.1 * | 0.5 |
| Brakenridge (2021) | Adults (high risk diabetes) | Fasting glucose^%^ | 60.00 | 2.1 * | 1.7 * | 0.4 | 2.5 | 2.1 | 0.7 | 0.3 | -1.5 * | -1.4 | -0.4 | -1.8 * | -1.7 |
| Lee (2020) | Older adults | Fasting glucose | 5.00 |  |  | -0.67 | 1.84 | 1.83 | 1.78 |  |  | -0.73 |  |  | -0.74 |
|  |  |  | 30 | 0.39 | 0.32 |  |  |  |  | 0.06 | -0.2 |  | -0.07 | -0.3 |  |
| Powell (2020) | Older adults | Fasting glucose | 30.00 | -0.07 | -0.09 | -0.1 | 0.08 | 0.05 | 0.12 | 0.03 | 0.07 | -0.01 | -0.03 | 0.05 | -0.04 |
| Biddle (2018) | Adults | Fasting insulin | 30.00 | 1.02 | 1.01 | 0.97 | 1.07 | 1.06 | 1.05 | 1.01 | 0.99 | 0.96 | 0.99 | 0.98 | 0.95 |
| Carson (2016) | Children/Adolescents | Fasting insulin^%^ | 10.00 | 0.015 | 0.067 | -0.002 | 0.084 | 0.213 | 0.024 | -0.002 | -0.001 | -0.001 | 0.001 | 0 | -0.001 |
| Biddle (2021) | Adults | HbA1c | 60.00 |  |  |  |  |  |  |  | 0.02 | 0 |  |  |  |
| Rossen (2019) | Adults/Older adults | HbAlc^%^ | 30.00 |  | 0.7 | -1.1 |  |  |  |  | -0.6 | -1.8 |  |  |  |
|  |  |  | 19.00 |  |  |  |  | 5.9 | 5.4 |  |  |  |  |  |  |
| Biddle (2021) | Adults | HDL cholesterol | 60.00 |  |  |  |  |  |  |  | 0.05 * | 0.17 * |  |  |  |
| Carson (2016) | Children/Adolescents | HDL cholesterol^%^ | 10.00 | -0.042 | -0.074 | -0.004 | -0.015 | -0.093 | 0.047 | 0.001 | 0.002 | 0.001 | -0.001 | 0.001 | 0 |
| Lee (2020) | Older adults | HDL cholesterol | 5.00 |  |  | 1.72 | -4.43 | -4.37 | -4.37 |  |  | 1.71 |  |  | 1.78 |
|  |  |  | 30.00 | -0.31 | 0.03 |  |  |  |  | -0.34 | -0.03 |  | 0.36 | 0.33 |  |
| Powell (2020) | Older adults | HDL cholesterol | 30.00 | 0.08 | 0.06 | 0.1 | -0.08 | -0.1 | -0.14 | 0.02 | -0.04 | 0.04 | -0.02 | -0.06 | 0.02 |
| Rossen (2019) | Adults/Older adults | HDL cholesterol^%^ | 30.00 |  | -1.7 | -0.4 |  |  |  |  | 1.6 | 1.3 |  |  |  |
|  |  |  | 19.00 |  |  |  |  | -3.3 | -2.3 |  |  |  |  |  |  |
| Marshall (2021) | Children (non-diabetic) | Heart rate variability (HF) | 20.00 | -3.61 | 0.08 | 5.5 | -11.01 | -7.32 | -7.43 | -3.73 | -0.16 | 5.38 | 3.8 | 3.69 | 9.23 |
| Marshall (2021) | Children (diabetic) | Heart rate variability (HF) | 20.00 | -4.64 | 0.19 | 11.52 | -23.02 | -18.19 | -18.42 | -4.89 | -0.29 | 11.27 | 4.99 | 4.76 | 16.32 |
| Marshall (2021) | Children (non-diabetic) | Heart rate variability (LF) | 20.00 | 1.83 | -0.03 | -2.76 | 5.54 | 3.68 | 3.73 | 1.88 | 0.07 | -2.71 | -1.92 | -1.87 | -4.65 |
| Marshall (2021) | Children (diabetic) | Heart rate variability (LF) | 20.00 | 1.64 | -0.06 | -4.04 | 8.08 | 6.38 | 6.46 | 1.72 | 0.1 | -3.95 | -1.76 | -1.68 | -5.73 |
| Rossen (2019) | Adults/Older adults | HOMA-IR^%^ | 30.00 |  | 4.4 | -6.1 |  |  |  |  | -3.9 | -10.1 |  |  |  |
|  |  |  | 19.00 |  |  |  |  | 39.2 | 35.7 |  |  |  |  |  |  |
| Biddle (2018) | Adults | HOMA-IS | 30.00 | 0.98 | 1 | 1.05 | 0.92 * | 0.93 | 0.93 | 0.99 | 1 | 1.05 | 1.02 | 1.02 | 1.07 * |
| Booker (2022) | Adults | hs-CRP concentration | 15.00 |  |  |  |  |  |  | 0 |  |  | 0 |  |  |
| Rossen (2019) | Adults/Older adults | IGFBP-1^%^ | 30.00 |  | 3 | 1.3 |  |  |  |  | -2.8 | -1.8 |  |  |  |
|  |  |  | 19.00 |  |  |  |  | 3.8 | 2 |  |  |  |  |  |  |
| Biddle (2021) | Adults | LDL cholesterol | 60.00 |  |  |  |  |  |  |  | -0.01 | 0.03 |  |  |  |
| Chastin (2015) | Adults | LDL cholesterol^%^ | 10.00 | 0.002 | 0.001 | 0 | 0.64 | 0 | -0.19 | 0.001 | 0 | -0.001 | -0.002 | -0.002 | -0.001 |
| Powell (2020) | Older adults | LDL cholesterol | 30.00 | 0.02 | 0.01 | 0 | 0.05 | 0.03 | 0.03 | 0.02 | 0 | -0.01 | -0.02 | -0.02 | -0.02 |
| Swindell (2020) | Adults | Log10 hs-CRP^%^ | 10.00 | 0.25 | 0.41 | -2.28 * | 3.93 * | 4.1 * | 3.69 * | -0.17 | -0.41 | -2.7 * | 0.17 | -0.24 | -2.53 * |
| Biddle (2018) | Adults | Matsuda-ISI | 30.00 | 0.98 | 1 | 1.09 * | 0.85 * | 0.87 * | 0.87 * | 0.98 | 1 | 1.1 * | 1.02 | 1.02 | 1.12 * |
| Sandborg (2022) | Adults (cross-sectional) | MetS score | 10.00 |  |  |  |  | 0.09 * |  |  |  |  |  |  |  |
| Sandborg (2022) | Adults (prospective) | MetS score | 30.00 |  |  |  |  |  |  |  | -0.05 |  |  |  |  |
| Lee (2020) | Older adults | MetS z-score | 5.00 |  |  | -0.41 | 1.07 | 1.07 | 1.06 |  |  | -0.43 |  |  | -0.42 |
|  | Older adults | MetS z-score | 30.00 | 0.05 | 0.11 |  |  |  |  | -0.05 | -0.1 |  | 0.05 | -0.05 |  |
| Swindell (2020) | Adults | sqrt HOMA-IR^%^ | 10.00 | 0.28 * | 0.32 * | -0.24 | 0.78 | 0.82 | 0.51 | 0.04 | -0.32 * | -0.56 | 0.04 | -0.28 * | -0.52 |
| Swindell (2020) | Adults | sqrt Insulin^%^ | 10.00 | 0.29 * | 0.28 * | -0.35 | 0.96 * | 0.95 * | 0.68 | 0.02 | -0.27 * | -0.63 * | -0.02 | -0.29 * | -0.65 * |
| Swindell (2020) | Adults | sqrt Triglycerides^%^ | 10.00 | 0.01 | 0.07 | -0.57 * | 0.9 * | 0.97 * | 0.9 * | -0.06 | -0.07 | -0.64 * | 0.06 | 0.01 | -0.57 * |
| Carson (2016) | Children/Adolescents | Systolic BP^%^ | 10.00 | -0.002 | 0.005 | -0.001 | 0.006 | -0.022 | 0.005 | 0 | 0 | 0 | 0 | 0 | 0 |
| Chastin (2015) | Adults | Systolic BP^%^ | 10.00 | -0.001 | -0.001 | 0 | -0.09 | -0.001 | 0.12 | 0 | 0 | 0 | 0 | 0.001 | 0 |
| Gupta (2018) | Adults | Systolic BP | 120.00 |  |  |  |  |  |  |  |  |  | 2 * |  |  |
| Lee (2020) | Older adults | Systolic BP | 5.00 |  |  | -0.67 | 1.46 | 1.51 | 1.21 |  |  | -0.73 |  |  | -0.59 |
|  |  |  | 30.00 | 1.55 | 1.88 |  |  |  |  | -0.34 | -1.76 |  | 0.32 | -1.42 |  |
| Sandborg (2022) | Adults | Systolic BP | 10.00 |  |  |  |  |  |  |  |  | 0.4 * |  |  |  |
| Biddle (2021) | Adults | Total cholesterol | 60.00 |  |  |  |  |  |  |  | -0.01 | 0.03 |  |  |  |
| Powell (2020) | Older adults | Total cholesterol | 30.00 | 0.1 | 0.06 | 0.08 | 0.02 | -0.02 | -0.06 | 0.04 | -0.04 | 0.02 | -0.04 | -0.08 | 0.06 |
| Biddle (2021) | Adults | Triglycerides | 60.00 |  |  |  |  |  |  |  | -0.06 * | -0.17 * |  |  |  |
| Carson (2016) | Children/Adolescents | Triglycerides^%^ | 10.00 | -0.11 | -0.068 | 0.088 | -2.172 | -2.064 | -0.975 | -0.001 | 0.001 | 0.013 | 0.001 | 0.002 | 0.014 |
| Lee (2020) | Older adults | Triglycerides | 5.00 |  |  | -4.95 | 12.78 | 12.71 | 12.65 |  |  | -5.01 |  |  | -5.08 |
|  |  |  | 30.00 | 0.76 | 0.38 |  |  |  |  | 0.37 | -0.36 |  | -0.2 | -0.39 |  |
| Powell (2020) | Older adults | Triglycerides | 30.00 | -0.06 | -0.06 | -0.08 | 0.04 | 0.05 | 0.08 | -0.01 | 0.04 | -0.02 | 0.01 | 0.04 | -0.02 |
| Rossen (2019) | Adults/Older adults | Triglycerides^%^ | 30.00 |  | 0.6 | -1.3 |  |  |  |  | -0.6 | -2 |  |  |  |
|  |  |  | 19.00 |  |  |  |  | 6.5 | 6.1 |  |  |  |  |  |  |
| Johansson (2020) | Adults/Older adults | Reallocating time from SB to high-intensity PA (stair climbing, running, cycling) associated with lower systolic BP and LDL cholesterol for adults and older adults. Reallocating SB to walking was only associated with lower systolic BP in older adults, with other outcomes not significant. Reallocations for SB to walking were smaller in magnitude than those from SB to high-intensity PA. | | | | | | | | | | | | | |
| Winkler (2018) | Adults | Reallocating time from sitting to standing or stepping associated with favourable changes to various biomarkers (systolic BP, triglycerides, insulin), however, benefits much larger (2-3 times) when reallocating to stepping than standing. However, using different ratios of standing/stepping showed even if only a small amount of time was reallocated to stepping, the favourable associations were much stronger. | | | | | | | | | | | | | |
| Matricciani (2020) | Children | Reallocating time to MVPA from any other behaviour (sleep, SB, LPA) showed favourable association for biomarkers. Reallocating time from MVPA to any other behaviour (sleep, SB, LPA) showed unfavourable association for adiposity. Reallocating time from SB or LPA to sleep also showed favourable associations with biomarkers, albeit much weaker than reallocations involving MVPA. | | | | | | | | | | | | | |
| Matricciani (2020) | Adults | Reallocating time to MVPA from any other behaviour (sleep, SB, LPA) showed favourable association for biomarkers. Reallocating time from MVPA to any other behaviour (sleep, SB, LPA) showed unfavourable association for biomarkers. Reallocating time from SB or sleep to LPA also showed favourable associations with adiposity, albeit much weaker than reallocations involving MVPA. | | | | | | | | | | | | | |
| Farrahi (2021) | Adults | Reallocating time to MVPA from any behaviour associated with favourable changes in multiple biomarkers (fasting glucose, LDL/HDL ratio). Reallocating time away from MVPA unfavourable for biomarkers. Reallocations from SB or sleep to LPA also favourable, albeit relationship weaker. | | | | | | | | | | | | | |
| Chronic disease/condition | |  |  |  |  |  |  |  |  |  |  |  |  |  |  |
| Lewthwaite (2019) | Older adults | Breathlessness | 30.00 | 0.09 | 0.02 | 0.04 | 0.06 | -0.02 | -0.03 | 0.07 | -0.02 | 0.02 | -0.08 | -0.09 | -0.06 |
| Lee (2020) | Older adults | Chronic kidney disease (eGFR)^ | 39.00 |  | -1.06 | -0.86 |  |  |  |  | 0.73 | 0.21 |  |  |  |
|  |  |  | 8.00 |  |  |  |  | -0.13 | 0.03 |  |  |  |  |  |  |
| Lee (2020) | Older adults | Chronic kidney disease (UACR)^ | 39.00 |  | 14.27 | 0.53 |  |  |  |  | -10.27 | -13.85 |  |  |  |
|  |  |  | 8.00 |  |  |  |  | 14.37 | 12.14 |  |  |  |  |  |  |
| Walmsley (2021) | Adults | Incident CVD^#^ | 60.00 |  | 1.04 * |  |  |  |  |  | 0.96 * |  |  |  |  |
|  |  |  | 15.00 |  |  |  |  | 1.19 * |  |  |  | 0.92 * |  |  |  |
| Yerramalla (2021) | Adults (MVPA = 10 min/day) | incident CVD^#^ | 10.00 |  | 1 | 0.87 * |  |  |  |  | 1 | 0.87 * |  |  |  |
|  | Adults (MVPA = 21 min/day) |  | 10.00 |  | 1 | 0.93 * |  | 1.14 * | 1.14 * |  | 1 | 0.92 * |  |  |  |
|  | Adults (MVPA = 30 min/day) |  | 10.00 |  | 1 | 0.94 |  | 1.09 * | 1.08 * |  | 1 | 0.94 * |  |  |  |
| Tsunoda (2021) | Adults | NAFLD^ | 60.00 |  | 0.99 | 0.78 * |  | 1.69 * | 1.69 * |  | 0.99 | 0.78 * |  |  |  |
| Cognitive health |  |  |  |  |  |  |  |  |  |  |  |  |  |  |  |
| Kuzik (2020) | Young children | Cognitive self-regulation | 30.00 |  |  | 0.22 * |  |  | -0.25 * |  |  |  |  |  |  |
| Whitaker (2021) | Adults (female) | DSST | 30.00 |  |  | 0.02 |  |  |  |  | -0.01 | 0.01 |  |  |  |
|  | Adults (male) | DSST | 30.00 |  |  | 0.07 * |  |  |  |  | -0.05 * | 0.03 |  |  |  |
| Bezerra (2020) | Young children | Executive function | 20.00 | 0.78 | 0.07 | 1.43 * | -0.73 * | -0.85 | -0.81 | -0.08 | -0.74 | 0.53 | -0.66 * | -1.46 * | 0.51 * |
| Fairclough (2021) | Children (primary school sample) | Inhibition errors | 20.00 | -5.45 * | -4.59 * | -8.23 * |  |  | 9.45 * |  | 4.19 * |  |  | 5.09 * |  |
| Whitaker (2021) | Adults (female) | RAVLT | 30.00 |  |  | -0.01 |  |  |  |  | -0.01 | -0.01 |  |  |  |
|  | Adults (male) | RAVLT | 30.00 |  |  | 0.09 * |  |  |  |  | -0.03 * | 0.07 * |  |  |  |
| Migueles (2020) | Children (overweight sample) | Right hippocampal volume | 45.00 |  | -141 |  |  |  |  |  | 146 |  |  |  |  |
|  |  |  | 30.00 |  |  |  |  | 121 |  |  |  | -29 |  |  |  |
|  | Children (obese 1 sample) | Right hippocampal volume | 45.00 |  | 44 |  |  |  |  |  | -39 |  |  |  |  |
|  |  |  | 30.00 |  |  |  |  | -272 * |  |  |  | 150 * |  |  |  |
|  | Children (obese2 sample) | Right hippocampal volume | 45.00 |  | 36 |  |  |  |  |  | -46 |  |  |  |  |
|  |  |  | 30.00 |  |  |  |  | 60 |  |  |  | -51 |  |  |  |
| Whitaker (2021) | Adults (female) | Stroop | 30.00 |  |  | 0.05 |  |  |  |  | 0.01 | 0.06 * |  |  |  |
|  | Adults (male) | Stroop | 30.00 |  |  | -0.11 * |  |  |  |  | 0.05 * | -0.06 |  |  |  |
| Fairclough (2021) | Children (primary school sample) | Switching errors | 20.00 | -2.15 * | -2.43 * | -3.42 * |  |  | 3.84 * |  | 2.26 * |  |  | 1.97 * |  |
| Kuzik (2020) | Young children | Vocabulary | 30.00 |  |  |  |  |  |  | -1.08 * | -1.11 * |  | 1.03 * |  |  |
| Amagasa (2020) | Older adults | Reallocating time away from MVPA to other behaviours collectively associated with higher risk of being in cognitive decline. Reallocating time towards MVPA from other behaviours associated with lower risk of being in cognitive decline. | | | | | | | | | | | | | |
| Dumuid (2022) | Older adults | Reallocating time to MVPA from other behaviours collectively had strongest favourable association for executive function. Reallocations away from MVPA also most unfavourable. For those carrying the APOE e4 gene the association was twice as large as non-carriers, however CIs did overlap. For example, reallocating 30 min away from MVPA resulted in 0.5 SD and 1.0 SD decrease in executive function for non-carriers vs. carriers respectively. | | | | | | | | | | | | | |
| Machida (2021) | Older adults | Reallocating time to MVPA from other behaviours collectively associated with increased hippocampal volume and reallocating time away from MVPA to other behaviours collectively associated with lower hippocampal volume. For example, reallocating 26.6 min/day away from MVPA associated with 33.1mm^3^ smaller hippocampal volume. | | | | | | | | | | | | | |
| Fitness |  |  |  |  |  |  |  |  |  |  |  |  |  |  |  |
| Lemos (2021) | Young children | 20m shuttle run (laps) | 15.00 |  |  | 1.61 |  |  | -2.04 |  | -0.03 | 1.59 * |  | 0.21 | 1.83 * |
| Zhang (2022) | Adolescents | 50m run (s) | 30.00 | -0.07 * | -0.05 * | -0.22 * | 0.4 * | 0.42 * | 0.47 * | -0.02 | 0.04 * | -0.17 * | 0.02 | 0.07 * | -0.15 * |
| del Pozo-Cruz (2022) | Older adults | 6MWT | 120.00 |  |  |  |  |  |  |  | 2.53 * | 10.39 * |  |  |  |
| Germano-Soares (2021) | Older adults (male) | 6MWT | 15.00 |  |  |  |  |  |  |  | -2 | 21 * |  |  |  |
|  | Older adults (female) | 6MWT | 15.00 |  |  |  |  |  |  |  | -2 | 14 * |  |  |  |
| Zhang (2022) | Adolescents | 800/1000 m running score | 30.00 | 2.31 * | 1.32 * | 4.21 * | -5.95 * | -6.94 * | -8.1 * | 1 * | -1.14 * | 2.9 * | -1.04 * | -2.19 * | 1.85 |
| Carson (2016) | Children/Adolescents | Aerobic fitness score^%^ | 10.00 | -0.041 | -0.059 | -0.004 | -0.027 | -0.05 | 0.018 | 0.001 | 0.001 | 0.001 | -0.001 | 0.001 | 0 |
| Zhang (2022) | Adolescents | Composite physical fitness score | 30.00 | 1.24 * | 0.6 * | 2.44 * | -3.73 * | -4.37 * | -4.88 * | 0.65 * | -0.5 * | 1.85 * | -0.67 * | -1.18 * | 1.18 |
| del Pozo-Cruz (2022) | Older adults | CST Number of cycles | 120.00 |  |  |  |  |  |  |  | 0.61 * | 1.81 * |  |  |  |
| del Pozo-Cruz (2022) | Older adults | Hand Grip (Kg) | 120.00 |  |  |  |  |  |  |  | 0.68 | 2.49 * |  |  |  |
| Zhang (2022) | Adolescents | Long jump (m) | 30.00 | 0.02 * | 0.003 | 0.06 * | -0.1 * | -0.12 * | -0.12 * | 0.02 * | -0.002 | 0.05 * | -0.02 * | -0.02 * | 0.03 |
| Lemos (2021) | Young children | Speed/agility (sec) | 15.00 |  |  | 2.39 |  |  | -2.63 |  | -0.99 | 1.32 |  | -1.52 * | 0.8 |
| Lemos (2021) | Young children | Standing long jump (cm) | 15.00 |  |  | 0.09 |  |  | -0.35 |  | 0.3 | 0.43 |  | 1.31 * | 1.44 |
| del Pozo-Cruz (2022) | Older adults | TUG test | 120.00 |  |  |  |  |  |  |  | -7.63 * | -12.43 * |  |  |  |
| Dumuid (2018) | Older adults | VO2max | 15.00 | -0.1 | 0 | 1 * | -1.9 * | -1.8 * | -1.8 * | -0.1 | 0 | 1.1 * | 0.1 | 0.1 | 1.1 * |
| Fairclough (2017) | Children | VO2max | 15.00 | 0 | 0 | 1.3 | -2.4 | -2.4 | -2.4 | 0 | 0 | 1.3 | 0 | 0 | 1.3 |
|  | Children (underweight) | VO2max | 15.00 | 0 | 0 | 1 | -1.6 | -1.6 | -1.6 | 0 | 0 | 1.1 | 0 | 0 | 1.1 |
|  | Children (normal weight) | VO2max | 15.00 | 0 | 0 | 1.2 | -2.1 | -2.1 | -2.1 | 0 | 0 | 1.2 | 0 | 0 | 1.2 |
|  | Children (Overweight/obese) | VO2max | 15.00 | 0 | 0 | 1.7 | -5.1 | -5.1 | -5 | 0 | 0 | 1.8 | 0 | 0 | 1.8 |
| Oviedo-Caro (2020) | Adults | VO2max^%^ | 30.00 | -0.28 * | -0.08 | 0.31 | -0.79 * | -0.59 * | -0.52 * | -0.2 | 0.07 | 0.39 * | 0.21 | 0.29 * | 0.6 * |
| Migueles (2022) | Young children | Reallocating time to VPA from other behaviours (SB, LPA, MPA) individually or collectively associated with increased upper and lower body strength and motor fitness. Results suggest that behaviour/s reallocated from were irrelevant, as long as VPA was increased. | | | | | | | | | | | | | |
| General/perceived health | |  |  |  |  |  |  |  |  |  |  |  |  |  |  |
| Lewthwaite (2019) | Older adults | Fatigue | 30.00 | 0.06 | 0.05 | 0.09 | -0.05 | -0.07 | -0.11 | 0.02 | -0.04 | 0.04 | -0.02 | -0.06 | 0.03 |
| Verhoog (2020) | Adults/Older adults | HRQoL | 30.00 | 0.01 | 0.01 | 0.04 * | -0.04 * | -0.04 * | -0.05 * | -0.001 | -0.01 | 0.03 * | 0.002 | -0.01 | 0.03 * |
| Lewthwaite (2019) | Older adults | Mastery | 30.00 | 0.11 | 0.02 | 0.12 | -0.04 | -0.13 | -0.15 | 0.09 | -0.01 | 0.1 | -0.09 | -0.11 | 0 |
| Curtis (2020) | Adults | Physical HRQoL | 15.00 | 0.02 | 0.04 * | 0.09 * | -0.08 * | -0.07 * | -0.1 * | -0.01 | -0.03 * | 0.05 * | 0.01 | -0.02 | 0.07 * |
| Verhoog (2020) | Adults/Older adults | VAS | 30.00 | 0.86 | 1.05 | 3.05 * | -3.16 * | -2.97 * | -3.84 * | -0.19 | -0.86 | 2 * | 0.2 | -0.66 | 2.2 * |
| Dumuid (2018) | Children | Reallocating time to sleep or MVPA from other behaviours collectively generally favourable for HRQoL. Reallocating time to SB and LPA from other behaviours collectively generally unfavourable for HRQoL. Children from high human development index countries reported higher association between reallocating time toward MVPA and favourable HRQoL than those from lower human development index countries. | | | | | | | | | | | | | |
| Ng (2021) | Children | Reallocating time to MVPA from other behaviours collectively 2-6 times more potent than reallocations to sleep or away from SB (from other behaviours collectively) in terms of higher HRQoL. Reallocating time to LPA unfavourable for all outcomes. | | | | | | | | | | | | | |
| Mental health | |  |  |  |  |  |  |  |  |  |  |  |  |  |  |
| Lewthwaite (2019) | Older adults | Anxiety | 30.00 | 0.06 | -0.01 | 0.06 | -0.02 | -0.09 | -0.07 | 0.07 | 0.02 | 0.07 | -0.07 | -0.06 | -0.01 |
| Kandola (2021) | Adults | Anxiety score^%^ | 60.00 |  |  |  |  |  |  | -4.5 * | 4.5 * | -6.6 * |  |  |  |
| Chao (2022) | Adults | Anxiety symptoms | 15.00 | 0.2 * | 0.2 * | -0.03 | 0.78 * | 0.78 * | 0.93 * | 0 | -0.12 * | -0.22 * | 0 | -0.12 * | -0.23 * |
| Hofman (2022) | Adults/Older adults | Anxiety symptoms | 30.00 | -0.19 | -0.2 | -0.32 | 0.19 | 0.18 | 0.35 | 0.01 | 0.17 | -0.12 | -0.01 | 0.16 | -0.13 |
|  | Adults/Older adults (male) | Anxiety symptoms | 30.00 | -0.1 | -0.1 | -0.17 | 0.11 | 0.11 | 0.19 | 0 | 0.08 | -0.08 | 0.01 | 0.09 | -0.07 |
|  | Adults/Older adults (female) | Anxiety symptoms | 30.00 | -0.27 | -0.31 | -0.46 | 0.28 | 0.24 | 0.5 | 0.04 | 0.26 | -0.15 | -0.04 | 0.22 | -0.2 |
| Hofman (2022) | Adults/Older adults | Clinically relevant anxiety symptoms^ | 30.00 | 0.77 | 0.77 | 0.6 | 1.53 | 1.53 | 1.94 | 1 | 1.26 | 0.77 | 1 | 1.26 | 0.77 |
| Hofman (2022) | Adults/Older adults | Clinically relevant depressive symptoms^ | 30.00 | 1.03 | 1.02 | 0.94 | 1.24 | 1.23 | 1.26 | 1.01 | 1 | 0.9 | 0.99 | 0.99 | 0.89 |
| Cabanas-Sanchez (2021) | Older adults (cross-sectional) | Depression | 30.00 | -0.067 * | -0.08 * | -0.326 * | 0.507 * | 0.494 * | 0.559 * | 0.013 | 0.065 * | -0.246 * | -0.014 | 0.051 | -0.26 * |
|  | Older adults (prospective) | Depression | 30.00 | 0.001 | -0.031 | -0.057 | 0.093 | 0.061 | 0.088 | 0.033 * | 0.028 | -0.026 | -0.033 * | -0.006 | -0.06 |
| Lewthwaite (2019) | Older adults | Depression | 30.00 | 0.01 | 0 | 0.09 | -0.12 | -0.13 | -0.13 | 0 | 0 | 0.08 | 0 | -0.01 | 0.08 |
| Blodgett (2022) | Adults | Depression risk^ | 30.00 | 1.07 * | 1.08 * | 0.76 * | 1.88 * | 1.82 * | 1.75 * | 1.04 | 0.93 * | 0.74 * | 0.95 | 0.93 * | 0.71 * |
| Kandola (2021) | Adults | Depression score^%^ | 60.00 |  |  |  |  |  |  | -7.6 * | -1.3 * | -12.5 * |  |  |  |
| del Pozo Cruz (2020) | Adults | Depressive symptoms | 45.00 |  |  |  |  |  |  |  | -0.009 |  |  |  |  |
|  |  |  | 60.00 |  |  |  |  |  |  | -0.08 * |  | -0.09 * |  |  |  |
| Hofman (2022) | Adults/Older adults | Depressive symptoms | 30.00 | -0.15 | -0.11 | -0.7 | 0.81 * | 0.85 * | 0.93 | -0.04 | 0.08 | -0.59 * | 0.03 | 0.12 | -0.55 * |
|  | Adults/Older adults (male) | Depressive symptoms | 30.00 | -0.23 | -0.18 | -0.84 | 0.9 * | 0.96 * | 1.09 | -0.05 | 0.14 | -0.66 * | 0.05 | 0.19 | -0.61 * |
|  | Adults/Older adults (female) | Depressive symptoms | 30.00 | -0.04 | -0.06 | -0.5 | 0.67 | 0.65 | 0.7 | 0.02 | 0.04 | -0.45 | -0.02 | 0.02 | -0.47 |
| Sampasa-Kanyinga (2021) | Children (younger boys) | Depressive symptoms | 60.00 |  |  |  | -0.033 * |  |  |  |  |  |  |  | 0.031 * |
|  | Children (older boys) | Depressive symptoms | 60.00 |  |  |  | -0.026 * |  |  |  |  |  |  |  | 0.027 * |
|  | Children (younger girls) | Depressive symptoms | 60.00 |  |  |  | -0.049 * |  |  |  |  |  |  |  | 0.044 * |
|  | Children (older girls) | Depressive symptoms | 60.00 |  |  |  | 0.025 * |  |  |  |  |  |  |  | 0 |
| Su (2022) | Adults | Depressive symptoms | 15.00 | 0.01 | 0.06 * | -0.07 | 0.15 * | 0.19 * | 0.14 * | -0.05 * | -0.05 * | -0.13 * | 0.05 * | -0.01 | -0.08 * |
| Brown (2021) | Young children | Externalising problems | 25.00 |  | -0.51 | -0.03 |  | -0.68 | -0.16 |  | 0.51 | 0.57 |  |  |  |
| Cabanas-Sanchez (2021) | Older adults (cross-sectional) | Global mental health | 30.00 | -0.06 | -0.029 | 0.323 | -0.73 | -0.699 | -0.674 | -0.031 | 0.025 | 0.352 | 0.034 | 0.059 | 0.386 |
|  | Older adults (prospective) | Global mental health | 30.00 | 0.236 | 0.276 | 0.755 * | -0.962 * | -0.923 * | -1.15 * | -0.04 | -0.228 | 0.479 * | 0.041 | -0.186 | 0.521 * |
| Cabanas-Sanchez (2021) | Older adults (cross-sectional) | Happiness | 30.00 | 0.02 | 0.014 | 0.172 * | -0.307 * | -0.313 * | -0.323 * | 0.006 | -0.01 | 0.158 * | -0.006 | -0.016 | 0.152 * |
|  | Older adults (prospective) | Happiness | 30.00 | 0.023 | 0.024 | 0.077 | -0.101 | -0.1 | -0.119 | -0.001 | -0.02 | 0.053 | 0.001 | -0.018 | 0.054 |
| Le (2022) | Adults | High arousal negative affect | 15.00 | -0.01 | 0 | 0 | -0.01 | 0 | 0 | -0.01 | 0 | 0 | 0.01 | 0.01 | 0.01 |
| Le (2022) | Adults | High arousal positive affect | 15.00 | 0.01 | 0 | 0.03 * | -0.03 * | -0.04 * | -0.04 * | 0.01 | 0 | 0.04 * | 0 | 0 | 0.03 * |
| Brown (2021) | Young children | Internalising problems | 25.00 |  | -0.26 | -0.88 |  | 0.96 | 1.17 |  | 0.25 | -0.63 |  |  |  |
| Fairclough (2021) | Children/Adolescents | Internalising problems | 20.00 |  | -0.03 |  |  | 0.69 * |  | -0.11 * | 0.02 | -0.44 * | 0.11 * |  |  |
| Cabanas-Sanchez (2021) | Older adults (cross-sectional) | Loneliness | 30.00 | -0.045 | -0.045 | -0.118 * | 0.151 * | 0.15 * | 0.187 * | 0 | 0.036 | -0.073 * | 0 | 0.036 | -0.074 * |
|  | Older adults (prospective) | Loneliness | 30.00 | -0.032 | -0.034 | -0.081 | 0.093 | 0.091 | 0.119 | 0.002 | 0.028 | -0.047 | -0.002 | 0.026 | -0.05 |
| Larisch (2020) | Adults | Mental wellbeing | 60.00 |  |  | 5 * | -21 * | -21 * | -22 * |  |  | 5 * |  |  | 5 |
| Fairclough (2021) | Children (primary school sample) | Prosocial behaviour | 20.00 |  | -0.06 * |  |  | -0.19 * |  | 0.12 * | 0.07 * | 0.15 * | -0.12 * |  |  |
| Carson (2016) | Children/Adolescents | SDQ^%^ | 10.00 | -0.171 | 0.072 | -0.024 | 0.087 | 0.636 | 0.25 | -0.008 | -0.002 | -0.005 | 0.007 | 0.003 | -0.001 |
| Ren (2022) | Adolescents | Smartphone addiction | 8.00 | -0.07 | 0.01 | -0.28 | 0.78 | 0.85 | 0.85 | -0.08 | -0.01 | -0.3 | 0.08 | 0.07 | -0.18 |
| Kuzik (2020) | Young children | Sociability | 30.00 |  |  |  | -0.21 * | -0.21 * | -0.26 * |  |  | 0.16 * |  |  | 0.16 * |
| Mortality |  |  |  |  |  |  |  |  |  |  |  |  |  |  |  |
| Clarke (2021) | Adults | Mortality^#^ | 75.00 |  |  | 0.68 * |  |  |  |  |  | 0.61 * |  |  | 0.64 * |
|  |  |  | 90.00 | 1.07 * |  |  |  |  |  | 0.93 |  |  |  |  |  |
|  |  |  | 120.00 |  | 1.19 * |  |  |  |  |  | 0.84 * |  | 1.08 | 0.93 * |  |
|  |  |  | 15.00 |  |  |  | 1.78 * | 1.8 * | 1.76 * |  |  |  |  |  |  |
| McGregor (2019) | Adults/Older adults | Mortality^#^ | 30.00 | 1.04 | 1.06 | 0.85 |  |  |  | 0.99 | 0.95 | 0.79 | 1.02 | 0.97 | 0.81 |
|  |  |  | 8.00 |  |  |  | 1.26 | 1.26 | 1.26 |  |  |  |  |  |  |
| Von Rosen (2019) | Adults/Older adults | Mortality^#^ | 45.00 |  | 1.15 | 0.99 |  |  |  |  | 0.87 | 0.86 |  |  |  |
|  |  |  | 20.00 |  |  |  |  | 1.26 * | 1.23 |  |  |  |  |  |  |
| Chastin (2021) | Adults/Older adults | When reallocating time from different reference compositions the association of a given time reallocation varies. To reduce HR to 0.9 reallocating 8min, 29min and 52 min from SB to MVPA needed using reference compositions (2, 10 and 30min/day of MVPA respectively). Reallocating SB to LPA also associated with reduced risk of mortality. | | | | | | | | | | | | | |
| Motor/movement skills |  |  |  |  |  |  |  |  |  |  |  |  |  |  |  |
| Estevan (2022) | Children (timepoint 1) | Actual ball skills | 10.00 |  | 0.53 * | 0.99 * |  | -0.56 * | -1.08 * |  | -0.52 * | 0.46 * |  |  |  |
|  | Children (timepoint 2) |  | 10.00 |  | 0.37 * | 0.52 * |  | -0.2 | -0.56 * |  | -0.37 * | 0.15 |  |  |  |
| Estevan (2022) | Children (timepoint 1) | Actual locomotion | 10.00 |  | 0.3 * | 0.61 * |  | -0.38 * | -0.67 * |  | -0.29 * | 0.31 * |  |  |  |
|  | Children (timepoint 2) |  | 10.00 |  | 0.2 * | 0.57 * |  | -0.45 * | -0.64 * |  | -0.19 * | 0.37 * |  |  |  |
| Estevan (2022) | Children (timepoint 1) | Actual MC | 10.00 |  | 0.83 * | 1.61 * |  | -0.94 * | -1.74 * |  | -0.81 * | 0.78 * |  |  |  |
|  | Children (timepoint 2) |  | 10.00 |  | 0.57 * | 1.09 * |  | -0.65 * | -1.21 * |  | -0.56 * | 0.52 * |  |  |  |
| Smith (2020) | Children (total sample) | FMS | 60.00 | 1.5 | -0.2 | 1.22 * | -0.16 | -1.85 * | -1.52 | 1.88 * | 0.52 | 1.6 * | -1.76 * | -1.43 | -0.35 |
|  | Children (British sample) |  | 60.00 | 1.38 | -0.4 | 1.2 * | -0.69 | -2.47 * | -1.97 | 2.01 * | 0.72 * | 1.82 * | -1.85 * | -1.35 | -0.26 |
|  | Children (Iranian sample) |  | 60.00 | 1.63 | -0.01 | 1.28 * | 0.09 | -1.56 * | -1.37 * | 1.81 * | 0.35 | 1.45 * | -1.71 * | -1.53 | -0.43 |
| Kuzik (2020) | Young children | Locomotor MS | 30.00 |  | 1.94 * | 3.28 * | -2.79 * |  | -3.82 * |  | -1.88 * |  |  |  | 2.12 * |
| Mota (2020) | Young children | Locomotor MS | 20.00 | 0.3 | 0.42 | 0.65 | -0.47 | -0.35 | -0.74 | -0.13 | -0.4 | 0.23 * | 0.13 | -0.27 | 0.36 |
| Kuzik (2020) | Young children | Object control MS | 30.00 |  |  | 3.99 * | -3.54 * | -3.67 * | -4.79 * |  |  | 2.75 * |  |  | 2.62 * |
| Mota (2020) | Young children | Object control MS | 20.00 | 0.56 * | 0.78 * | 1.15 * | -0.79 | -0.57 * | -1.3 * | -0.23 * | -0.74 * | 0.36 | 0.22 * | -0.51 * | 0.59 |
| Estevan (2022) | Children (timepoint 1) | Perceived ball skills | 10.00 |  | -0.1 | -0.02 |  | -0.08 | 0.01 |  | 0.1 | 0.08 |  |  |  |
|  | Children (timepoint 2) |  | 10.00 |  | 0.01 | 0.18 |  | -0.2 | -0.21 |  | -0.01 | 0.17 |  |  |  |
| Estevan (2022) | Children (timepoint 1) | Perceived locomotion | 10.00 |  | -0.08 * | -0.06 |  | -0.02 | 0.06 |  | 0.08 * | 0.02 |  |  |  |
|  | Children (timepoint 2) |  | 10.00 |  | 0.03 | 0.3 * |  | -0.32 * | -0.35 * |  | -0.03 | 0.27 * |  |  |  |
| Estevan (2022) | Children (timepoint 1) | Perceived MC | 10.00 |  | -0.17 * | -0.08 |  | -0.1 | 0.07 |  | 0.17 * | 0.1 |  |  |  |
|  | Children (timepoint 2) |  | 10.00 |  | 0.04 | 0.48 * |  | -0.52 * | -0.56 * |  | -0.04 | 0.44 * |  |  |  |
| Kuzik (2020) | Young children | Total MS | 30.00 |  | 3.18 * | 7.27 * | -6.33 * | -5.67 * | -8.62 * |  | -2.99 * | 4.03 * |  |  | 4.74 * |
| Mota (2020) | Young children | Total MS | 20.00 | 0.86 * | 1.2 * | 1.81 * | -1.26 | -0.92 | -2.04 * | -0.33 * | -1.14 * | 0.59 | 0.35 * | -0.78 * | 0.95 |
| Other |  |  |  |  |  |  |  |  |  |  |  |  |  |  |  |
| del Pozo-Cruz (2022) | Older adults | Barthel Index | 120.00 |  |  |  |  |  |  |  | 2.53 * | 10.42 * |  |  |  |
| Bianchim (2022) | Children | Lung capacity (FEV1% predicted) | 30.00 | 2.56 * | -0.73 * | 0.51 | 2.17 * | -1.12 * | -0.36 | 3.42 * | 0.89 * | 1.37 * | -3.41 * | -2.65 * | -2.18 * |
| Bianchim (2022) | Adults | Lung capacity (FEV1% predicted) | 30.00 | -2.94 * | -1.84 * | -0.8 * | -2.33 * | -1.23 * | 0.53 * | -1.06 * | 1.8 * | 1.08 * | 1.2 | 2.96 * | 2.24 |
| Taylor (2018) | Young children | Reallocating time to MVPA from other behaviours collectively favourable for bone mineral density at multiple ages. Other reallocations generally not related. However, size of all associations very small. | | | | | | | | | | | | | |

Values represent change in outcome, unless otherwise indicated. * indicates result significant at p <0.05; ^%^ indicates percent change in outcome; ^#^ indicates HR for outcome; ^^^ indicates OR for outcome.

Abbreviations: APOE, apolipoprotein E; BMI, body mass index; BP, blood pressure; CRP, C-reactive protein; CMR, cardio-metabolic risk; CVD, cardio-vascular disease; DSST, digit symbol substitution test; eGFR, estimated glomerular filtration rate; FMI, fat mass index; FFMI, fat-free mass index; FMS, fundamental motor skills; FEV1%, forced expiratory volume in 1 second; HbAlc, hemoglobin A1c; HDL, high-density lipoprotein; HOMA-IR, Homeostatic Model Assessment for Insulin Resistance; HOMA-IS, Homeostasis Model Assessment of Insulin Sensitivity; HRQoL, health-related quality of life; IGFBP, insulin-like growth factor-binding protein; LPA, light physical activity; Matsuda-ISI, matsuda insulin sensitivity index; MC, motor competence; MetS, metabolic syndrome score; MS, motor skills; MVPA, moderate-to-vigorous physical activity; NAFLD, non-alcoholic fatty liver disease; RAVLT, rey auditory verbal learning test; SB, sedentary behaviour; SD, standard deviation; SDQ, strength and difficulties questionnaire; TUG, timed up and go; UACR, urine albumin-creatinine ratio; VPA, vigorous physical activity; WC, waist circumference; zBMI, body mass index z-score; %BF, body fat percentage; %WHtR, waist-to-height ratio
