# Supplementary material for: The association between reallocations of time and health using compositional data analysis: a systematic scoping review with an interactive data exploration interface
Source: Int J Behav Nutr Phys Act. 2023 Oct 19;20:127. doi: 10.1186/s12966-023-01526-x (PMC10588100; doi:10.1186/s12966-023-01526-x)
Supplement: Supplementary file 8 — Supplementary Material 8: Table S7. Findings for studies reallocating time during subcompositions of the day [file 12966_2023_1526_MOESM8_ESM.docx]

Table S7. Findings for studies reallocating time during subcompositions of the day

| Study ID | Study sample | Type of reallocation | Time-use components | Findings |
| --- | --- | --- | --- | --- |
| Atkin (2021) | Adolescents | 1-for-1, 1-for-remaining | Sleep, PA, School-related, Hobbies & socialising, Electronic media, Domestic, personal care, work-related (on weekends and weekdays separately) | Reallocating time to PA generally favourable for depressive symptoms, global self-worth and strengths and difficulties questionnaire regardless of where time came from. Reallocations were done separately for weekdays and weekend days. Predicted differences were slightly larger for the weekend analysis than the weekday analysis, but the magnitude of change was relatively small in either instance (less than 0.3 of a unit). Reallocating time away from screen time towards other domains also favourable for self-worth and depressive symptoms. However, predicted differences all less than 0.1 units. |
| Bourke (2022) | Young Children | 1-for-1 | Preschool SB, Preschool LPA, Preschool MVPA | Reallocating time to LPA from either SB or MVPA favourable for physical functioning score (2min/hr associated with 1.51, 2.97 change respectively). Reallocating time away from LPA unfavourable. Non-significant trend for reduced zBMI when reallocating time to MVPA. |
| Burns (2019) | Children | 1-for-1 | School SB, School LPA, School MVPA | Reallocating time from school LPA to school MVPA resulted in more pronounced improvements in motor skills than reallocating from school SB to school MVPA. A 5% reallocation in percentage of time spent in LPA to MVPA was associated with a 1.74-point increase in TGMD-3 scores. The reallocation of SB to MVPA was less pronounced. |
| Fairclough (2018) | Children | 1-for-1 | School SB, School LPA, School MVPA | Reallocating 10min of school time from MVPA to SB associated with higher adiposity (0.16, 0.28, unit increase in zBMI and %WHtR respectively) and lower VO2 max (-0.91). changes were even more pronounced when reallocating MVPA with LPA. The reverse was true when SB or LPA were reallocated for MVPA, but the magnitude of the predicted differences was smaller. |
| Gaba (2021) | Children, Adolescents | 1-for-1 | Sleep, School SB, Out-of-school SB, School LPA, Out-of-school LPA, School MPA, Out-of-school MPA, School VPA, Out-of-school VPA | Reallocating out-of-school SB to out-of-school LPA results in favourable changes in adiposity among girls (30 min reallocation associated with 14% lower FMI). No significant associations were found for school SB. |
| Giurgiu (2022) | Adults | 1-for-remaining | SB, LPA, MVPA (within hour windows of the day) | Reallocating time to MVPA and LPA (relative to remaining) positively associated with mood (energetic arousal and valence). Reallocating time to SB from remaining behaviours negatively associated with mood. |
| Gupta (2020) | Adults | 1-for-remaining | Sleep, Work SB, Work Standing, Work LPA, Work MVPA, Leisure SB, Leisure Standing, Leisure LPA, Leisure MVPA | Reallocating 20 min to MVPA at work from other work behaviours was significantly positively associated with LTSA (HR 1.15), while 20 min more time spent on MVPA in leisure, relative to other leisure behaviours, was significantly negatively associated with LTSA (HR 0.80). |
| Gupta (2019) | Adults | 1-for-remaining | Sleep, Work SB, Work LPA, Work MVPA, Leisure SB, Leisure LPA, Leisure MVPA | Reallocating 30min to SB during work or leisure time (from other domain specific behaviours collectively) associated with unfavourable associations for systolic BP (0.7, 0.5 unit increase respectively). Only leisure time SB reallocations associated with diastolic BP. During leisure, it seems beneficial to reallocate time from SB to remaining behaviours, especially to time in bed and MVPA |
| Gupta (2022) | Adults | 1-for-1, 1-for-remaining | Work SB, Work standing, Work LPA, Work MVPA, Leisure SB, Leisure standing, Leisure LPA, Leisure MVPA, Time in bed | For employees with low back pain reallocating 20min/day to work MVPA from other work behaviours associated with higher LTSA risk (HR 1.38), however, reallocating 20min/day to work LPA from other work behaviours associated with lower LTSA risk (HR 0.82). No such associations were found for those without low back pain. During leisure, reallocating time to MVPA from other behaviours was associated with reduced LTSA. |
| Hallman (2021) | Adults | 1-for-remaining | Work sitting, Work standing, Work LPA, Work MVPA, Leisure MVPA, Leisure non-MVPA | Latent class analysis identified four trajectories for change in sick leave. Reallocating time to work SB from all other reduced likelihood of being in few days - increasing trajectory group. Reallocating time to work LPA from other work time predicted increase in likelihood of increasing |
| Januario (2020) | Adults | 1-for-1, 1-for-remaining | Work sitting, Work standing, Work LPA, Work MVPA | Reallocating 30 min to sitting relative to remaining work behaviours associated with less perceived physical exertion, while reallocating 30 min to work MVPA relative to remaining work behaviours were associated with more perceived physical exertion (0.11). in 1-for-1 reallocations reallocating work MVPA to work SB showed strongest associations with lower exertion. However, ESs for all reallocations were small. |
| Johansson (2022) | Adults | 1-for-1 | Sleep, Work SB, Work Standing, Work Moving, Work Walking, Work HIPA, Leisure SB, Leisure Standing, Leisure Moving, Leisure Walking, Leisure HIPA | During leisure, reallocating time away from SB to either walking or high-intensity PA (HIPA) associated with a lower SBP, while during work, the findings indicated an association with a higher SBP, although non-significant. During both work and leisure, reallocating time from less SB to HIPA was associated with a smaller WC and a lower LDL cholesterol. |
| Ketels (2020) | Adults | 1-for-1, 1-for-remaining | Sleep, Work SB, Work Standing, Work LPA, Work MVPA, Leisure SB, Leisure standing, Leisure LPA, Leisure MVPA | Reallocating time to work SB from other work behaviours was associated with higher CRF, while reallocating leisure time to SB from other leisure behaviours associated with lower CRF. Reallocating time to MVPA during work, relative to the other work behaviours, was not significantly associated with lower CRF levels, while reallocating time to MVPA during leisure time was significantly associated with higher CRF levels. No associations found for reallocations involving standing and LPA in either work or leisure. |
| Kitano (2022) | Adults | 1-for-1 | Work SB, Work LPA, Work MVPA, Leisure SB, Leisure LPA, Leisure MVPA | During leisure time, reallocating time from SB to either LPA or MVPA were associated with favourable changes in cardiometabolic risk score (CmRS) and lipid metabolism, respectively. For example, reallocating 30 min/day from SB to LPA was associated with a decrease in CmRS by 0.03 points, corresponding to a 13.2% decrease. Reallocating 30 min/day of leisure SB to leisure MVPA was also associated with lower triglycerides (-3.16 mg/dl), while opposite reallocation was associated with higher triglycerides (5.26 mg/dl). Similar results were also observed for HDL in relation to leisure time reallocations. Reallocating 30 min/day of work SB to work LPA was associated with higher diastolic SB (0.39 mmHg), but during leisure, this time-substitution was unfavourable (-0.47 mmHg). |
| Kitano (2020) | Adults | 1-for-1 | Sleep, SB, LPA, MVPA (on workdays and non-workdays) | On workdays, reallocating time to Sleep from either SB or LPA associated with reduced probability of psychological distress. On workdays, reallocating time from SB to sleep reduced probability of work engagement. Reallocations involving MVPA not reported. |
| Ma (2021) | Adults | 1-for-1 | SB, LPA, Morning MVPA, Evening MVPA | Reallocating SB time to either morning or evening MVPA was associated with a lower risk of all-cause mortality. However, reallocating evening MVPA with morning MVPA was not associated with changes in the risk of all-cause mortality. |
| Martins (2021) | Young children | 1-for-1 | Preschool SB, Preschool LPA, Preschool MVPA | Reallocating preschool time from LPA to SB associated with increased manipulative skills (0.14, 0.28, and 0.42-units; five, 10 and 15 minutes, respectively). All other reallocations not significant. |
| Roscoe (2021) | Young children | 1-for-1 | SB, LPA, MVPA (reallocations modelled on weekdays, weekend days, and combined) | Associations varied depending on whether they were modelled for the whole 4-day monitoring period, or weekends or weekdays separately. For weekday and 4-day reallocations, increasing LPA at the expense of any behaviour was associated with significant improvements in total motor competence and the locomotor and object control subsets. However, for weekend-derived behaviours, MVPA was preferential to LPA, whilst the association between reallocating time for MVPA and SB was equivocal for week-, weekend-, and 4-day-derived behaviours, respectively |
| Stevens (2019) | Adults | 1-for-1, 1-for-remaining | Work SB, Work standing, Work LPA, Work MVPA, Non-work time | Significant, but small reductions in NFR when reallocating 30 min to work SB from other work behaviours (-0.01 unit). Also, despite a lack of significant differences in other reallocations, there was a trend towards increased time spent in more vigorous behaviours being associated with an increased NFR. However, the effect size was very small for all reallocations. |

Abbreviations: CmRS, cardio-metabolic risk score; CRF, cardio-respiratory fitness; FMI, fat mass index; HDL, high-density lipoprotein; HR, hazard ratio; LDL, low-density lipoprotein; LPA, light physical activity; LTSA, long-term sickness absence; MVPA, moderate-to-vigorous physical activity; NFR, need for recovery; PA, physical activity; SB, sedentary behaviour; TGMD, test of gross motor development; VO2, volume of oxygen; WC, waist circumference; WHtR, waist-to-height ratio; zBMI, body mass index z-score
