# Supplementary material for: The association between reallocations of time and health using compositional data analysis: a systematic scoping review with an interactive data exploration interface
Source: Int J Behav Nutr Phys Act. 2023 Oct 19;20:127. doi: 10.1186/s12966-023-01526-x (PMC10588100; doi:10.1186/s12966-023-01526-x)
Supplement: Supplementary file 9 — Supplementary Material 9: Table S8. Results for studies reporting reallocations between recall domains [file 12966_2023_1526_MOESM9_ESM.docx]

Table S8. Results for studies reporting reallocations between recall domains

| Study ID | Study sample | Type of reallocation | Time-use components | Findings |
| --- | --- | --- | --- | --- |
| Atkin (2021) | Adolescents | 1-for-1, 1-for-remaining | Sleep, Physical activity, School-related, Hobbies & socialising, Electronic media, Domestic, personal care, work-related | Reallocating time to PA generally favourable for depressive symptoms, global self-worth and strengths and difficulties (SDQ) questionnaire regardless of where time came from. Reallocations were done separately for weekdays and weekend days. Predicted differences were slightly larger for the weekend analysis than the weekday analysis, but the magnitude of change was relatively small in either instance (less than 0.3 of a unit). Reallocating time away from screen time towards other domains also favourable for self-worth and depressive symptoms. However, predicted differences all less than 0.1 units. |
| Chong (2022) | Children, Adolescents | 1-for-remaining | Sleep, Physical activity, Self-care/domestic, Social, Education, Recreational screen use, Quiet time, Passive transport | Overall, minimal association found for reallocation of behaviour. For boys, reallocating 30 min/day to social time or recreational screen time from other behaviours collectively, resulted in unfavourable changes in prosocial behaviour (-0.16 and -0.03, respectively). No associations found for reallocations of time with girls. |
| Dumuid (2022) | Children | Other | Sleep, Screen time, Physical activity, Quiet time, Passive transport, School-related activity, Domestic/self-care | Interactive app allows people to specify hypothetical changes based on different reference compositions and preferred reallocations, not just 1-for-1 swaps. Reallocations have flow-on effects, where certain reallocations may be good for one outcome, but bad for another. Strength of relationship depends on starting composition, which can be changed by user to allow for personalised predictions. |
| Kandola (2022) | Adolescents | 1-for-1 | Daily screen time, Exercise, Watching TV, Using social media, Playing video games, General computer use, Individual exercise, team sports | Reallocating screen time to exercises associated with lowered emotional symptoms (60min/day reallocation, -0.05). When considering specific screen time and exercise types, reallocating TV viewing and social media with team sports was associated with lower emotional symptoms (-0.17, -0.15 respectively); replacing general computer or gaming not associated with lower symptoms. |
| Lewthwaite (2019) | Older adults | 1-for-1, 1-for-remaining | Sleep, Sedentary behaviour, LPA, MVPA;  Physical activity, Chores, Self-care, Socio-cultural, Passive transport, Work/study, Quiet time | Reallocating time to higher intensity PA or sleep was associated with favourable changes in breathlessness symptoms and HRQoL. Reallocating time to LPA or SB was associated with unfavourable changes in breathlessness symptoms and HRQoL; and reallocating time to specific activity types of self-care, passive transport and work/study was unfavourably associated with breathlessness symptoms and HRQoL. |
| Olds (2018) | Adults | 1-for-1 | Sleep, Physical activity, Chores, Quiet time, Screen time, Self-care, Social time, Passive transport, Work | Longitudinal reallocations after retirement showed that reallocating work to other domains associated with effect sizes ranging from -0.15 to +0.31 for 60-min substitutions. The most favourable substitutions were replacing Work with Physical Activity or Sleep. Unfavourable substitutions showed lower effect sizes, and usually involved replacing Work with Screen Time or Social. Strongest relationships when work substituted for PA on DASS total score, stress, and anxiety. |

Abbreviations: DASS, depression, anxiety and stress scale; HRQoL, health related quality of life; LPA, light physical activity; MVPA, moderate-to-vigorous physical activity; PA, physical activity; SB, sedentary behaviour; SDQ, strengths and difficulties questionnaire.
