# Supplementary material for: The association between reallocations of time and health using compositional data analysis: a systematic scoping review with an interactive data exploration interface
Source: Int J Behav Nutr Phys Act. 2023 Oct 19;20:127. doi: 10.1186/s12966-023-01526-x (PMC10588100; doi:10.1186/s12966-023-01526-x)
Supplement: Supplementary file 10 — Supplementary Material 10: Table S9. Findings from studies reporting reallocations for bouts of activities [file 12966_2023_1526_MOESM10_ESM.docx]

Table S9. Findings from studies reporting reallocations for bouts of activities

| Study ID | Study sample | Type of reallocation | Time-use components | Findings |
| --- | --- | --- | --- | --- |
| Gaba (2020) | Children | 1-for-1 | SB, LPA, MVPA, SB short bouts, SB middle bouts, SB long bouts | Reallocating 2hr/week from middle sedentary bouts to MVPA were associated with favourable adiposity changes ( -5.6%, -6.5%, -11.5% reduction for fat mass %, FMI, visceral adipose tissue respectively). Reallocating time from MVPA to either long or middle bouts of SB associated with unfavourable changes in all markers of adiposity. Results for total SB were similar in magnitude, but not always significant. |
| Gaba (2021) | Older adults | 1-for-1 | SB, LPA, MVPA, SB short bouts, SB middle bouts, SB long bouts | Reallocating time from long bouts of SB to LPA favourable for changes in (30 min reallocation, -3.13 FMI). |
| Rubin (2022) | Children, Adolescents | 1-for-1 | SB, LPA, MPA, VPA, SB short bouts, SB middle bouts, SB long bouts | Reallocating 15min/week from SB towards VPA associated with favourable changes in visceral adipose tissue (-3.8%). Favourable, but non-significant changes observed for other adiposity markers (%BF and FMI). Similar associations generally observed regardless of whether time reallocated from total SB time, or SB accrued in bouts of different lengths. |
| Verswijveren (2022) | Children | 1-for-1 | SB, LPA, MPA, VPA, SB short bouts (<5 min), SB long bouts (≥5 min), LPA short bouts (<1 min), LPA longs bouts (≥1 min), MPA short bouts (<1min), MPA long bouts (≥1 min), VPA short bouts (<1min), VPA long bouts (≥1 min), Other (sleep & non-wear) | Reallocating 10 minutes from sporadic LPA to LPA accrued in a continuous bout associated with 0.2 higher mean zBMI and 2.2 cm higher mean WC in girls. Similar estimations were predicted for boys |

Abbreviations: FMI, fat mass index; LPA, light physical activity; MVPA, moderate-to-vigorous physical activity; SB, sedentary behaviour; VPA, vigorous physical activity; WC, waist circumference; zBMI, body mass index z-score
